# Diverse *N*‐Oxidation of Primary Aromatic Amines Controlled by Engineered P450 Peroxizyme Variants Facilitated by Dual‐Functional Small Molecule

**DOI:** 10.1002/advs.202412100

**Published:** 2024-12-16

**Authors:** Jie Chen, Fuquan Yao, Yiping Jiang, Xiangquan Qin, Mo Xian, Yingang Feng, Zhiqi Cong

**Affiliations:** ^1^ Key Laboratory of Photoelectric Conversion and Utilization of Solar Energy Qingdao New Energy Shandong Laboratory CAS Key Laboratory of Biofuels Shandong Provincial Key Laboratory of Synthetic Biology Qingdao Institute of Bioenergy and Bioprocess Technology Chinese Academy of Sciences Qingdao 266101 China; ^2^ University of Chinese Academy of Sciences Beijing 100049 China

**Keywords:** amine, cytochrome P450 enzymes, enzyme catalysis, *N*‐oxygenation, one‐electron oxidation

## Abstract

Amine oxidation is an important organic reaction for the production of high‐value *N*‐containing compounds. However, it is still challenging to control the reactivity of active *N*‐centered radicals to selectively access *N*‐oxidation products. Herein, this study reports the engineering of cytochrome P450BM3 into multifunctional *N*‐oxidizing enzymes with the assistance of dual‐functional small molecules (DFSM) to selectively produce *N*‐oxygenation (i.e., *p*‐nitrosobenzene, *p*‐nitrobenzene, and azoxybenzene) and one‐electron oxidation products (i.e., oligomeric quinones and azobenzene) from aromatic amines. The best mutant, F87A/T268V/V78T/A82T, exclusively gives *p*‐nitrosobenzene (up to 98% selectivity), whereas the selectivity for *p*‐nitrobenzene is >99% using the mutant F87A/T268V/A82T/I263L. Crystal structure analysis reveals that key mutations and DFSM exert synergistic effects on catalytic promiscuity by controlling the substrate orientation in active center. This study highlights the potential of DFSM‐facilitated P450 peroxygenase and peroxidase for the synthesis of *N*‐containing compounds via the controllable oxidation of aromatic amines, substantially expanding the chemical space of P450 enzymes.

## Graphical abstract

Highly divergent *N*‐oxidation of primary arylamines is achieved by combining P450 variants and exogenous dual‐functional small molecules (DFSM) for the selective production of *p*‐nitrosobenzene, *p*‐nitrobenzene, oligomeric quinones, and azobenzenes. Crystal structures reveal a crucial synergistic effect of key mutations and DFSM in generating the catalytic promiscuity of P450 by controlling substrate orientation.

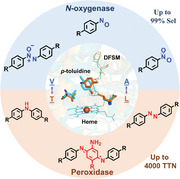

## 1 Introduction

The oxidation of primary aromatic amines is an important organic transformation to prepare a variety of high‐value *N*‐containing chemicals and synthetic intermediates. For example, the process can yield aromatic azo compounds, which are widely used in industry as dyes, pigments, food additives, photochemical switches, and therapeutic agents,^[^ ^1^, ^2^ ^]^ and nitroso, nitro, and azoxy‐compounds, which are versatile intermediates in pharmaceuticals and fine chemicals.^[^ ^3^, ^4^, ^5^, ^6^, ^7^, ^8^ ^]^ Many strategies, including transition metal catalysis, organic catalysis, photocatalysis, and heterogeneous catalysis, have been developed for the oxidation of aromatic amines.^[^ ^9^, ^10^, ^11^, ^12^, ^13^, ^14^ ^]^ However, most chemical catalytic approaches are limited by harsh reaction conditions and severe environmental issues, sometimes leading to uncontrolled overoxidation. Alternatively, natural oxidizing enzymes can catalyze the highly selective oxidation of aromatic amines under mild conditions. For instance, *N*‐oxygenases,^[^ ^15^, ^16^ ^]^ *N*‐hydroxylases,^[^ ^17^, ^18^ ^]^ and a few haloperoxidases^[^ ^19^, ^20^ ^]^ catalyze the two‐electron oxidation of aromatic amines to hydroxylamine, nitroso, and nitro compounds.^[^ ^21^, ^22^, ^23^, ^24^ ^]^ Horseradish peroxidase^[^ ^25^, ^26^ ^]^ and laccase^[^ ^27^, ^28^ ^]^ catalyze the one‐electron oxidation of aromatic amines to azo compounds or oligomers, such as dimers or trimers.

**Scheme 1:**
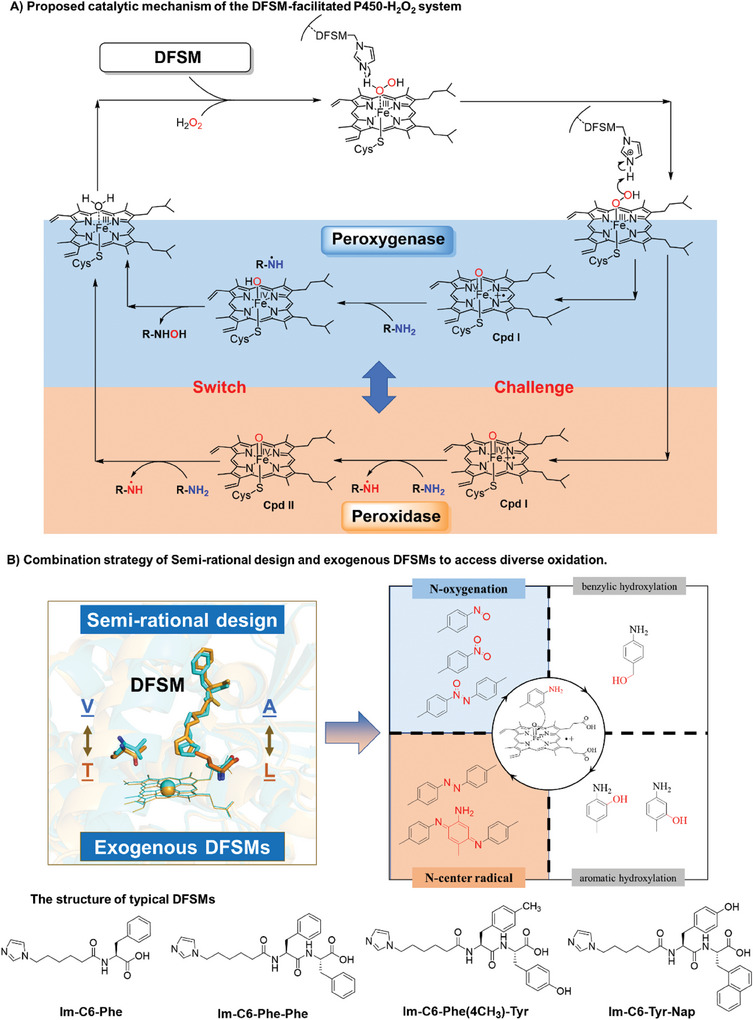
A) Proposed catalytic cycle of the DFSM‐facilitated P450BM3‐H_2_O_2_ system and the challenge to modulate peroxygenase and peroxidase mode of this system. B) Combination strategy of semi‐rational design and exogenous DFSMs to access diverse *N*‐oxidation.

Cytochrome P450 monooxygenases (P450s) can oxidize aromatic amines to produce *N*‐hydroxylamine with NAD(P)H/O_2_, while producing nitrobenzene with cumene hydroperoxide as a terminal oxidant.^[^ ^29^, ^30^, ^31^ ^]^ Accordingly, P450 can be engineered into multifunctionalized *N*‐oxidizing enzymes for aromatic amines through protein engineering. However, this is extremely challenging because aromatic amines contain multiple reactive sites. For example, the oxidation of *p*‐toluidine catalyzed by P450 forms at least four main products: benzylic hydroxylation, aromatic hydroxylation, *N*‐oxygenation, and one‐electron oxidation products.^[^ ^32^, ^33^, ^34^ ^]^ *N*‐oxygenation and one‐electron oxidation can lead to complicated product distributions. We reasoned that controlling the orientation of the arylamine substrate in the active site of P450 is necessary to access a specific product; such manipulations are usually achieved through protein engineering. Inspired by the application of a small molecule auxiliary to regulate enzyme reactivity and selectivity,^[^ ^35^, ^36^, ^37^ ^]^ we have recently used a unique dual‐functional small‐molecule (DFSM) to develop a P450‐H_2_O_2_ system and provide additional support for tuning substrate orientations. In this system, exogenous DFSM (such as *N*‐(ω‐imidazol‐1‐yl hexanoyl)‐*l*‐phenylalanine, Im‐C6‐Phe) bound to the long‐chain fatty acid hydroxylase P450BM3 acts as a general acid‐base catalyst in the H_2_O_2_‐dependent activation of P450 (**Scheme** **1**A).^[^ ^38^ ^]^ We have also demonstrated that the introduction of DFSMs could significantly affect the enantioselectivity and regioselectivity in various oxidative reactions catalyzed by P450BM3, including styrene epoxidation,^[^ ^39^ ^]^ *O*‐demethylation of aromatic ethers,^[^ ^40^ ^]^ and the diverse C‐H hydroxylation of alkylbenzenes.^[^ ^41^ ^]^

Natural P450BM3 catalyzes the *p*‐ and *o*‐hydroxylation of aniline.^[^ ^42^, ^43^ ^]^ We hypothesized that a protein engineering approach combined with the DFSM‐facilitated P450BM3 system could achieve selective *N*‐oxidation of arylamine. Herein, we report the development of multifunctional enzymes capable of *N*‐oxidation of primary aromatic amines by combining engineered P450BM3 and typical DFSMs (Scheme 1),^[^ ^44^ ^]^ efficiently generating the *N*‐oxygenation products nitrosobenzene and its derivatives nitrobenzene and azoxybenzene as well as one‐electron oxidation products, such as oligomeric quinones and azobenzenes. We also evaluated mechanisms underlying the formation of these products based on crystallographic investigations and additional experiments as well as the potential for semi‐preparative‐scale synthesis.

## 2 Results

### 2.1 Initial Screening

**Figure 1:**
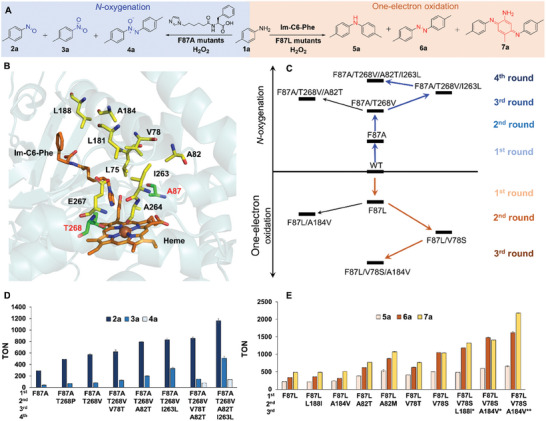
A) *N‐*oxidation of *p*‐toluidine catalyzed by the synergistic use of P450BM3 variants and Im‐C6‐Phe. B) Location of selected key sites in the active site of P450BM3 (PDB No. 7EGN^[^ ^47^ ^]^). C) Screening flowchart. D) *N*‐oxygenation and E) one‐electron oxidation of *p*‐toluidine catalyzed by synergistic use of P450BM3 variants and Im‐C6‐Phe. Conditions: P450 (0.5 µM), Im‐C6‐Phe (500 µM), *p*‐toluidine (10 mM), for *N*‐oxygenation, H_2_O_2_ (80 mM) in pH 8.0 phosphate buffer; for one‐electron oxidation, H_2_O_2_ (40 mM) in pH 7.0 phosphate buffer; * H_2_O_2_ (20 mM) in pH 7.0 phosphate buffer; ** H_2_O_2_ (20 mM) in pH 6.0 phosphate buffer.

Residue 87 significantly affects the catalytic activity and selectivity of DFSM‐facilitated P450BM3.^[^ ^38^, ^39^, ^40^, ^45^ ^]^ A single F87 mutation library of the P450BM3 heme domain was screened in phosphate buffer, pH 8.0 and 7.0, for the oxidation of *p*‐toluidine (**Figure** **1**; Figures S1, S2 and Tables S1–S3, Supporting Information). No products were detected in the absence of Im‐C6‐Phe under either pH condition. The addition of Im‐C6‐Phe greatly improved the *N*‐oxidation activity of the F87A variant to give *p*‐nitrosotoluene (**2a**) and *p*‐nitrotoluene (**3a**) with turnover numbers (TONs) of 291 and 41 in phosphate buffer (pH 8.0) (Figure 1D; Figure S1 and Table S2, Supporting Information). Notably, C–H hydroxylation and one‐electron oxidation products were not detected. Interestingly, variants of F87 with larger residues, such as F87I, F87L, F87Q, F87K, and F87N, gave di‐4‐tolylamine (**5a**) and 4,4′‐dimethylazobenzene (**6a**) as the major products in the presence of Im‐C6‐Phe in pH 7.0 phosphate buffer but without any *N*‐oxygenation product. This was in line with our previous results; it is possible that a bulky residue at F87 prevents access of the arylamine substrate to the active site and blocks the general hydroxylation pathway of “H‐abstract and OH‐rebound”.^[^ ^46^ ^]^ Among these, F87L showed the best catalytic TONs of 221 and 337 for **5a** and **6a** (Figure 1E; Figure S2 and Table S3, Supporting Information). These results indicate the crucial role of DFSM in enhancing the peroxygenase or peroxidase activity of P450BM3 variants. In addition, the catalytic preferences of F87A and F87L under different pH conditions suggest that directed evolution of P450BM3 through protein engineering can generate multifunctional *N*‐oxidizing enzymes.

### 2.2 Stepwise Protein Engineering Toward Multifunctional *N*‐Oxidizing Enzyme

Based on these results, F87A and F87L were used as parent enzymes in the next step of protein engineering to improve peroxygenase and peroxidase activity toward *N*‐oxygenation and one‐electron oxidation reactions. In addition, we demonstrated that T268 mutations significantly influenced regio‐ and enantioselectivity in the DFSM‐facilitated P450BM3 peroxygenase system. Therefore, libraries of the double mutants F87A/T268 and F87L/T268 were prepared to screen beneficial variants for *N*‐oxygenation and one‐electron oxidation products in buffer (pH 8.0 or pH 7.0) (Figure 1; Tables S4 and S5, Supporting Information). As expected, hydrophobic mutations of T268 based on F87A, such as F87A/T268 V and F87A/T268P, resulted in an improvement of >1.5‐fold in the catalytic total TON (TTN) for *N*‐oxygenation reactions over that of F87A. F87A/T268 V gave the best TON for **2a** (572) and **3a** (80) among the examined double mutations (Figure 1D; Figure S3 and Table S4, Supporting Information). However, the double mutation F87L/T268 did not improve the formation of the one‐electron oxidation products **5a** and **6a** even in pH 7.0 phosphate buffer (Table S5, Supporting Information).

Following a well‐established semi‐rational FRISM (focused rational iterative site‐specific mutagenesis) approach,^[^ ^39^, ^41^, ^45^, ^48^, ^49^, ^50^ ^]^ we then selected F87A/T268V (AV) as the parent enzyme in two‐electron oxidation for the next round of mutations. Additional mutations of other key residues (L75, V78, A82, L181, A184, L188, I263, A264, and E267) around the active site were introduced (Figure 1B, C; Table S1, Supporting Information). Each selected residue was mutated to several structurally and functionally diverse amino acids to better adapt to the substrate *p*‐toluidine, and a library of 50 mutants was prepared and screened. Various triple mutants, AV/V78T, AV/A82T, AV/A82V, AV/A184V, AV/L188I, AV/I263L, and AV/E267Q, obviously improved the catalytic TONs of *N*‐oxygenation products over that of the parent AV (Figure 1D; Figure S4 and Table S6, Supporting Information).

**Figure 2:**
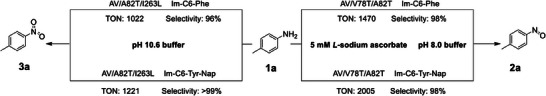
*N*‐oxygenation of *p*‐toluidine (10 mM) by typical P450BM3 mutants (0.5 µM) in the presence of Im‐C6‐Phe (0.5 mM) or Im‐C6‐Tyr‐Nap (5 µM) and H_2_O_2_ (80 mM) at 25 °C in optimized reaction conditions; for **2a** in 5 mM *L*‐sodium ascorbate pH 8.0 phosphate buffer; for **3a** in pH 10.6 phosphate buffer.

Quadruple mutants were prepared by combining beneficial triple mutants to further improve their catalytic activity. The most active quadruple mutant AV/A82T/I263L gave catalytic TONs of 1164 and 507 for **2a** and **3a**, which were almost 4‐fold and 12‐fold higher than that of the initial mutant F87A. In addition, the by‐product 4,4′‐dimethylazoxybenzene (**4a**), which might form from cross‐coupling of **2a** and the undetected monooxygenation product *N*‐(*p*‐tolyl) hydroxylamine,^[^ ^51^, ^52^ ^]^ was identified in the reactions catalyzed by the quadruple mutants (Figure 1D; Figure S5 and Table S9, Supporting Information). Despite the relatively low *N*‐oxygenation activity, another quadruple mutant, AV/V78T/A82T, showed better chemoselectivity (≈80%) for **2a** (TONs = 857, 143, and 78 for products **2a**, **3a**, and **4a**) than that of AV/A82T/I263L (≈64%) (Figure 1D; Figure S5 and Table S9, Supporting Information). The chemoselectivities of **2a** and **3a** were dramatically affected by the pH condition and ascorbate (Figure S5 and Tables S7, S8, S10, and S11, Supporting Information). AV/V78T/A82T gave **2a** in a catalytic TON of 1470 (98% selectivity) in the presence of 5 mM *L*‐sodium ascorbate in phosphate buffer (pH 8.0) (**Figure** **2**, Figures S5 and S6, Supporting Information). The control experiments with **2a** as a substrate indicated that ascorbate played a role of reductant in preventing further oxidation of **2a** to **3a** (Figure S7, Supporting Information). Similarly, AV/A82T/I263L gave **2a** in a catalytic TON of 2001 (97% selectivity) under the same conditions (Figure S5 and Table S10, Supporting Information); however, it converted *p*‐toluidine to **3a** with a selectivity of 96% (TON = 1022) in carbonate buffer at pH 10.6 (Figure 2; Figures S5, S8 and Table S11, Supporting Information). Recently, we have developed the second‐generation DFSMs (Im‐DFSM‐dipeps) by equipping a peptide module as anchoring group. This significantly enhanced the binding affinity of DFSMs and decreased their stoichiometric load to as low as 2 equivalents of P450, together with maintained or increased activity for expoxidation and hydroxylation reactions.^[^ ^44^ ^]^ We therefore examined the effect of Im‐DFSM‐dipeps for the formation of *p*‐nitrosotoluene and *p*‐nitrotoluene, respectively (Scheme 1). The combination of AV/V78T/A82T with Im‐C6‐Tyr‐Nap (10 equiv.) achieved a higher TON of 2005 (an improvement to 1.4‐fold over the best Im‐C6‐Phe combination) for *p*‐nitrosotoluene wi maintaining 98% selectivity. The combination of AV/A82T/I263L with Im‐C6‐Tyr‐Nap (10 equiv.) boosted the selectivity of *p*‐nitrotoluene to over 99% and slightly improved catalytic activity (Figure 2; Figure S9 and Tables S12–S14, Supporting Information). To our knowledge, this is the first example of exclusively converting *p*‐toluidine to *p*‐nitrosotoluene and *p*‐nitrotoluene by engineered P450 mutants. Notably, their *N*‐oxygenation activities exceeded those of most oxidizing enzymes reported to date, except for chloroperoxidase from *Caldariomyces fumago*.^[^ ^53^ ^]^

Because the introduction of T268 mutations to F87L did not improve the catalytic TONs for the one‐electron oxidation of *p*‐toluidine, we prepared a series of F87L‐based double mutants by incorporating the key residues located around the active site following the same FRISM approach (Figure 1B,C; Table S1, Supporting Information). Several double mutants, including F87L/A82T, F87L/A82M, F87L/A184V, F87L/L188I, F87L/V78T, and F87L/V78S, showed improved TONs toward the one‐electron oxidation products **5a**, **6a** and *N*, *N*‐di‐*p*‐tolyl‐5‐amino‐2‐methyl‐2,5‐cyclohexadiene‐1,4‐diimine (**7a**). The TONs of the best double mutant, F87L/V78S, were 501, 1047, and 1041 for products **5a**, **6a** and **7a**, which were more than 2.4‐fold, 2.7‐fold, and four‐fold higher than those of F87L, respectively (Figure 1E; Table S15, Supporting Information). The combination of beneficial double mutants further improved the catalytic activity for one‐electron oxidation (Table S18, Supporting Information). Among them, F87L/V78S/A184V showed the highest TTN of 3479 for all one‐electron oxidation products (**5a**: **6a**: **7a** = 17:42:41). In addition, when the reaction was carried out in phosphate buffer at pH 6.0, the TON of **5a**, **6a**, and **7a** further increased to 660, 1622, and 2177, which were 1.1‐fold, 1.1‐fold, and 1.5‐fold improvements over those of the reaction at pH 7.0, respectively (Figure 1E; Figure S10 and Table S20, Supporting Information). Notably, a >99% utilization efficiency of H_2_O_2_ was achieved when the concentration of H_2_O_2_ was less than 5 mM (Table S16, Supporting Information). Interestingly, we observed the difference of Im‐C6‐dipepes on one‐electron oxidation reactions from *N*‐oxygenations. The catalytic performance of the mutant F87L decreased for one electron oxidation of *p*‐toluidine when using Im‐C6‐dipepes instead of Im‐C6‐Phe. This may be ascribed to the introduction of additional redox‐sensitive residues, such as tyrosine or phenylalanine on Im‐C6‐dipepes. Redox sensitive residues are considered potential competitors for single electron oxidation substrates.^[^ ^50^, ^54^, ^55^ ^]^

### 2.3 Substrate Scope

Encouraged by these excellent results, we examined the oxidation of *p*‐substituted anilines with electron‐withdrawing groups, such as halogen and trifluoromethyl, using beneficial mutants evolved for one‐electron oxidation or *N*‐oxygenation reactions (**Table** **1**; Tables S21–S47, Supporting Information). Among them, the oxidation of *p*‐bromoaniline (**1d**) catalyzed by AV/A82T/A184V afforded the better catalytic activity for *N*‐oxygenation products. The reaction yielded *p*‐bromonitrosobenzene (**2d**) as the major product (99% selectivity) with a TON of 4220 together with small amounts of *p*‐bromonitrobenzene (**3d**) in the presence of 5 mM *L*‐sodium ascorbate in phosphate buffer at pH 8.0 (Table 1; Table S38, Supporting Information). The reaction yielded *p*‐bromonitrobenzene (**3d**) as the sole product (>99% selectivity) with a TON of 1845 in carbonate buffer (pH 10.6) (Table 1; Figures S11, S12, and Table S39, Supporting Information). Although AV/A82T/A184V showed comparable activity to that of **1d** for the nitrosation of *p*‐chloroaniline (**1c**), the nitration activity of **1c** was obviously decreased, even for the best mutant, AV/A82T/I263L (Tables S31 and S32, Supporting Information). Furthermore, the nitrosation and nitration activities of the electron‐withdrawing substituents *p*‐fluoroaniline (**1b**) and *p*‐trifluoromethylaniline (**1e**) were much lower than those of **1d** and **1c** by almost the same P450 mutant. These results suggest that the electronic properties of the arylamine substrates affect *N*‐oxygenation activity to a certain extent. In fact, the presence of electron‐withdrawing groups had a more significant impact on the one‐electron oxidation activity of the arylamine substrates. The TTN of the one‐electron oxidation products of all of the examined substrates **1b**–**1e** decreased significantly (Table 1). For instance, *p*‐fluoroaniline showed the highest one‐electron oxidation activity among the substrates **1b‐1e** to give two products, 4,4′‐difluoroazobenzene (**5b**) and a trimer **6b** with a TTN of 1150 (Table 1; Figure S13, Tables S26, and S27, Supporting Information), which was only one‐fourth that of the one‐electron product of *p*‐toluidine (**1a**). For *p*‐trifluoromethylaniline (**1e**), only traces of the one‐electron oxidation products were detected (Table 1; Table S47, Supporting Information).

**Table 1:** Substrate scopes of *N*‐oxidation of *p*‐substituted anilines catalyzed by the DFSM‐facilitated P450BM3‐H_2_O_2_ system.

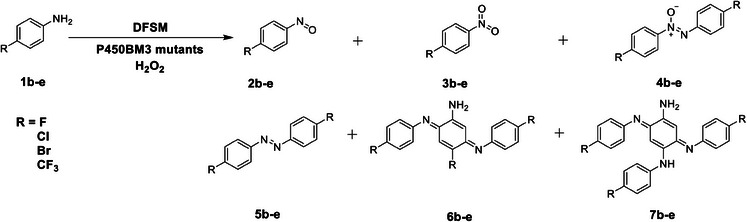								
Substrate	R	P450 variants	TON					
			2b‐e	3b‐e	4b‐e	5b‐e	6b‐e	7b‐e
1b	F	AV/A82T/I263L^a^ ^)^	2279 ± 17					
		AV/A82T/I263L^b^ ^)^		1129 ± 1				
		F87L/V78S/A184V^c^ ^)^				373 ± 11	777 ± 5	
1c	Cl	AV/A82T/A184V^d^ ^)^	4274 ± 13	30 ± 1	77 ± 1			
		AV/A82T/I263L^b^ ^)^		1583 ± 4				
		F87L/V78S/A184V^c^ ^)^				198 ± 3	193 ± 24	154 ± 19
1d	Br	AV/A82T/A184V^d^ ^)^	4220 ± 102	47 ± 1				
		AV/A82T/A184V^b^ ^)^		1845 ± 153				
		F87L/V78S/A184V^c^ ^)^				113 ± 1	143 ± 1	174 ± 1
1e	CF_3_	AV/A82T/A184V^d^ ^)^	2195 ± 29	78 ± 2				
		AV/A82T/A184V^e^ ^)^		1041 ± 1	15 ± 1			
		F87L^c^ ^)^				trace		

^a)^ Reaction conditions: heme domain of P450BM3 variants (0.5 µM), substrate (10 mM, dissolved in 2% DMSO), *L*‐sodium ascorbate (5 mM), H_2_O_2_ (80 mM), Im‐C6‐Phe‐Phe (5 µM) in 0.1 M phosphate buffer (pH 8.0);

^b)^ Reaction conditions: heme domain of P450BM3 variants (0.5 µM), substrate (10 mM, dissolved in 2% DMSO), H_2_O_2_ (80 mM), Im‐C6‐Tyr‐Nap (5 µM) in 0.1 M carbonate buffer (pH 10.6);

^c)^ Reaction conditions: heme domain of P450BM3 variants (0.5 µM), substrate (10 mM, dissolved in 2% DMSO), H_2_O_2_ (40 mM), Im‐C6‐Phe (0.5 mM) in 0.1 M phosphate buffer (pH 7.0);

^d)^ Reaction conditions: same to [a], except using Im‐C6‐Tyr‐Nap (5 µM) instead of Im‐C6‐Phe‐Phe (5 µM);

^e)^ Reaction conditions: same to [b], except using Im‐C6‐Phe (0.5 mM) instead of Im‐C6‐Tyr‐Nap (5 µM).

### 2.4 Structural Insights into Diverse *N*‐Oxidizing Pathways

**Figure 3:**
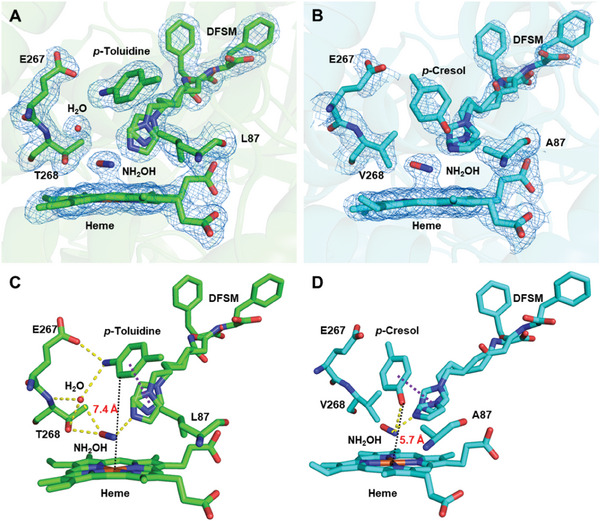
Active site of P450BM3 mutants in complex with the DFSM (Im‐C6‐Phe), NH_2_OH, and *p*‐toluidine/*p*‐cresol. Ligands and substrate‐binding residues of F87L/V78S/A184V (PDB ID: 7Y0R) and AV/A82T/I263L (PDB ID: 7Y0P) are represented as stick models in green and cyan, respectively. The water molecules are drawn as sphere models in red. The hydrogen bonds, *π*–*π* interactions, and distance between the substrate and heme are shown as dashed lines in yellow, purple, and black, respectively. The 2Fo‐Fc electron‐density maps contoured at 1.0σ are shown in the blue mesh. A) The 2Fo‐Fc electron‐density map of F87L/V78S/A184V active site; B) The 2Fo‐Fc electron‐density map of F87A/T268V/A82T/I263L active site; C) Hydrogen bonds and *π*–*π* interactions of *p*‐toluidine binding site in F87L/V78S/A184V; D) Hydrogen bonds and *π*–*π* interactions of *p*‐cresol binding site in F87A/T268V/A82T/I263L.

To explore the structural basis of diverse *N*‐oxidizing pathways and to understand the catalytic mechanism, two superior mutants, F87L/V78S/A184V and AV/A82T/I263L, were crystallized in the presence of the DFSM molecule Im‐C6‐Phe, the substrate *p*‐toluidine, and the H_2_O_2_ analog NH_2_OH. The crystal structure of F87L/V78S/A184V in complex with the three components was determined at 2.09 Å and the electron density corresponding to all ligands is of sufficient quality for analyzing the catalytic mode in the active site (**Figure** **3**A; Table S50, Supporting Information). DFSM adopted two conformations, as previously reported.^[^ ^41^, ^46^ ^]^ Furthermore, similar to the previously solved crystal structure of F87L in complex with phenol (PDB entry: 7WDG),^[^ ^46^ ^]^ the bulky side chain of L87 blocked the approach of substrate **1a** toward the heme center. Consequently, two‐electron *N*‐oxygenation cannot occur readily as a result of the long distance between the substrate and heme, leading only to one‐electron *N*‐oxidation reactions. Meanwhile, the amine group of *p*‐toluidine interacted with E267 and T268 through hydrogen‐bonding networks, and the aromatic ring of **1a** had *π*–*π* interactions with the imidazolyl moiety of Im‐C6‐Phe, which was consistent with the phenol bond crystal structure 7WDG (Figure 3C). The co‐crystal structures (PDB ID: 7Y0R, 7Y0P) clearly indicated the crucial role of the large steric hindrance at position 87 in controlling the direction of the reaction pathway between one‐electron oxidation and *N*‐oxygenation. This was further supported by a crystallographic study of AV/A82T/I263L and its substrates. The crystal structure of AV/A82T/I263L in complex with **1a** (2.31 Å) revealed that *p*‐toluidine directly coordinated to the heme iron, suggesting that the smaller residue A87 enabled the substrate to approach the heme center (Figure S14A, Supporting Information). This also suggests that arylamine substrates may cause P450 enzyme inactivation because nitrogen‐containing heterocyclic or amino compounds can easily coordinate to heme iron, such as nicotine and some amino acids^[^ ^56^, ^57^ ^]^ (Figure S14B,C, Supporting Information). However, the inactivation of P450 caused by *N*‐Fe coordination did not occur in the current reaction system, according to the observed high *N*‐oxygenation activity of AV/A82T/I263L. We speculate that crystallization was a slow reaction process, whereas the *N*‐oxygenation reaction was completed instantaneously. Some factors in the reaction system may delay the approach of the substrate arylamine to the heme iron. The crystal structure of AV/A82T/I263L in complex with Im‐C6‐Phe, NH_2_OH, and *p*‐cresol (an analog of *p*‐toluidine) was determined at 1.99 Å, providing a possible structural explanation (Figure 3B). The hydroxyl group of *p*‐cresol is not directly coordinated to the heme iron. Instead, it faced toward heme and formed a hydrogen bond with NH_2_OH, suggesting a suitable distance between the terminal OH (NH_2_ in the case of *p*‐toluidine) and the heme iron, facilitating *N*‐oxygenation. More importantly, the aromatic ring of *p*‐cresol also interacted with Im‐C6‐Phe through *π*–*π* interactions, as mentioned above (Figure 3D). We believe that the *π*–*π* interaction enabled the completion of the *N*‐oxygenation reaction before the *N*‐Fe coordination occurs. In addition, the polar interactions in the active site of AV/A82T/I263L were weaker than those in F87L/V78S/A184V in terms of the number of hydrogen bonds, resulting in weaker binding of *p*‐toluidine. This could be ascribed to the T268V mutation, which not only changed the local hydrophilic environment around residues 267–268 but also created the amine moiety of *p‐*toluidine toward heme for *N*‐oxygenation.

### 2.5 Verification of the Catalytic Mechanism

**Figure 4:**
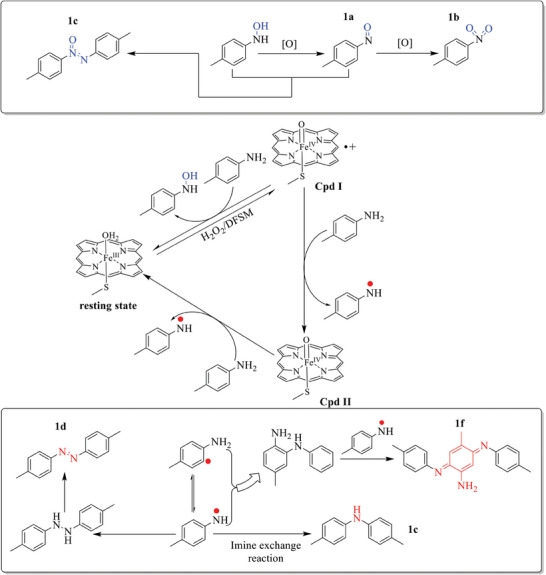
Possible catalytic mechanism of *N*‐oxygenation and one‐electron oxidation and radical trapping mechanism of TEMPO.

To further investigate the catalytic mechanisms of the different *N*‐oxidizing pathways, we added a free radical scavenger, TEMPO (2,2,6,6‐tetramethyl‐1‐piperidinyloxy), to the reaction system (**Figure** **4**). No one‐electron oxidation products (**5a**, **6a**, or **7a**) were detected; only the starting **1a** was recovered when 2 mM TEMPO was added to the oxidation reaction of *p*‐toluidine **1a** catalyzed by F87L/V78S/A184V in the presence of Im‐C6‐Phe (Table S48, Supporting Information). This result strongly supported the idea that the F87L/V78S/A184V‐catalyzed reaction adopts an *N*‐centered radical mechanism (Figure 4). In contrast, for the oxidation of *p*‐toluidine catalyzed by AV/A82T/I263L in the presence of Im‐C6‐Phe, the addition of TEMPO did not inhibit and even slightly improved both the product formation rate (PFR) and the TTNs of *N*‐oxygenation products. The PFR and TTN increased 2‐fold and 1.1‐fold, respectively, compared with those in the TEMPO‐free system (Table S49, Supporting Information). This result further indicated that the fast sequential process of “H‐abstract” and “OH‐rebound” occurred in *p*‐toluidine oxidation by AV/A82T/I263L (Figure 4). In addition, the presence of the free radical scavenger TEMPO may play a role in trapping unfavorable reactive oxygen species, thus improving the stability of the DFSM‐facilitated P450 peroxygenase system.

### 2.6 Scalable Preparations

To further examine its practical applicability, we investigated the semi‐preparative‐scale synthesis of *p*‐nitrosotoluene, *p*‐bromonitrobenzene, and one‐electron oxidation products, including 4,4′‐dimethylazobenzene and *N*, *N*‐di‐*p*‐tolyl‐5‐amino‐2‐methyl‐2,5‐cyclohexadiene‐1, 4‐diimine (trimer), using the DFSM‐facilitated P450BM3 peroxizyme system under standard conditions (**Table** **2**). The oxidation of *p*‐toluidine (2 mM) catalyzed by AV/A82V/L188I (2 µM) gave *p*‐nitrosotoluene with an isolated yield of 71% as a colorless liquid (17.2 mg), corresponding to 87% *p*‐toluidine conversion (Figure S15, Supporting Information). The same mutant AV/A82T/A184V (6 µM) catalyzed the conversion of *p*‐bromoaniline (4 mM) to deliver *p*‐bromonitrobenzene in 77% isolated yield (30.4 mg) (Figure S16, Supporting Information). One‐electron oxidation of *p*‐toluidine by the mutant F87L/V78S/A184 V gave 4,4′‐dimethylazobenzene (13 mg, 31%, yellow solid) and trimer product (9.9 mg, 24%, red solid) with 78% conversion (Figure S17, Supporting Information). These results indicated the potential of the DFSM‐facilitated P450 peroxizyme system for preparing divergent *N*‐oxidation products from aromatic amines.

**Table 2:** Semi‐preparative scale synthesis of divergent *N*‐oxidation products catalyzed by the typical DFSM‐facilitated P450BM3‐H_2_O_2_ system.

Substrate	Product	Mutant	Conversion^a^ ^)^/%	Yield^b^ ^)^/%
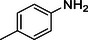	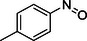	AV/A82V/L188I^c^ ^)^	87	71
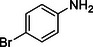	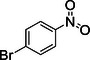	AV/A82T/A184V^d^ ^)^	91	77
	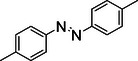			31
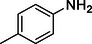		F87L/V78S/A184V^e^ ^)^	78	
	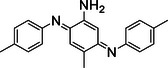			24

^a)^ Conversion ratio (%) = converted substrate/initial substrate × 100;

^b)^ Isolated yields based on the initial substrate;

^c)^ Reaction conditions: AV/A82V/L188I (2 µM), *p*‐toluidine (2 mM, dissolved in 2% DMSO), *L*‐sodium ascorbate (5 mM), H_2_O_2_ (80 mM, after 5 minutes add 30 mM), Im‐C6‐Phe‐Phe (5 µM) in 0.1 M phosphate buffer (pH 8.0);

^d)^ Reaction conditions: F87A/T268V/A82T/A184 V (6 µM), *p*‐bromoaniline (4 mM, dissolved in 2% DMSO), H_2_O_2_ (100 mM), Im‐C6‐Phe‐Phe (5 µM) in 0.1 M carbonate buffer (pH 10.6);

^e)^ F87L/V78S/A184 V (0.5 µM), *p*‐toluidine (8 mM, dissolved in 2% DMSO), H_2_O_2_ (40 mM), Im‐C6‐Phe (0.5 mM) in 0.1 M phosphate buffer (pH 7.0).

## 3 Conclusion

In summary, we successfully engineered P450BM3 into multifunctional *N*‐oxidizing enzymes for primary aromatic amines in the presence of exogenous DFSM molecules. Crystallographic studies clearly showed the synergistic effect of mutations of key residue and DFSM molecules in controlling *N*‐oxidation pathways, leading to divergent *N*‐oxygenation and one‐electron oxidation products. Furthermore, two *N*‐oxygenation products, nitrosobenzene and nitrobenzene, and one‐electron oxidation products, such as azobenzene, were readily prepared using the current P450 peroxizyme system on a milligram scale. This study provides a versatile toolbox of non‐natural *N*‐oxidizing enzymes to access miscellaneous *N*‐containing aromatic chemicals. This provides a basis for manipulating the reactivity and chemoselectivity of *N*‐centered radicals using engineered P450 peroxizymes, which substantially expands the chemical space of P450 enzymes. It is also to note that it is still a challenge to selectively access either one‐electron oxidation products for the current system. Considering the unique benefits of the DFSM‐facilitated P450BM3‐H_2_O_2_ system, further investigations aimed at improving reaction selectivity (e.g., azobenzene compounds) and substrate promiscuity are now underway in our laboratory.

## 4 Experimental Section

### General Procedure for *p‐*Substituted Aniline Oxidation

P450BM3 heme domain mutant (0.5 µM) was transferred to a glass sample bottle containing 0.1 M, pH 7.0, 8.0 or 10.6 buffer, *p*‐substituted aniline (10 mM, dissolved in DMSO (2% vol/vol)) and Im‐C6‐Phe (500 µM, dissolved in pH 8.0 or 7.0 phosphate buffer) or Im‐DFSM‐dipeps (5 µM, dissolved in pH 8.0 or 7.0 phosphate buffer). The reaction was initiated by the addition of H_2_O_2_ (20/40/60/80 mM, dissolved in pH 7.0, 8.0 or 10.6 buffer). The reaction mixture was incubated in water bath at 25 °C for 30 min. After the reaction, the mixture was quenched and extracted with 1 mL of ethyl acetate, and the organic phase was separated and dried with sodium sulphate anhydrous. The product was analyzed by gas chromatography (GC) by benzophenone as an internal standard. Control reactions performed by repeating these steps in the absence of DFSMs.

### Creation of P450BM3 Variants

All mutations were made by polymerase chain reaction (PCR)‐based site‐directed mutagenesis, and the PCR‐amplified target DNA fragments were assembled by kinase‐ligase. The target mutant assembly products were transformed into chemically competent *Escherichia coli* strain BL21(DE3) cells. Single colonies from the transformation plates were selected, cultured overnight at 37 °C with kanamycin (50 µg mL^−1^), and further sequenced to confirm the mutations.

### Crystallization of the P450BM3 Mutants in Complex with the DFSM and Substrates

The affinity column purified proteins were subjected to gel filtration using a HiPrep 16/60 Sephacryl S‐200 column (Cytiva), followed by concentration and storage in 10 mM Tris buffer (pH 8.0) prior to crystallization. The crystallization experiment was performed at 18 °C using hanging drop evaporation combined with seeding as described previously.^[^ ^46^ ^]^ Briefly, multi‐crystals of F87A mutant in complex with Im‐C6‐Phe were prepared by mixing 1 mL protein solution (30 mg mL^−1^ protein supplemented with 1 mM Im‐C6‐Phe) and 1 mL reservoir solution A (0.1 M Tris pH 8.0, 0.38 M MgCl_2_ and 28% PEG 3350). The multi‐crystals were crushed into microcrystals and diluted 1000‐fold with reservoir solution A as the seeding solution. The rod‐like single crystals of F87L/V78S/A184V and F87A/T268V/A82T/I263L mutants in complex with Im‐C6‐Phe were obtained within 1 week by mixing 1.5 mL protein solution, 1 mL reservoir solution B (0.1 M Tris pH 8.5, 0.38 M MgCl_2_, and 10–20% PEG 3350), and 0.5 mL seeding solution. The crystals were soaked in a cryo‐protecting solution containing 0.1 M Tris pH 8.5, 0.38 M MgCl_2_, 16% PEG 3350, 1 mM Im‐C6‐Phe, 20% glycerol, 0.5 M hydroxylamine, 5–100 mM *p*‐toluidine or 100 mM p‐cresol for 3 min before transferring into the liquid nitrogen for data collection.

The X‐ray diffraction data were collected at BL02U1 and BL10U2 beamlines of the Shanghai Synchrotron Radiation Facility (SSRF) with a DECTRIS EIGER X 16 M detector at 100 K and processed with Aquarium.^[^ ^58^ ^]^ The structure was solved by molecular replacement with PHENIX Phaser‐MR,^[^ ^59^ ^]^ using the previously determined structure of P450BM3 heme domain (PDB ID: 7EGN) as the search model. The structure model was further built using COOT^[^ ^60^ ^]^ and refined by PHENIX Refine.^[^ ^61^ ^]^ The overall quality of the structural model was checked by MolProbity,^[^ ^62^ ^]^ and the structure has been deposited in the Protein Data Bank (PDB ID: 7Y0P, 7Y0Q&7Y0R). Data collection and refinement statistics are shown in Table S35 (Supporting Information). Protein structure graphics were generated by PyMOL (https://www.schrodinger.com).

## Conflict of Interest

The authors declare no conflict of interest.

## Author Contributions

J.C., F.Y., and Y.J. contributed equally to this work. Z.C. conceived the project. Z.C., Y.F., and M.X. supervised the investigation. Z.C., J.C., F.Y., and Y.J. designed the experiments. J.C., F.Y., and Y.J. performed the experiments and data analysis. X.Q. assisted in the experiments or data analysis. Z.C., J.C., F.Y., and Y.J. wrote the manuscript upon discussion and comments from all authors.

## Supporting information



Supporting Information

## Data Availability

The data that support the findings of this study are available from the corresponding author upon reasonable request.
