# Supplementary material for: Diverse N‐Oxidation of Primary Aromatic Amines Controlled by Engineered P450 Peroxizyme Variants Facilitated by Dual‐Functional Small Molecule
Source: Adv Sci (Weinh). 2024 Dec 16;12(6):2412100. doi: 10.1002/advs.202412100 (PMC11809401; doi:10.1002/advs.202412100)
Supplement: Supplementary file 1 — Supporting Information [file ADVS-12-2412100-s001.pdf]

ADVANCED SCIENCE
Open Access

Supporting Information

for *Adv. Sci.*, DOI 10.1002/advs.202412100

Diverse *N*-Oxidation of Primary Aromatic Amines Controlled by Engineered P450 Peroxizyme Variants Facilitated by Dual-Functional Small Molecule

*Jie Chen, Fuquan Yao, Yiping Jiang, Xiangquan Qin, Mo Xian, Yingang Feng and Zhiqi Cong**

Supporting Information

Diverse *N*-oxidation of Primary Aromatic Amines Controlled by Engineered P450 Peroxizyme Variants Facilitated by Dual-functional Small Molecule

Jie Chen,‡[a, b] Fuquan Yao,‡[a] Yiping Jiang,‡[a] Xiangquan Qin,[a] Mo Xian,[a, b] Yingang Feng,[a, b] and Zhiqi Cong*[a, b]

[a] Key Laboratory of Photoelectric Conversion and Utilization of Solar Energy, Qingdao New Energy Shandong Laboratory, CAS Key Laboratory of Biofuels, Shandong Provincial Key Laboratory of Synthetic Biology, Qingdao Institute of Bioenergy and Bioprocess Technology, Chinese Academy of Sciences, Qingdao 266101, China
[b] University of Chinese Academy of Sciences
Beijing 100049, China
‡These authors contributed equally to this work

* To whom correspondence should be addressed. E-mail: congzq@qibebt.ac.cn

This file includes:

Supporting Figures. S1-S38, Supporting Tables S1-S50, Supporting Methods, Supporting References

Supporting Figures

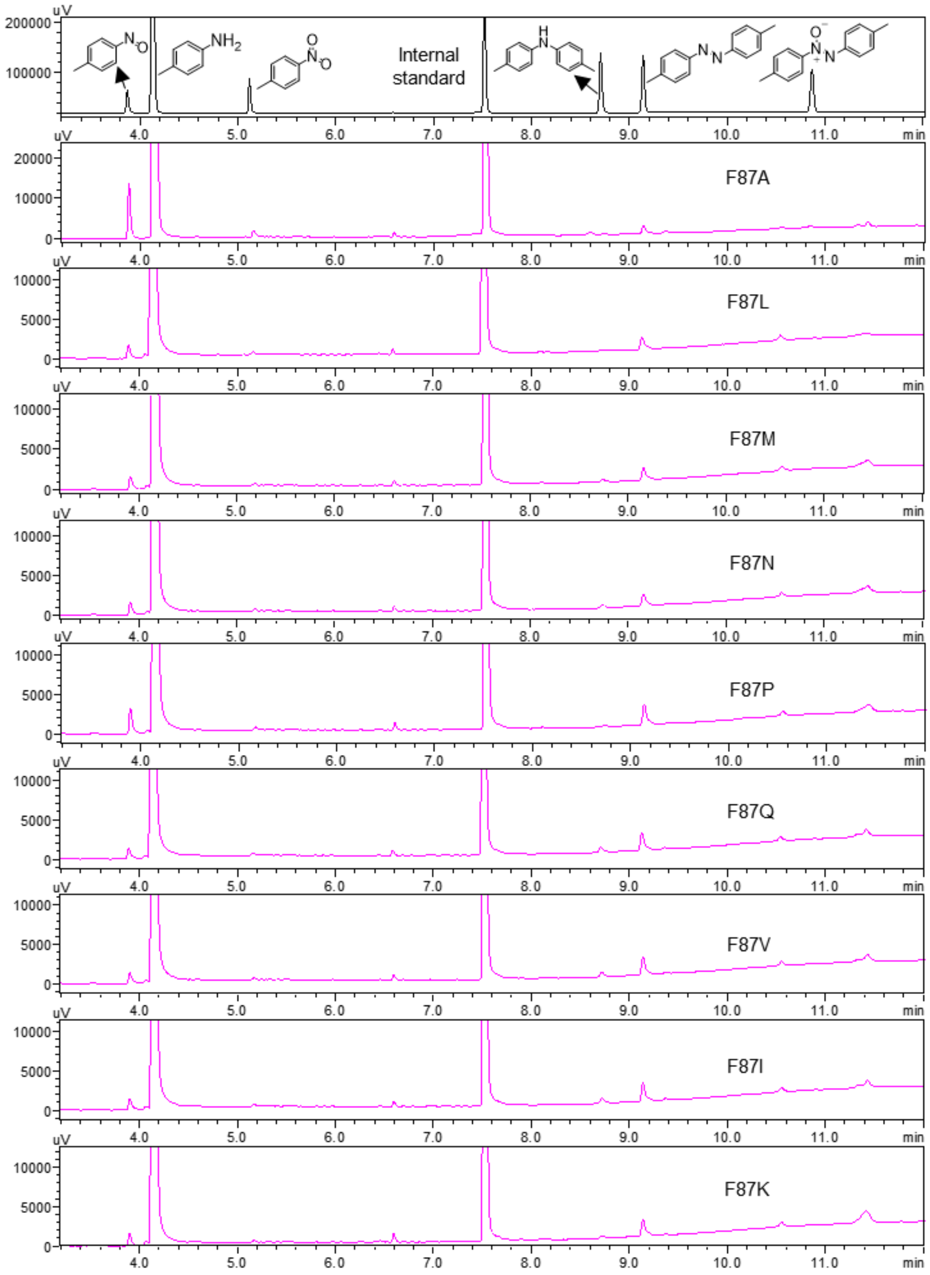

Figure S1. Typical GC analyses of the product distributions for *p*-toluidine oxidation catalyzed by F87 mutants of P450BM3 heme domain at 25 ºC in the presence of Im-C6-Phe in pH 8.0 buffer for 30 min.

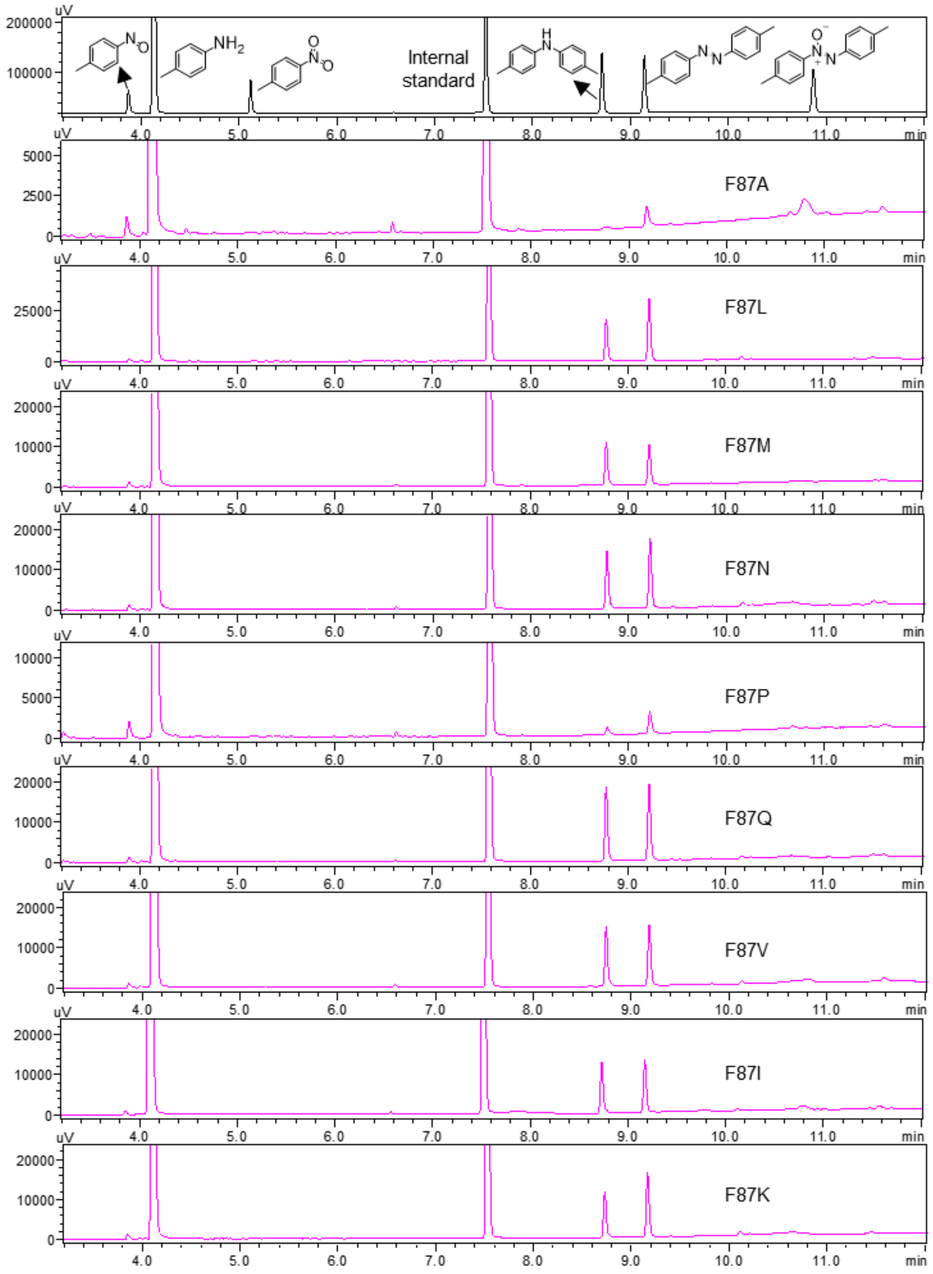

Figure S2. Typical GC analyses of the product distributions for *p*-toluidine oxidation catalyzed by F87 mutants of P450BM3 heme domain at 25 ºC in the presence of Im-C6-Phe in pH 7.0 buffer for 30 min.

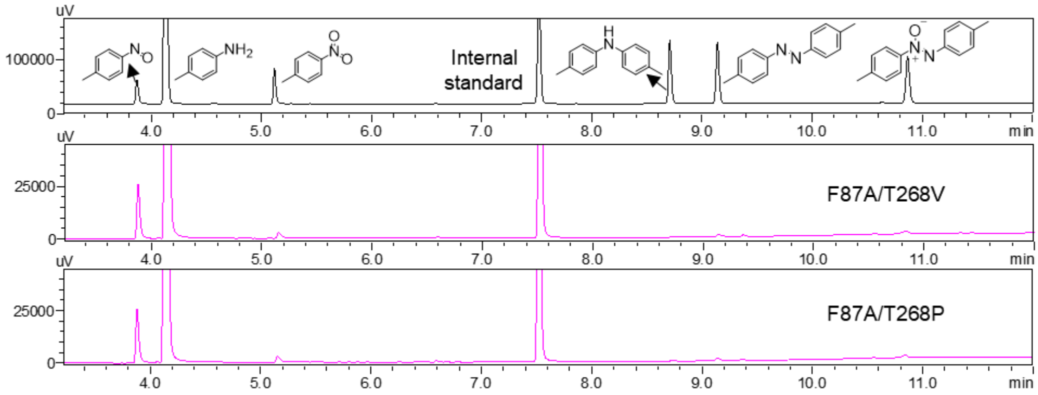

Figure S3. Typical GC analyses of the product distributions for *p*-toluidine oxidation catalyzed by F87A/T268 double mutants of P450BM3 heme domain at 25 ºC in the presence of 80 mM H_2O_2 and Im-C6-Phe in pH 8.0 buffer for 30 min.

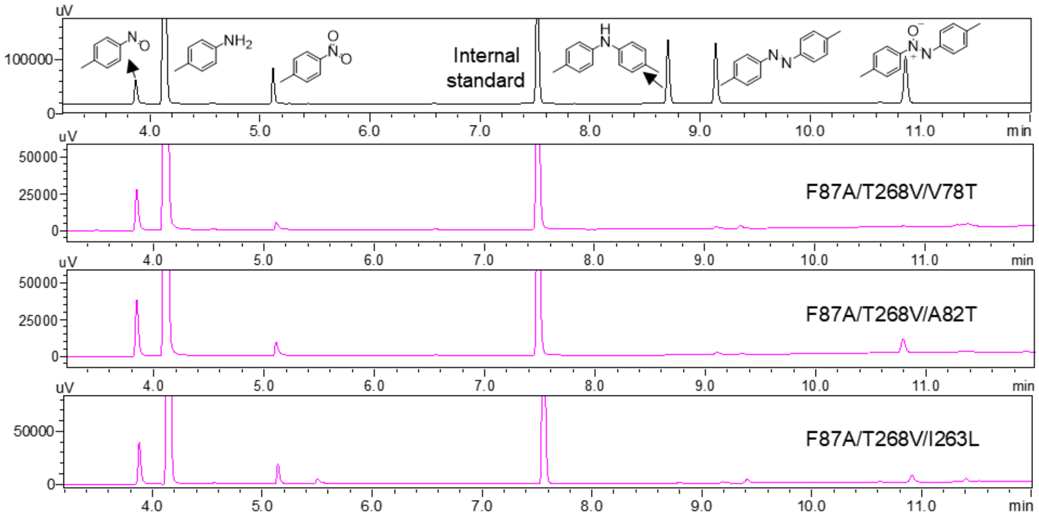

Figure S4. Typical GC analyses of the product distributions for *p*-toluidine oxidation catalyzed by triple mutants of P450BM3 heme domain at 25 ºC in the presence of 80 mM H_2O_2 and Im-C6-Phe in pH 8.0 buffer for 30 min.

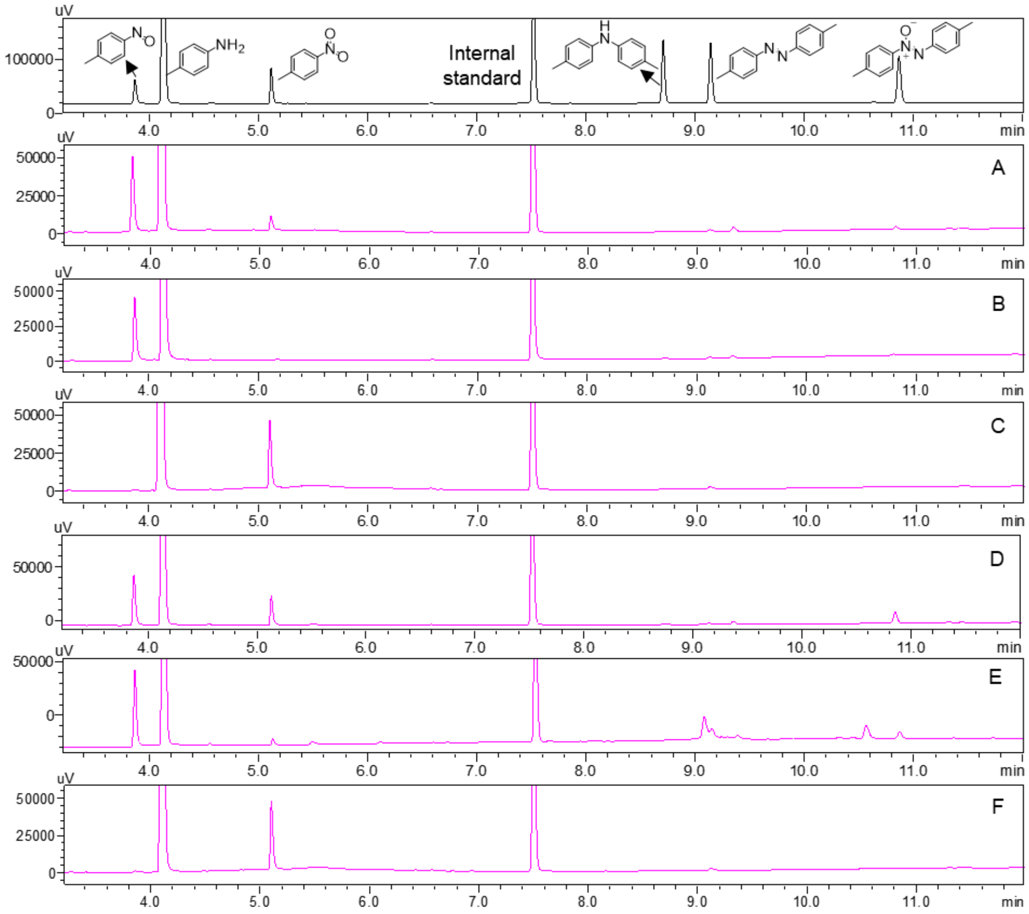

Figure S5. Typical GC analyses of the product distributions for *p*-toluidine oxidation in different reaction conditions. A) F87A/T268V/V78T/A82T of P450BM3 heme domain at 25 ºC in the presence of 80 mM H_2O_2 and Im-C6-Phe in pH 8.0 buffer for 30 min. B) F87A/T268V/V78T/A82T of P450BM3 heme domain at 25 ºC in the presence of 80 mM H_2O_2, Im-C6-Phe and 5mM *L*-sodium ascorbate in pH 8.0 buffer for 30 min. C) F87A/T268V/V78T/A82T of P450BM3 heme domain at 25 ºC in the presence of 80 mM H_2O_2 and Im-C6-Phe in pH 10.6 buffer for 30 min. D) F87A/T268V/A82T/I263L of P450BM3 heme domain at 25 ºC in the presence of 80 mM H_2O_2 and Im-C6-Phe in pH 8.0 buffer for 30 min. E) F87A/T268V/A82T/I263L of P450BM3 heme domain at 25 ºC in the presence of 80 mM H_2O_2, Im-C6-Phe and 5mM *L*-sodium ascorbate in pH 8.0 buffer for 30 min. F) F87A/T268V/A82T/I263L of P450BM3 heme domain at 25 ºC in the presence of 80 mM H_2O_2 and Im-C6-Phe in pH 10.6 buffer for 30 min.

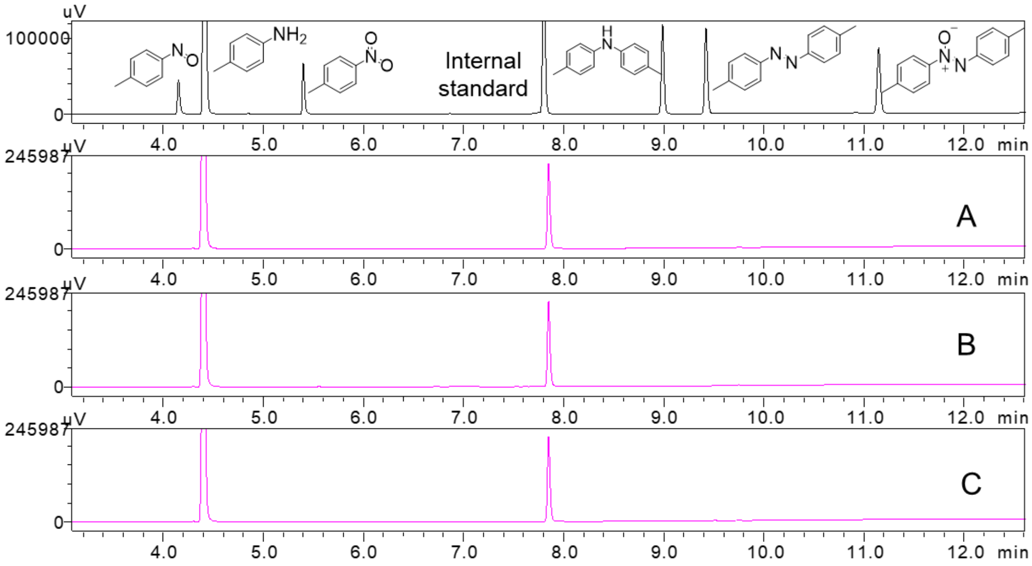

Figure S6. Typical GC analysis of F87A/T268V/V78T/A82T catalyzed control reaction of toluidine oxidation products in 5mM *L*-sodium ascorbate pH 8.0 buffer for 30 min. A) No enzyme added in the reaction system. B) No DFSM added in the reaction system. C) No H_2O_2 added in the reaction system.

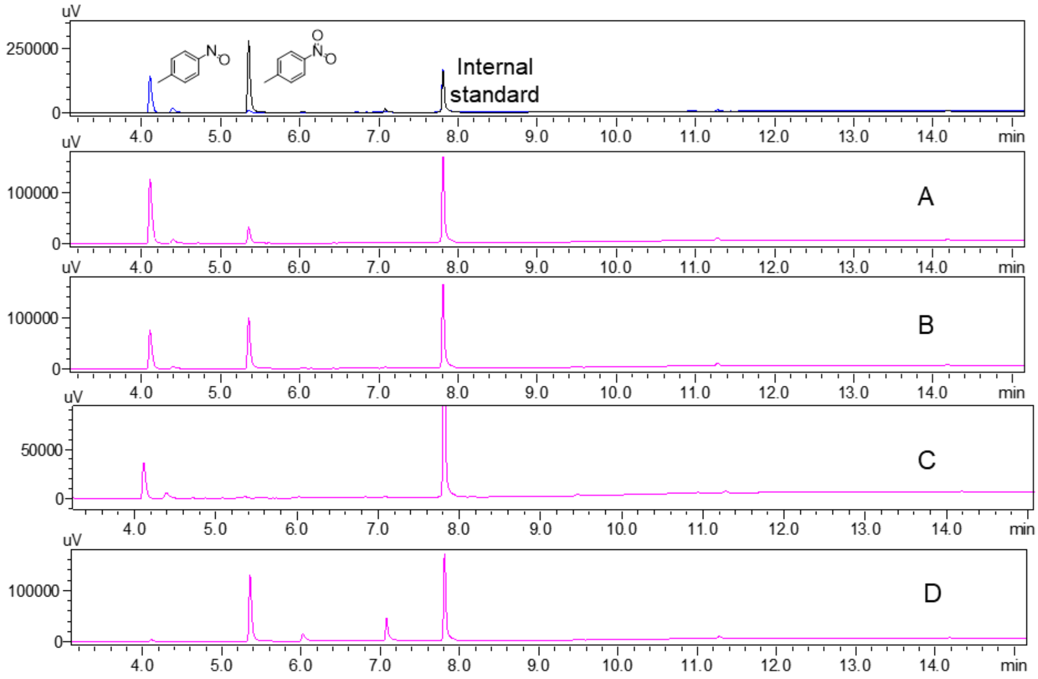

Figure S7. The Typical GC analyses of the product distributions for *p*-nitrosotoluene oxidation in different reaction conditions. A) No Enzyme at 25 ºC in the presence of 80 mM H_2O_2 and Im-C6-Phe in pH 8.0 buffer for 30min. B) F87A/T268V/V78T/A82T of P450BM3 heme domain at 25 ºC in the presence of 80 mM H_2O_2, Im-C6-Phe and in pH 8.0 buffer for 30min. C) F87A/T268V/V78T/A82T of P450BM3 heme domain at 25 ºC in the presence of 80 mM H_2O_2, Im-C6-Phe and in 5 mM *L*-sodium ascorbate pH 8.0 buffer for 30 min. D) F87A/T268V/A82T/I263L of P450BM3 heme domain at 25 ºC in the presence of 80 mM H_2O_2, Im-C6-Phe in pH 8.0 buffer for 30min.

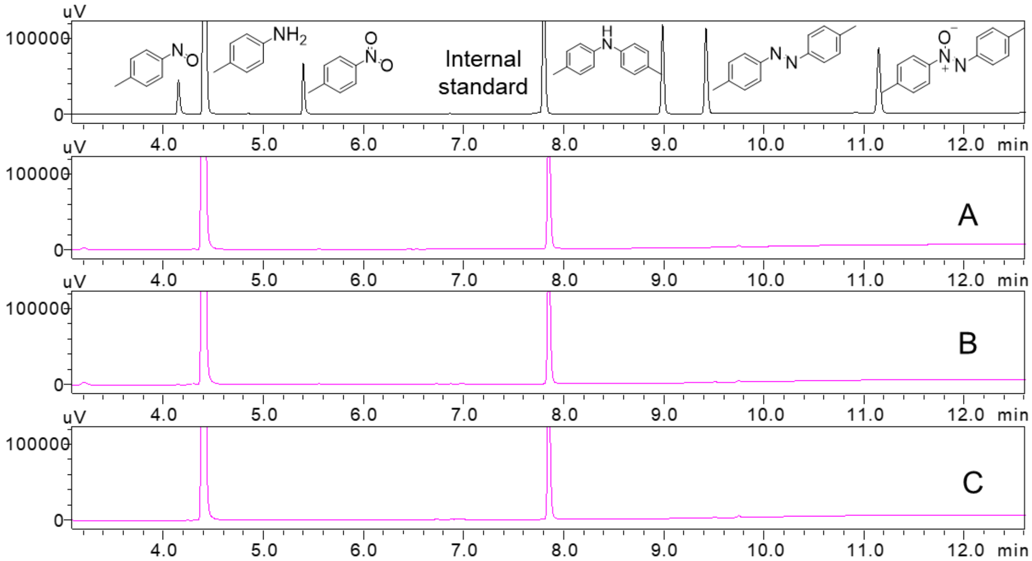

Figure S8. Typical GC analysis of F87A/T268V/A82T/I263L catalyzed control reaction of toluidine oxidation products in pH 10.6 buffer. A) No enzyme added in the reaction system for 30 min. B) No DFSM added in the reaction system for 30 min. C) No H_2O_2 added in the reaction system for 30 min.

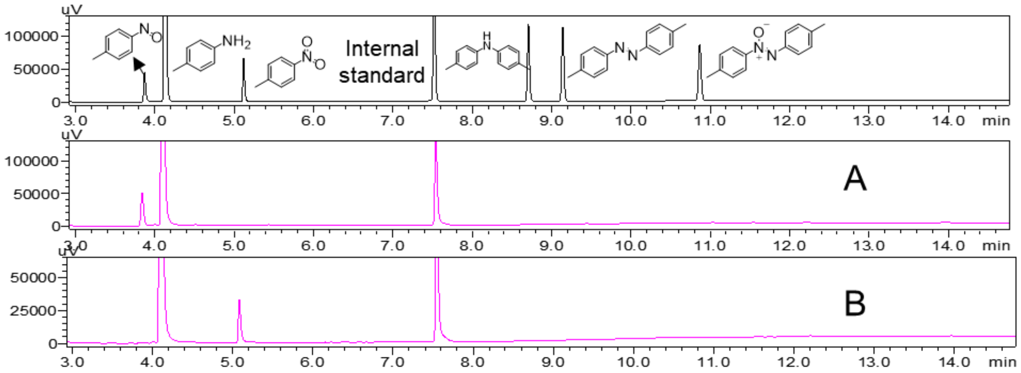

Figure S9. Typical GC analyses of the product distributions for *p*-toluidine oxidation in different reaction conditions. A) F87A/T268V/V78T/A82T of P450BM3 heme domain at 25 ºC in the presence of 80 mM H_2O_2 and Im-C6-Tyr-Nap in pH 8.0 buffer for 30 min. B) F87A/T268V/A82T/I263L of P450BM3 heme domain at 25 ºC in the presence of 80 mM H_2O_2 and Im-C6-Tyr-Nap in pH 10.6 buffer for 30 min.

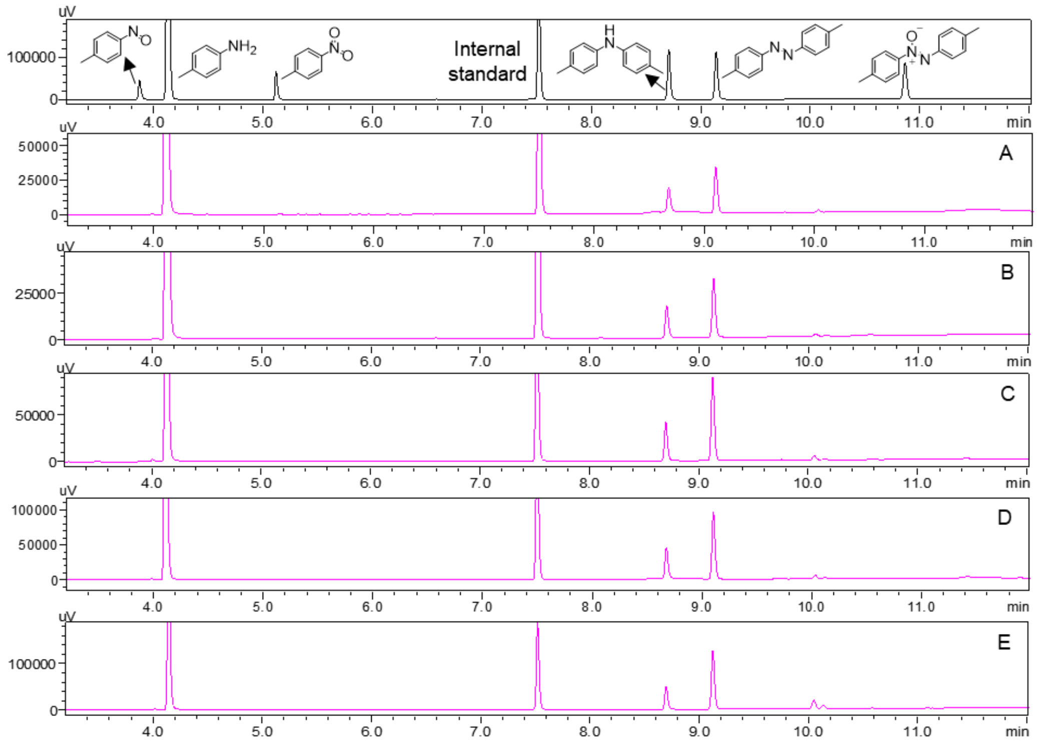

Figure S10. Typical GC analyses of the product distributions for *p*-toluidine oxidation. A) F87L of P450BM3 heme domain at 25 ºC in the presence of Im-C6-Phe and 40 mM H_2O_2 in pH 7.0 buffer for 30 min. B) F87L/A184V of P450BM3 heme domain at 25 ºC in the presence of Im-C6-Phe and 40 mM H_2O_2 in pH 7.0 buffer for 30 min. C) F87L/V78S of P450BM3 heme domain at 25 ºC in the presence of Im-C6-Phe and 40 mM H_2O_2 in pH 7.0 buffer for 30 min. D) F87L/V78S/A184V of P450BM3 heme domain at 25 ºC in the presence of Im-C6-Phe and 20 mM H_2O_2 in pH 7.0 for 30 min. E) F87L/V78S/A184V of P450BM3 heme domain at 25 ºC in the presence of Im-C6-Phe and 20 mM H_2O_2 in pH 6.0 buffer for 30 min.

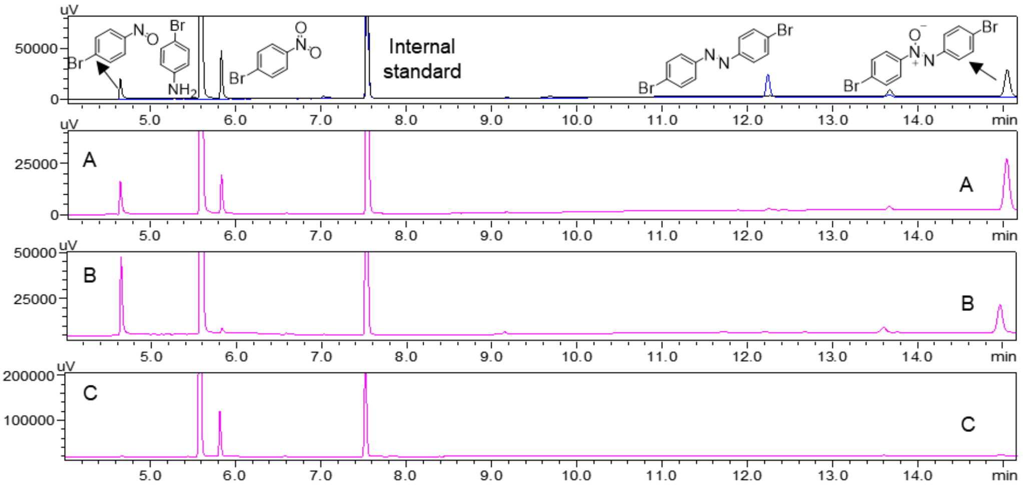

Figure S11. Typical GC analyses of the product distributions for *p*-bromoaniline oxidation in different reaction conditions. A) F87A/T268V/A82T/A184V of P450BM3 heme domain at 25 ºC in the presence of 80 mM H_2O_2 and Im-C6-Phe in pH 8.0 buffer for 30 min. B) F87A/T268V/A82T/A184V of P450BM3 heme domain at 25 ºC in the presence of 80 mM H_2O_2, Im-C6-Phe and 5mM *L*-sodium ascorbate in pH 8.0 buffer for 30 min. C) F87A/T268V/A82T/A184V of P450BM3 heme domain at 25 ºC in the presence of 80 mM H_2O_2 and Im-C6-Phe in pH 10.6 buffer for 30 min.

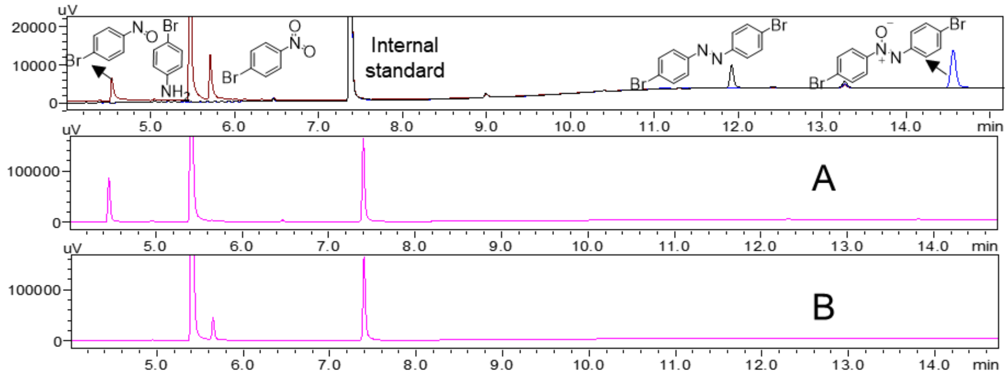

Figure S12. Typical GC analyses of the product distributions for *p*-bromoaniline oxidation in different reaction conditions. A) F87A/T268V/A82T/A184V of P450BM3 heme domain at 25 ºC in the presence of 80 mM H_2O_2 and Im-C6-Tyr-Nap in pH 8.0 buffer for 30 min. B) F87A/T268V/A82T/A184V of P450BM3 heme domain at 25 ºC in the presence of 80 mM H_2O_2 and Im-C6-Tyr-Nap in pH 10.6 buffer for 30 min.

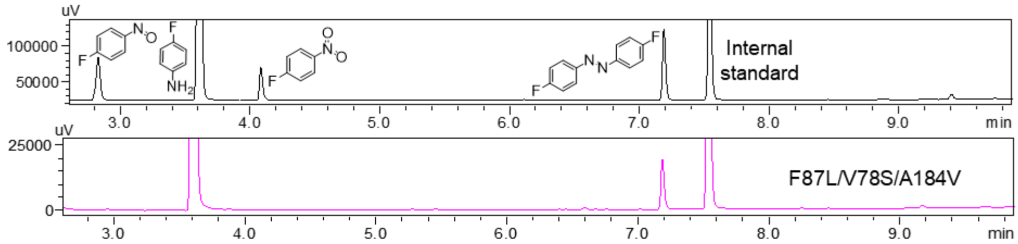

Figure S13. Typical GC analyses of the product distributions for *p*-fluoroaniline oxidation catalyzed by F87L/V78S/A184V of P450BM3 heme domain at 25 ºC in the presence of Im-C6-Phe and 40 mM H_2O_2 in pH 7.0 buffer for 30 min.

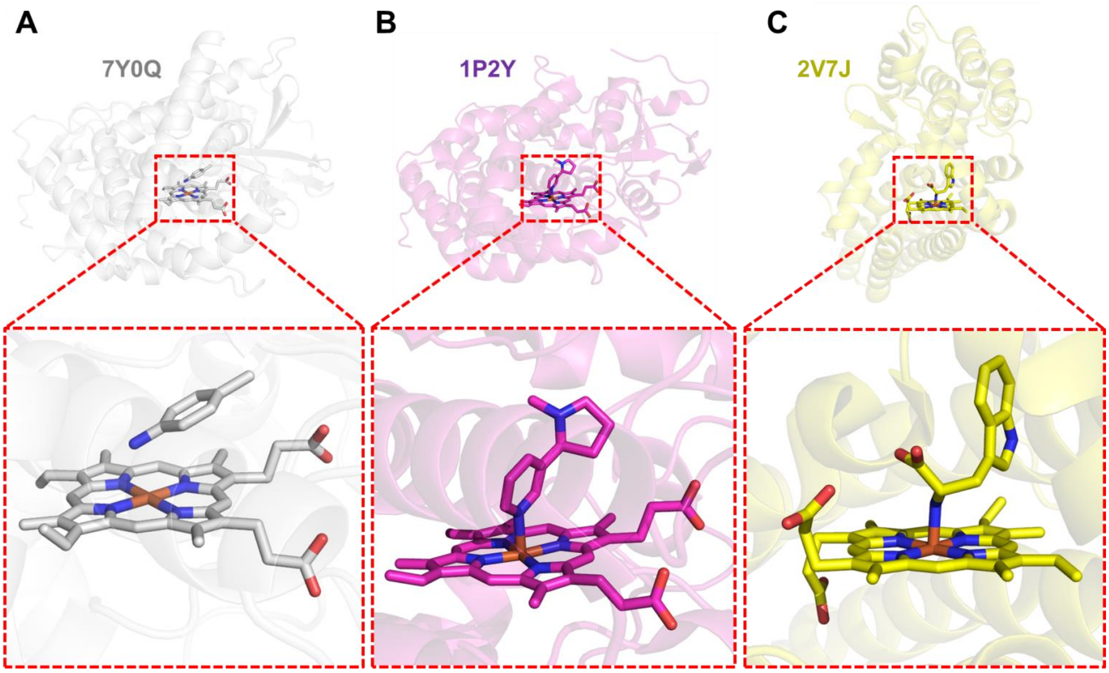

Figure S14. Crystal structures of (A) *p*-toluidine, (B) nicotine, and (C) L-tryptophan coordinated heme containing enzymes (PDB Entry: 7Y0Q, 1P2Y, 2V7J).

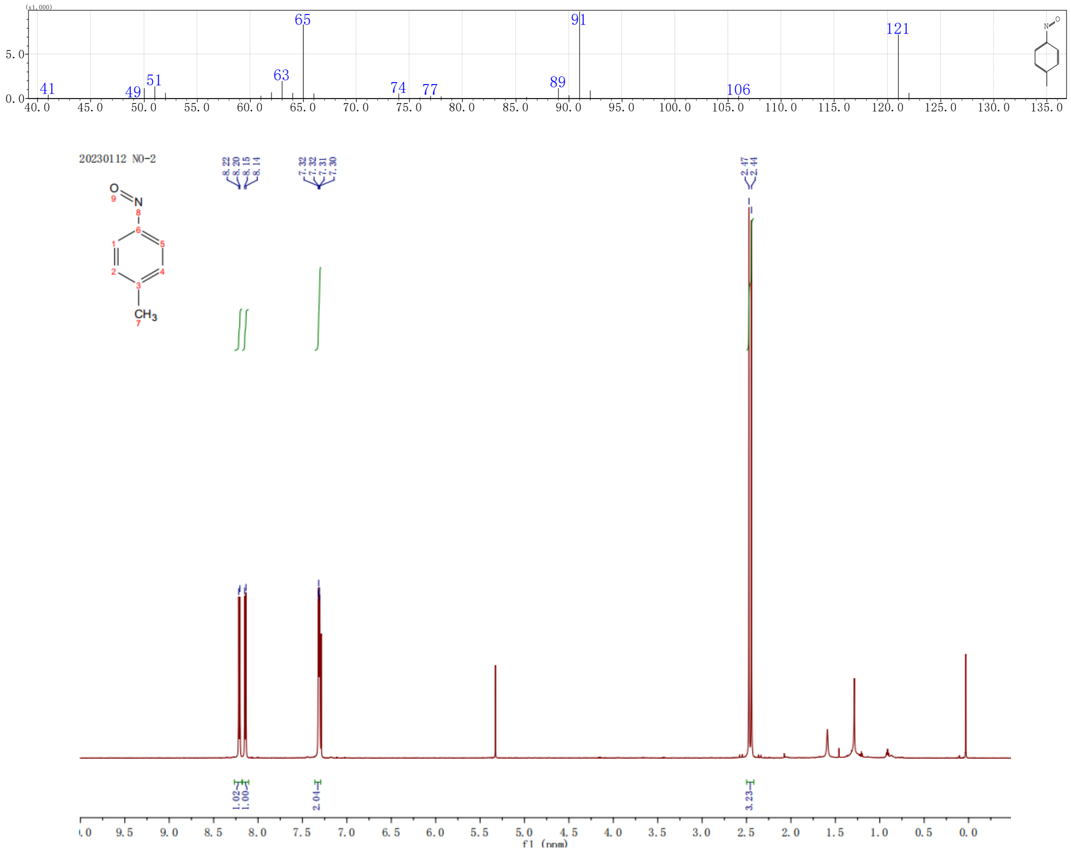

Figure S15. The spectra of EI-MS and ^{1}H NMR for the product identification of *p*-toluidine. ^{1}H NMR (600 MHz, CDCl$_3$) δ = 8.22~8.14(m, 2H), 8.15~8.14 (m, 2H), 2.47~2.44 (d, 3H). 4-Nitrosotoluene (C_7H_7NO)

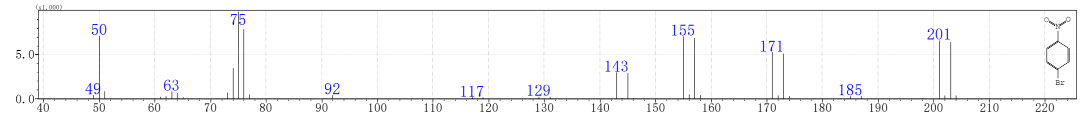

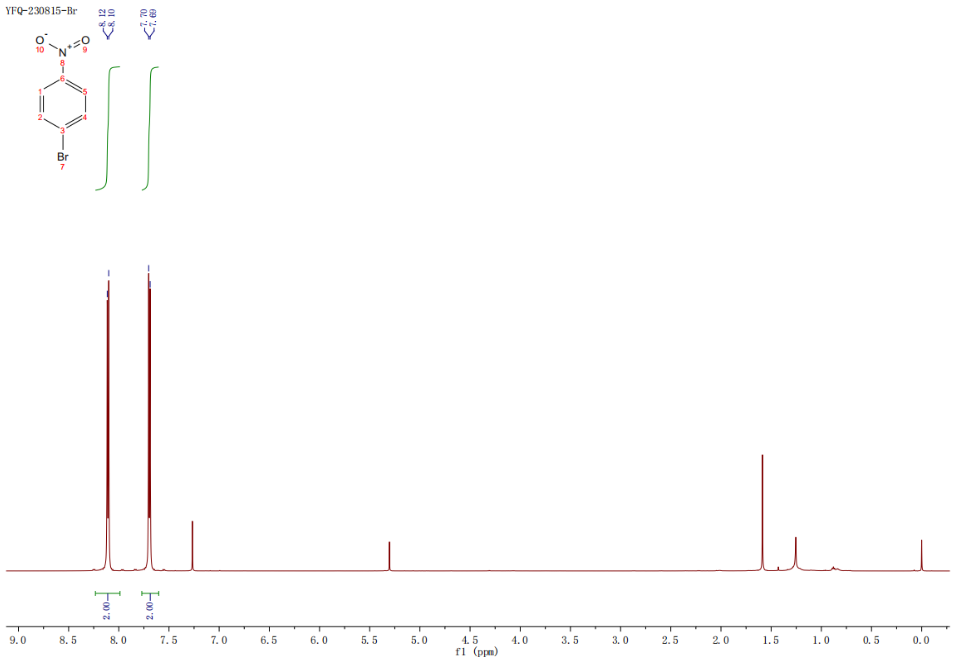

Figure S16. The spectra of EI-MS and ^{1}H NMR for the product identification of *p*-bromoaniline. ^{1}H NMR (600 MHz， $CDCl_3$) δ = 8.12~8.10 (d, 2H), 7.70~7.69 (d, 2H): *p*-nitrobromobenzene ($C_6H_4BrNO_2$).

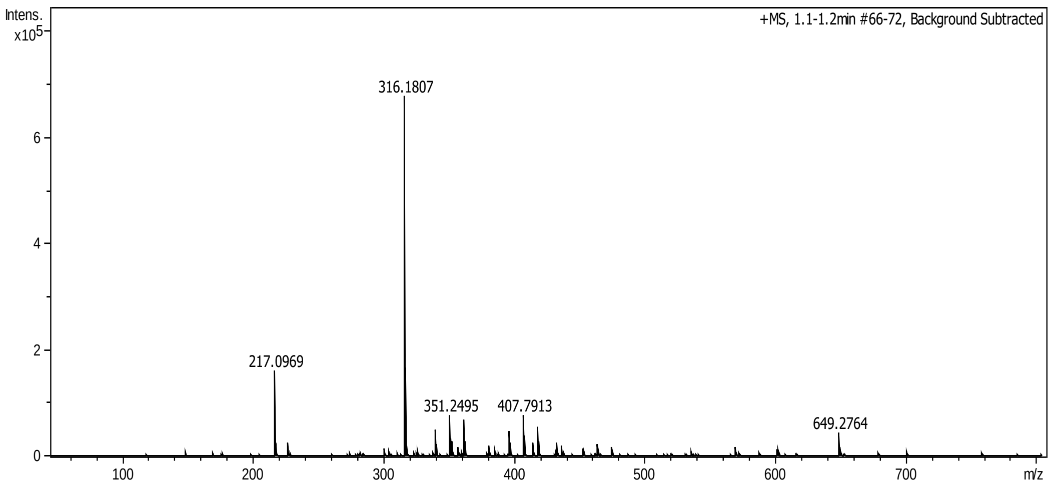

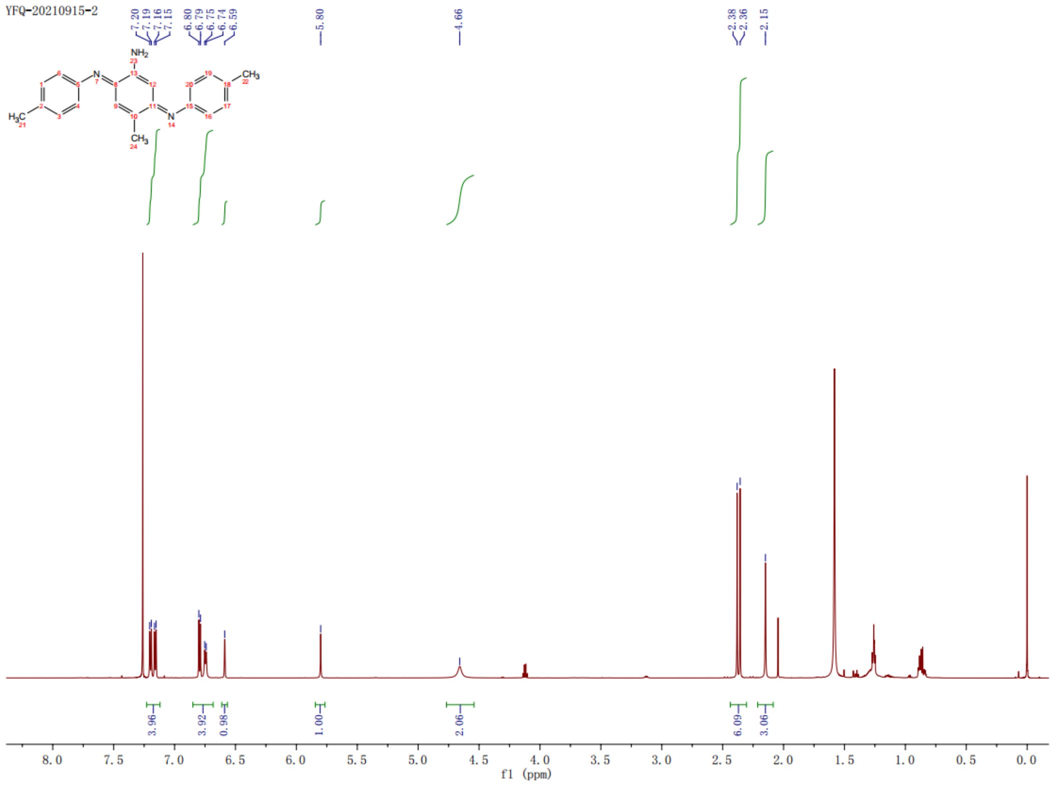

Figure S17. The spectra of ESI-MS (top) and ^{1}H NMR (bottom) for the product identification of *p*-toluidine. ^{1}H NMR (600 MHz, $CDCl_3$) δ = 7.20~7.15 (m, 4H), 6.80~6.74 (m, 4H), 6.59 (s, 1H), 5.80 (s, 1H), 6.59 (s, 1H), 4.66 (d, 2H), 2.38~2.36 (d, 6H, J = 12 Hz), 2.15 (s, 3H). MS (ESI): m/z $[M+H]^+$: N,N'-Di-p-tolyl-5-amino-2-methyl-2,5-cyclohexadiene-1,4-diimine ($C_{21}H_{21}N_3$), Calculated: 315.42 Da; Observed: 316.1807 Da ($[M+H]^+$).

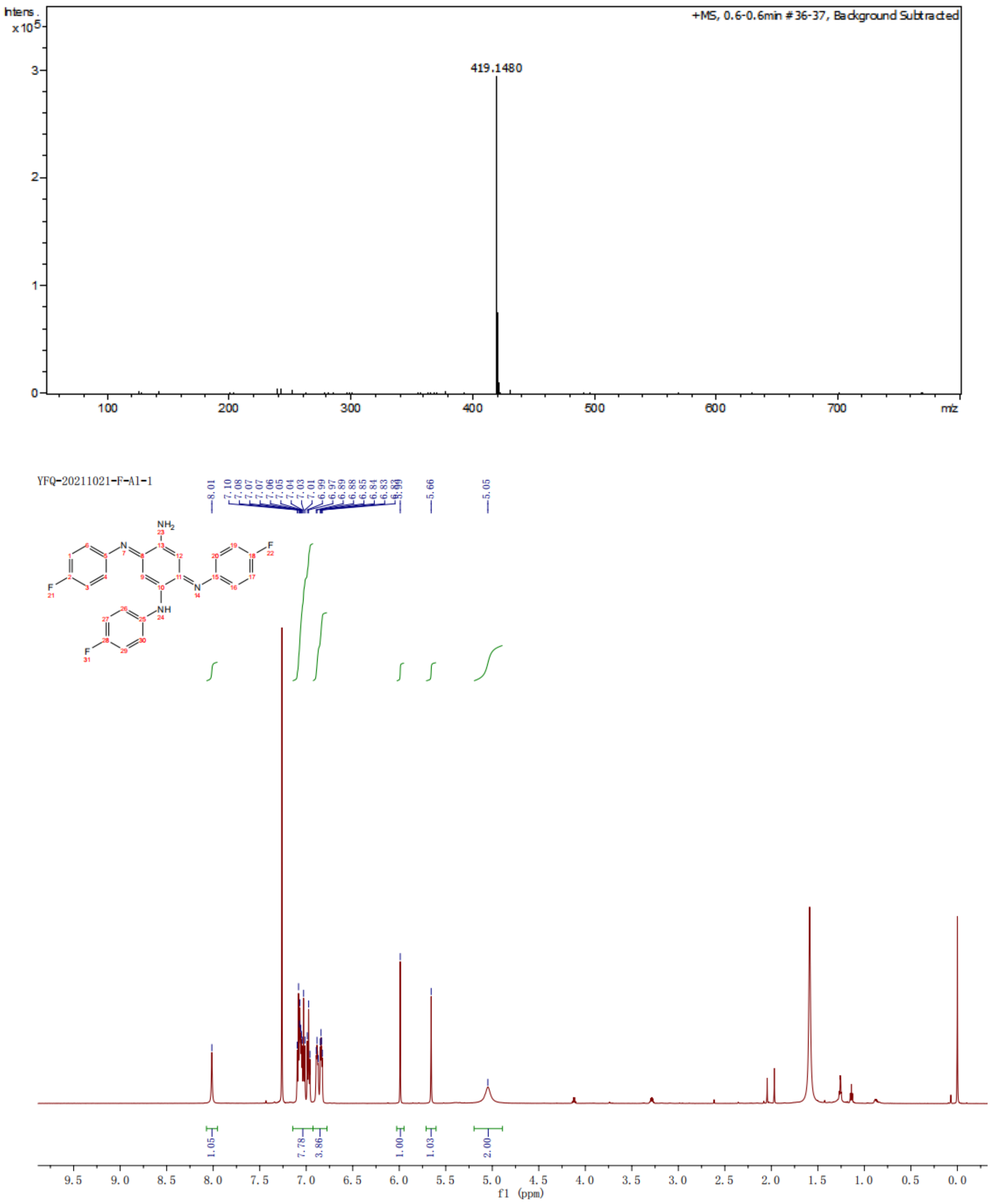

Figure S18. The spectra of ESI-MS (top) and ^{1}H NMR (bottom) for the product identification of *p*-fluoroaniline. ^{1}H NMR (600 MHz, $CDCl_3$) δ = 8.01 (s, 1H), 7.10~6.96 (m, 8H), 6.89~6.83 (m, 4H), 5.99 (s, 1H), 5.66 (s, 1H), 5.05 (s, 2H), MS (ESI): m/z $[M+H]^+$: N1-(4-Fluorophenyl)-3,6-bis[(4-fluorophenyl)imino]-1,4-cyclohexadiene-1,4-diamine ($C_{18}H_{12}F_3N_3$), Calculated: 418.42 Da; Observed: 419.1408 Da ($[M+H]^+$).

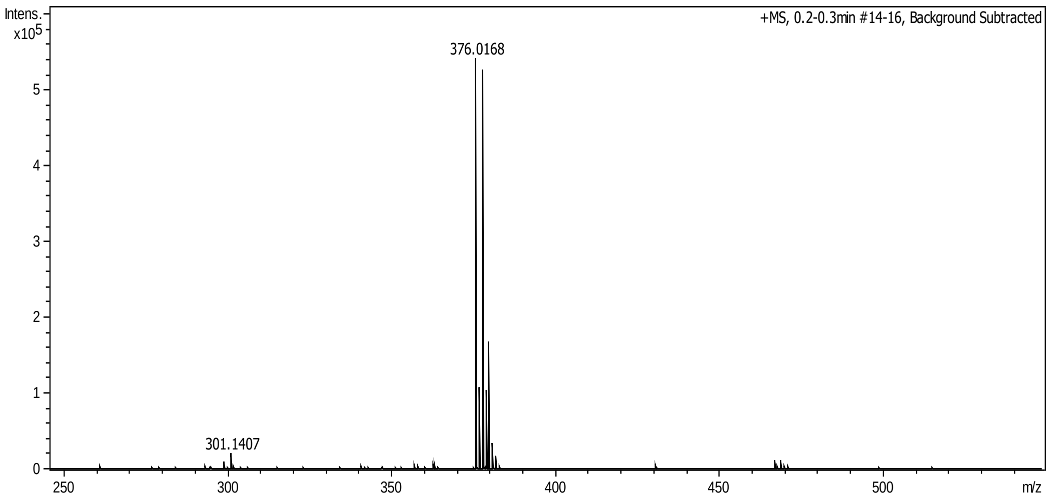

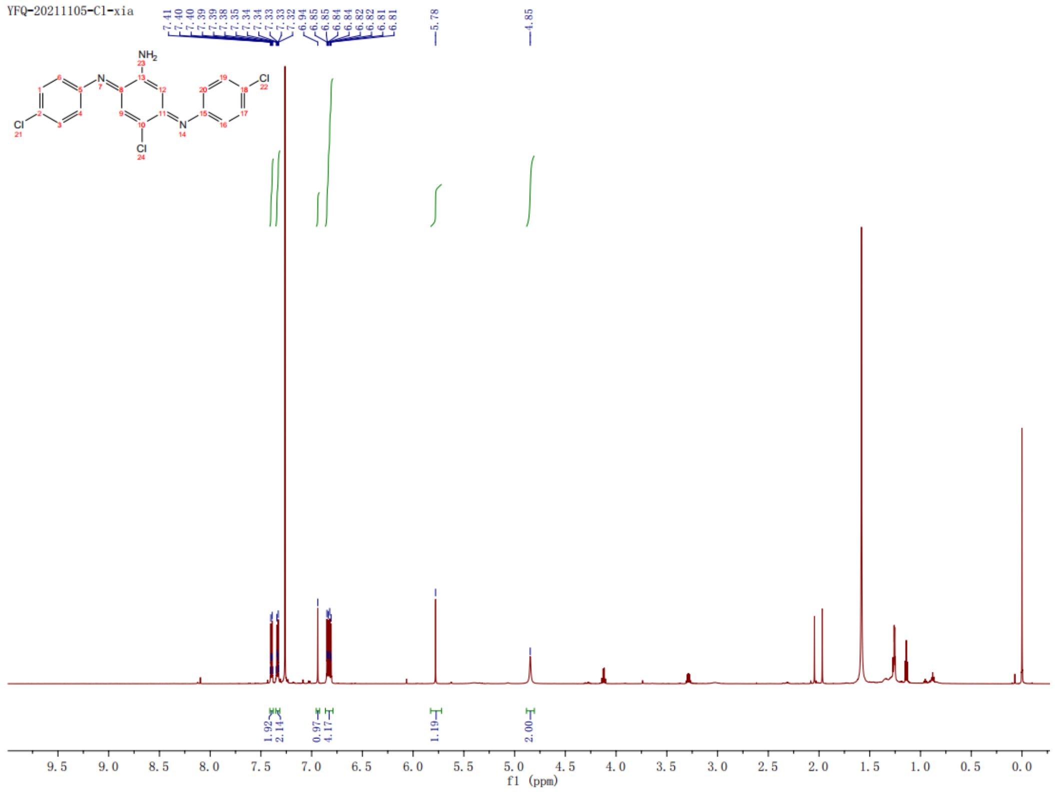

Figure S19. The spectra of ESI-MS (top) and ^{1}H NMR (bottom) for the product identification of *p*-chloroaniline. ^{1}H NMR (600 MHz， CDCl$_3$) δ = 7.41~7.38 (m, 2H), 7.35-7.32 (m, 2H), 6.94 (s, 1H), 6.85~6.81 (m, 4H), 5.78 (s, 1H), 4.85 (s, 2H), . MS (ESI): m/z [M+H]$^+$: Benzenamine,N,N'-(2-amino-5-chloro-2,5-cyclohexadiene-1,4-diylidene)bis[4-chloro- (9Cl)] ($C_{18}H_{12}Cl_3N_3$), Calculated: 375.01 Da; Observed: 376.0168 Da ([M+H]$^+$).

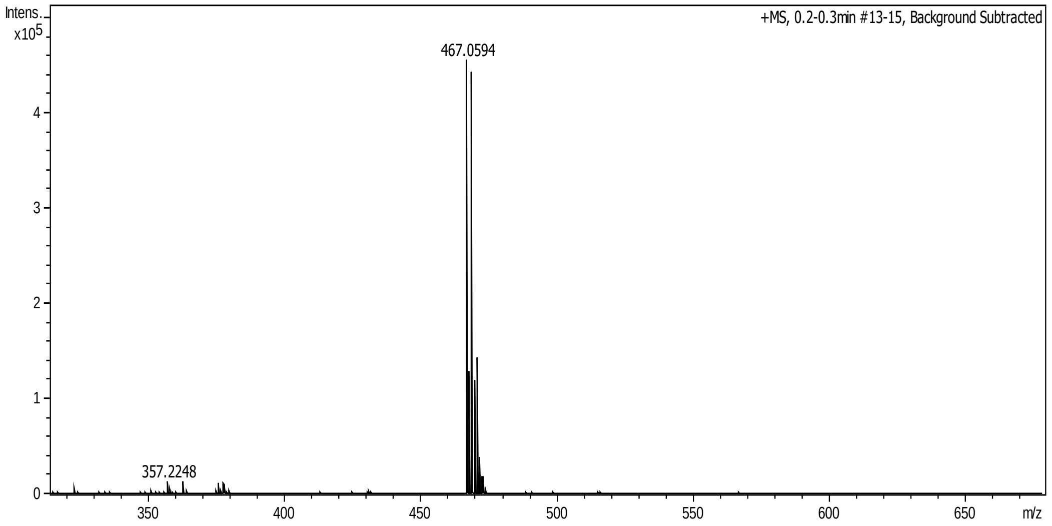

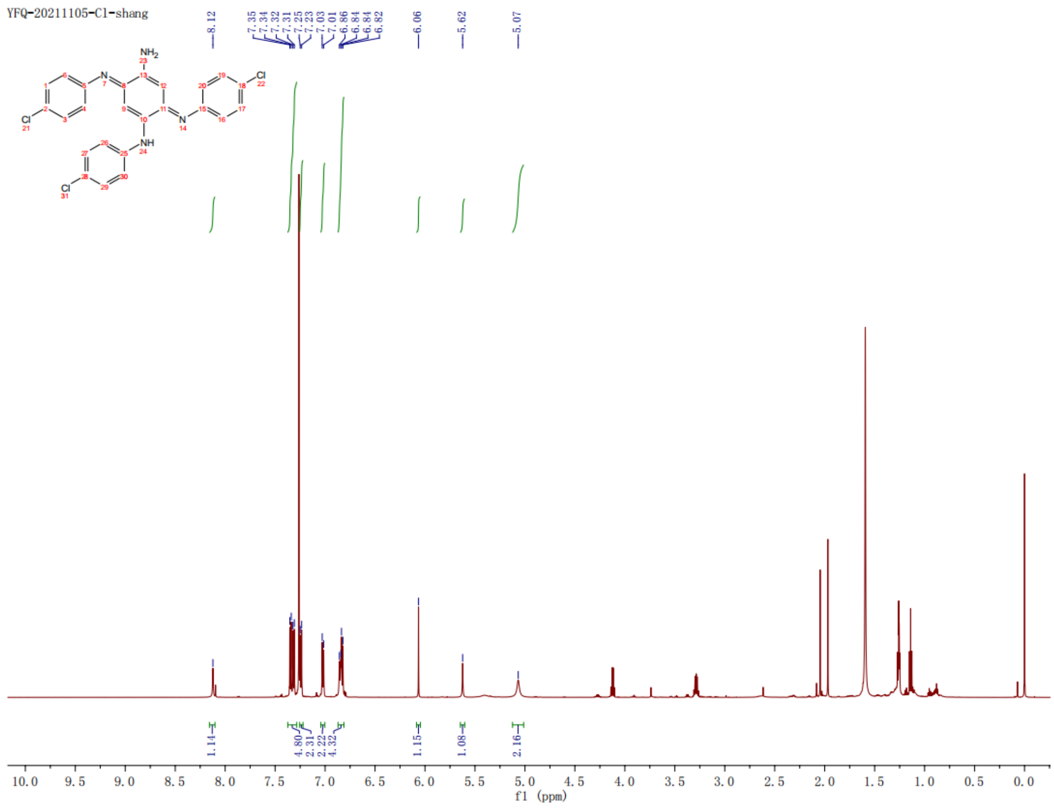

Figure S20. The spectra of ESI-MS (top) and ^{1}H NMR (bottom) for the product identification of *p*-chloroaniline. ^{1}H NMR (600 MHz, $CDCl_3$) δ = 8.12 (s, 1H), 7.35~7.31 (m, 5H), 7.25~7.23 (d, 2H, J = 12 Hz), 7.03~7.01 (d, 2H, J = 12 Hz), 6.86~6.82 (m, 4H), 6.06 (s, 1H), 5.62 (s, 1H), 5.07 (s, 2H). MS (ESI): m/z $[M+H]^+$: N1-(4-Chlorophenyl)-3,6-bis[(4-chlorophenyl)imino]-1,4-cyclohexadiene-1,4-diamine ($C_{18}H_{12}Cl_3N_4$), Calculated: 466.05 Da; Observed: 467.0594 Da ($[M+H]^+$).

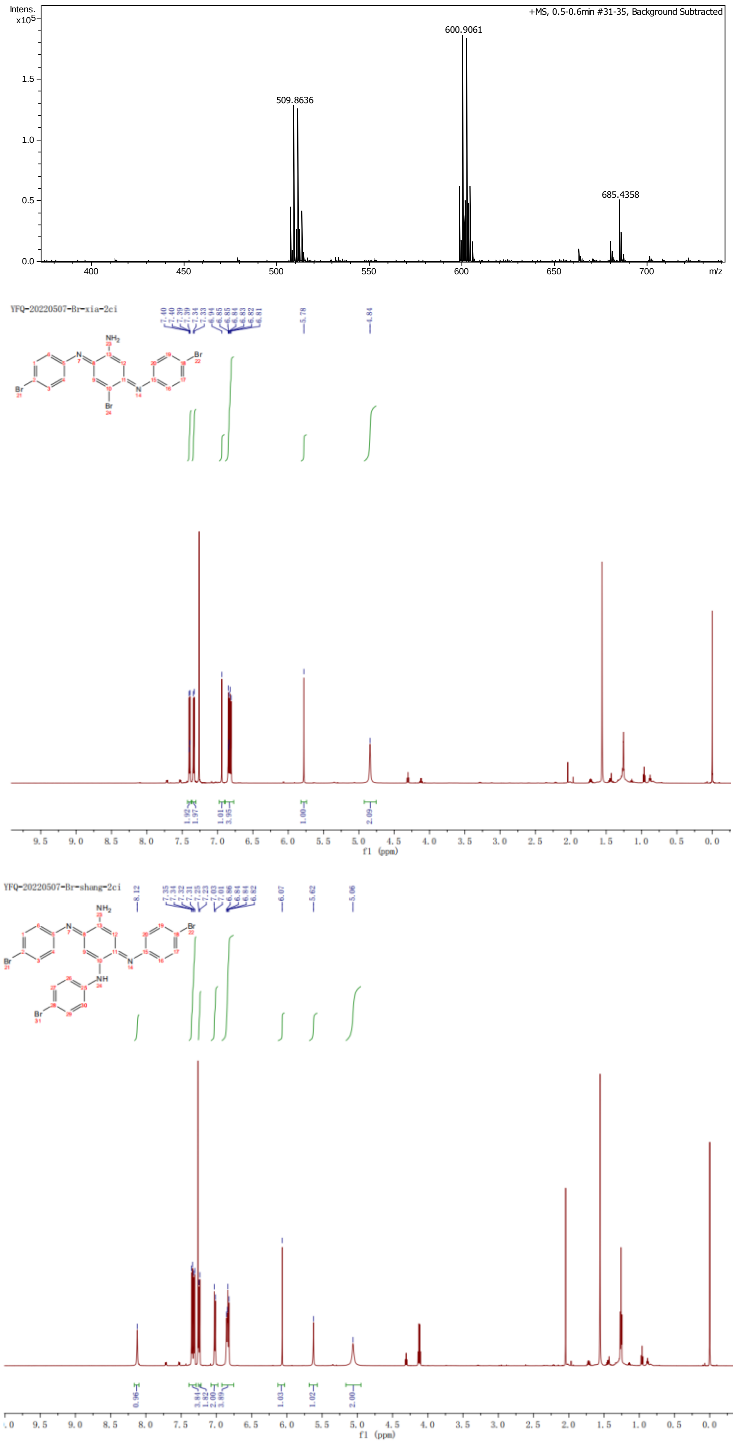

Figure S21. The spectra of ESI-MS (top) and ^{1}H NMR (bottom) for the product identification of *p*-bromoaniline. ^{1}H NMR (600 MHz, $CDCl_3$) δ = 7.4~7.39 (m, 2H), 7.34-7.33 (d, 2H, J = 6 Hz), 6.94 (s, 1H), 6.85~6.81 (m, 4H), 5.78 (s, 1H), 4.84 (s, 2H). MS (ESI): m/z $[M+H]^+$: Benzenamine,N,N'-(2-amino-5-bromo-2,5-cyclohexadiene-1,4-diylidene)bis[4-bromo- (9Br)] ($C_{18}H_{12}Br_3N_4$), Calculated: 508.86 Da; Observed: 509.8636 Da ($[M+H]^+$). ^{1}H NMR (600 MHz, d6-Me2SO) δ = 8.12 (s, 1H), 7.35~7.31 (m, 5H), 7.25~7.23 (d, 2H, J = 12 Hz), 7.03~7.01 (d, 2H, J = 12 Hz), 6.86~6.82 (m, 4H), 6.06 (s, 1H), 5.62 (s, 1H), 5.06 (s, 2H).. MS (ESI): m/z $[M+H]^+$: N1-(4-Bromophenyl)-3,6-bis[(4-bromophenyl)imino]-1,4-cyclohexadiene-1,4-diamine. ($C_{18}H_{12}Br_4N_4$), Calculated: 599.90 Da; Observed: 600.9061 Da ($[M+H]^+$).

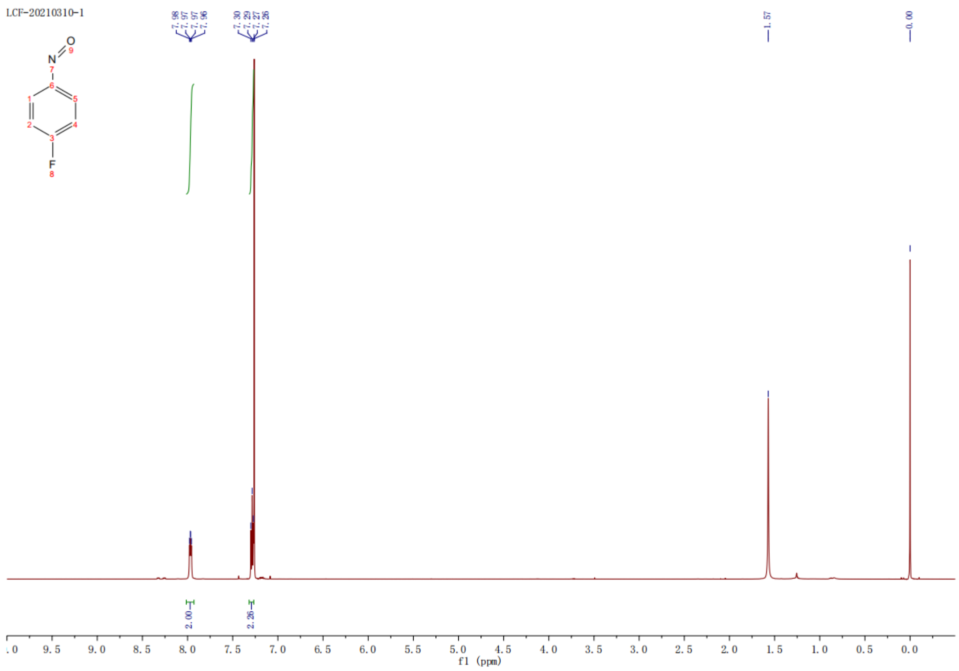

Figure S22. The spectra of ^{1}H NMR for the product identification of *p*-F-aniline. ^{1}H NMR (600 MHz, $CDCl_3$) $\delta = 7.98\sim7.96$ (m, 2H), $7.30\sim7.26$ (m, 2H): 1-Fluoro-4-nitrosobenzene (C_6H_4FNO).

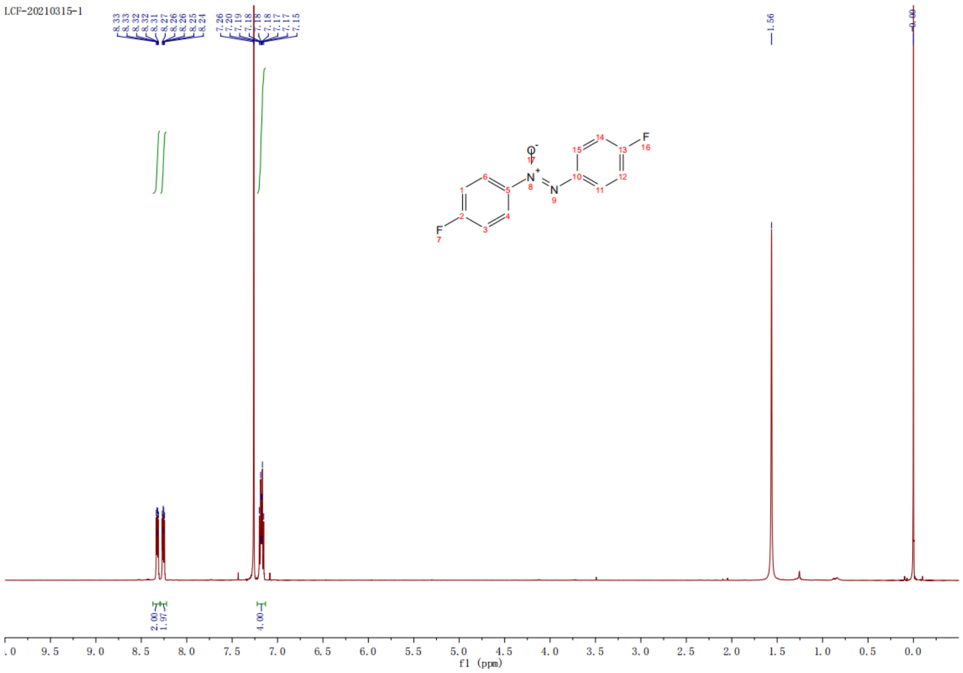

Figure S23. The spectra of ^{1}H NMR for the product identification of *p*-F-aniline. ^{1}H NMR (600 MHz, $CDCl_3$) $\delta = 8.33\sim8.24$ (m, 4H), $7.26\sim7.15$ (m, 4H): 4,4'-Difluoroazoxybenzene ($C_{12}H_8F_2N_2O$).

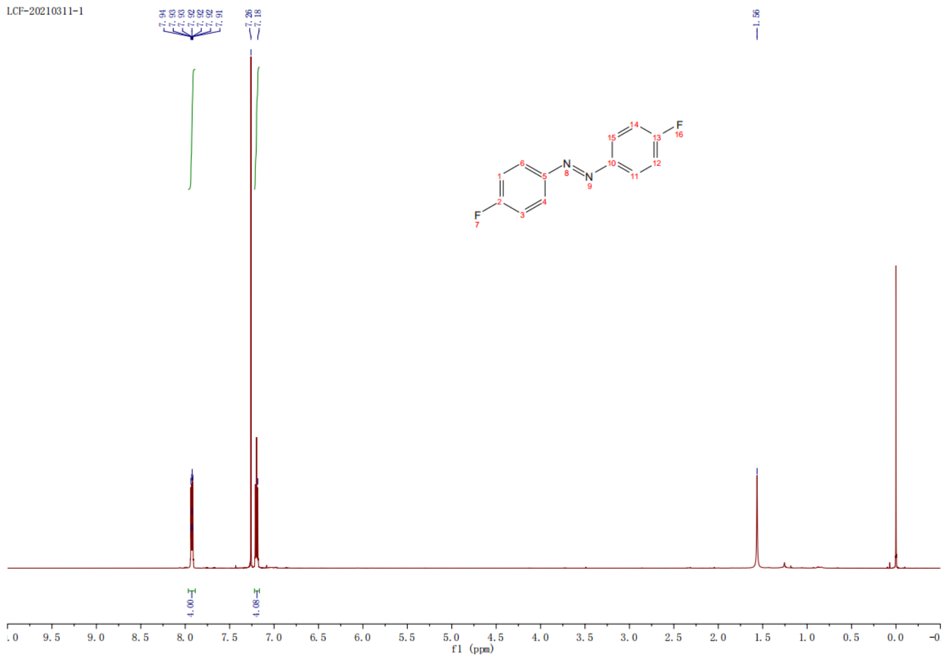

Figure S24. The spectra of ^{1}H NMR for the product identification of *p*-fluoroaniline. ^{1}H NMR (600 MHz, $CDCl_3$) δ = 7.94~7.91 (m, 4H), 7.26~7.18 (d, 4H): 4,4'-Difluoroazobenzene ($C_{12}H_8F_2N_2$).

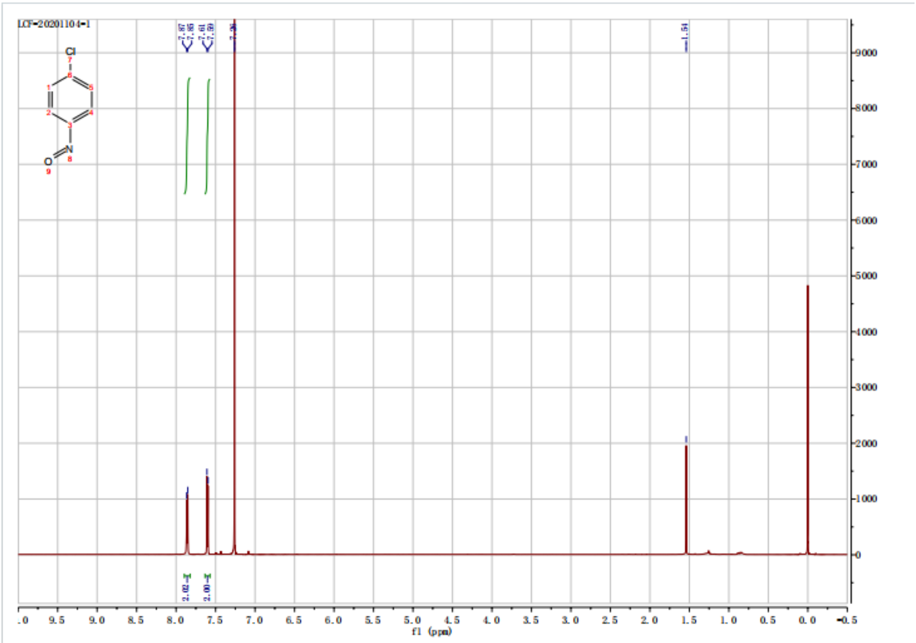

Figure S25. The spectra of ^{1}H NMR for the product identification of *p*-chloroaniline. ^{1}H NMR (600 MHz, $CDCl_3$) δ = 7.87~7.85 (d, 2H), 7.61~7.59 (d, 2H): 1- Chloro -4-nitrosobenzene (C_6H_4ClNO).

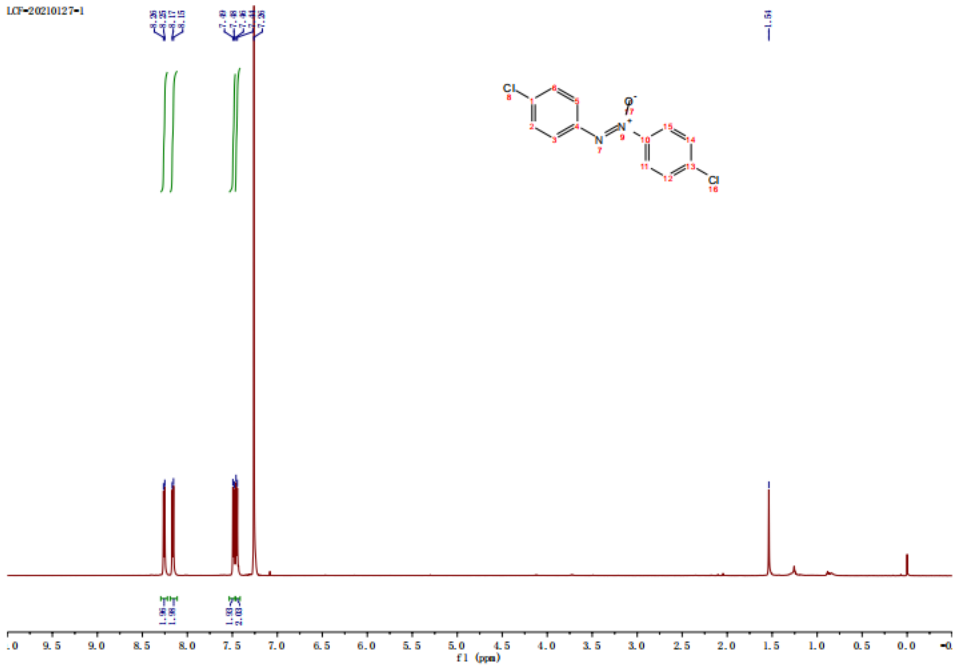

Figure S26. The spectra of ^{1}H NMR for the product identification of *p*-chloroaniline. ^{1}H NMR (600 MHz， CDCl3) δ = 8.26~8.15 (m, 4H), 7.49~7.26 (m, 4H): 4,4'-Dichloroazoxybenzene ($C_{12}H_8Cl_2N_2O$).

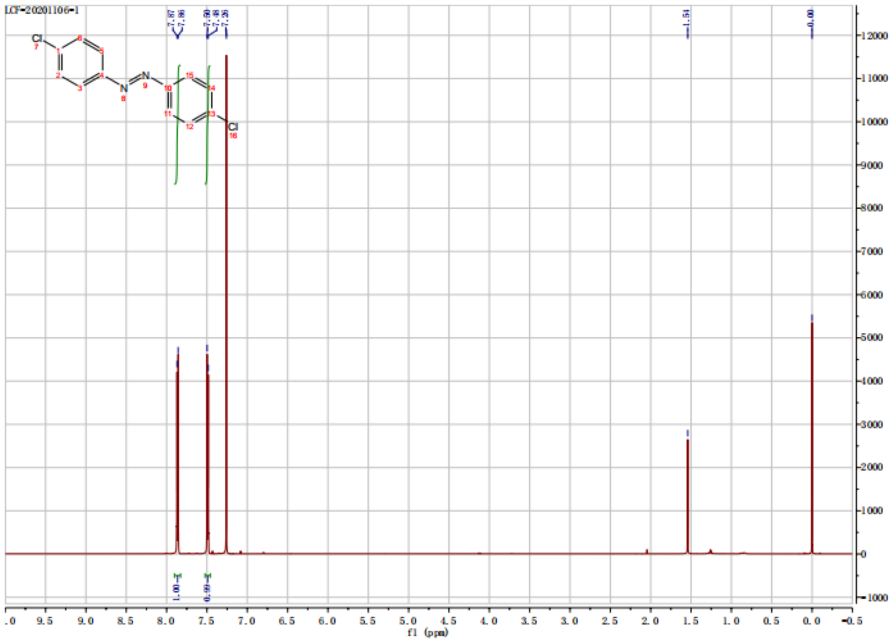

Figure S27. The spectra of ^{1}H NMR for the product identification of *p*-chloroaniline. ^{1}H NMR (600 MHz，CDCl3) δ = 7.87~7.86 (d, 4H), 7.50~7.48 (d, 4H): 4,4'-Dichloroazobenzene ($C_{12}H_8Cl_2N_2$).

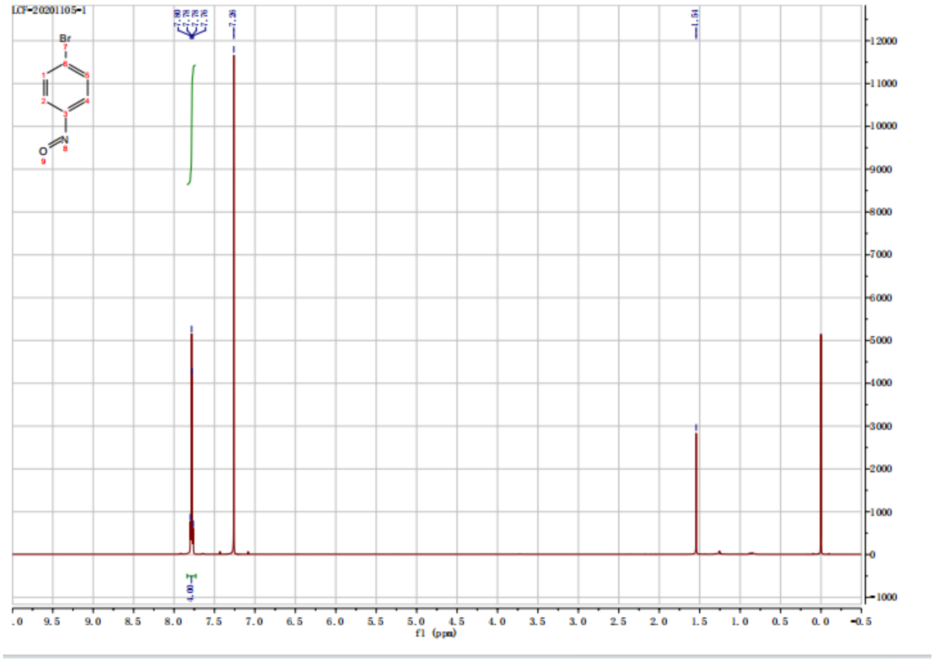

Figure S28. The spectra of ^{1}H NMR for the product identification of *p*-bromoaniline. ^{1}H NMR (600 MHz, $CDCl_3$) δ = 7.80~7.76 (m, 4H): 1- Bromo -4-nitrosobenzene (C_6H_4BrNO).

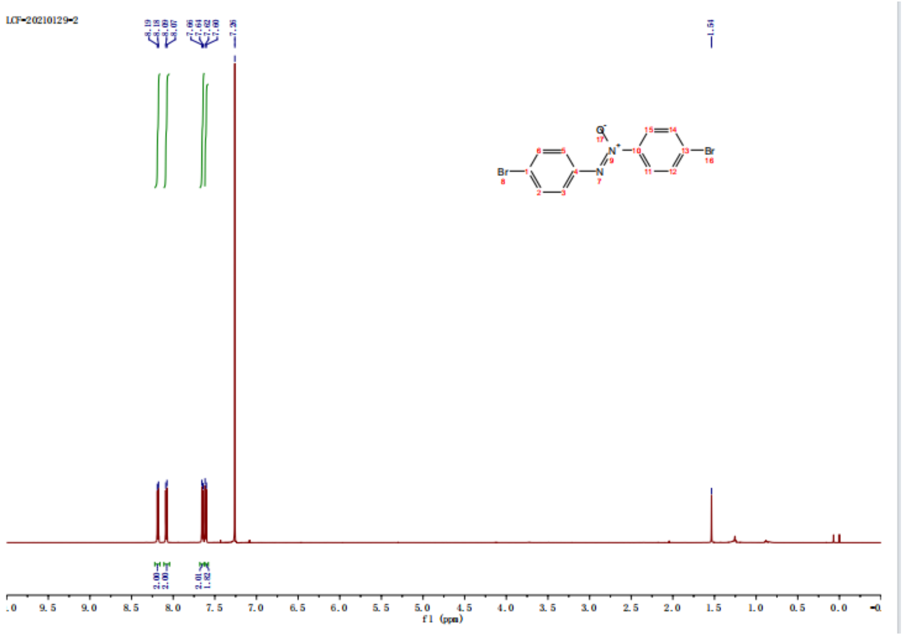

Figure S29. The spectra of ^{1}H NMR for the product identification of *p*-bromoaniline. ^{1}H NMR (600 MHz, $CDCl_3$) δ = 8.19~8.07 (m, 4H), 7.66~7.60 (m, 4H): 4,4'-Dibromoazoxybenzene ($C_{12}H_8Br_2N_2O$).

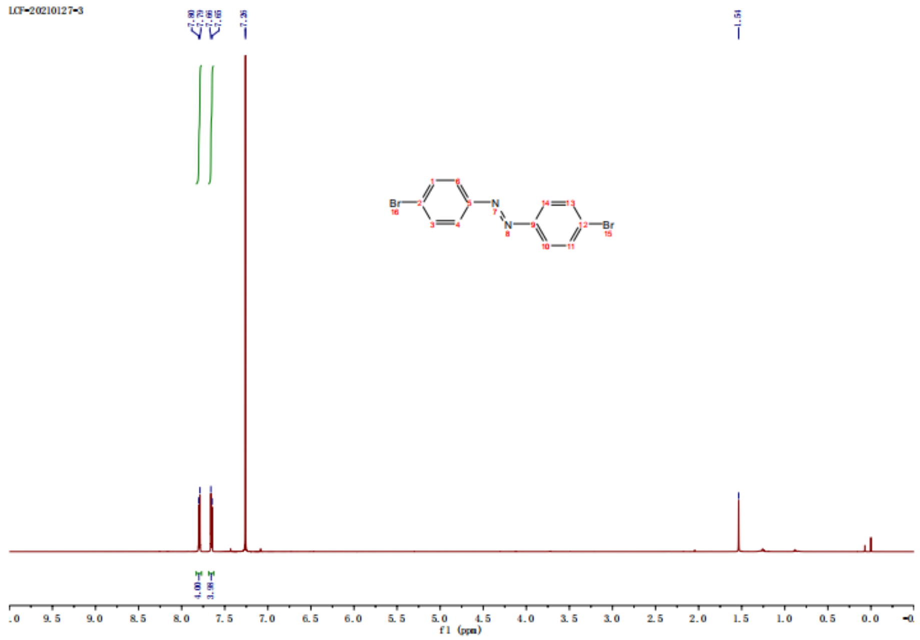

Figure S30. The spectra of ^{1}H NMR for the product identification of *p*-bromoaniline. ^{1}H NMR (600 MHz， $CDCl_3$) δ = 7.80~7.79 (d, 4H), 7.66~7.65 (d, 4H): 4,4'-Dichloroazobenzene ($C_{12}H_8Br_2N_2$).

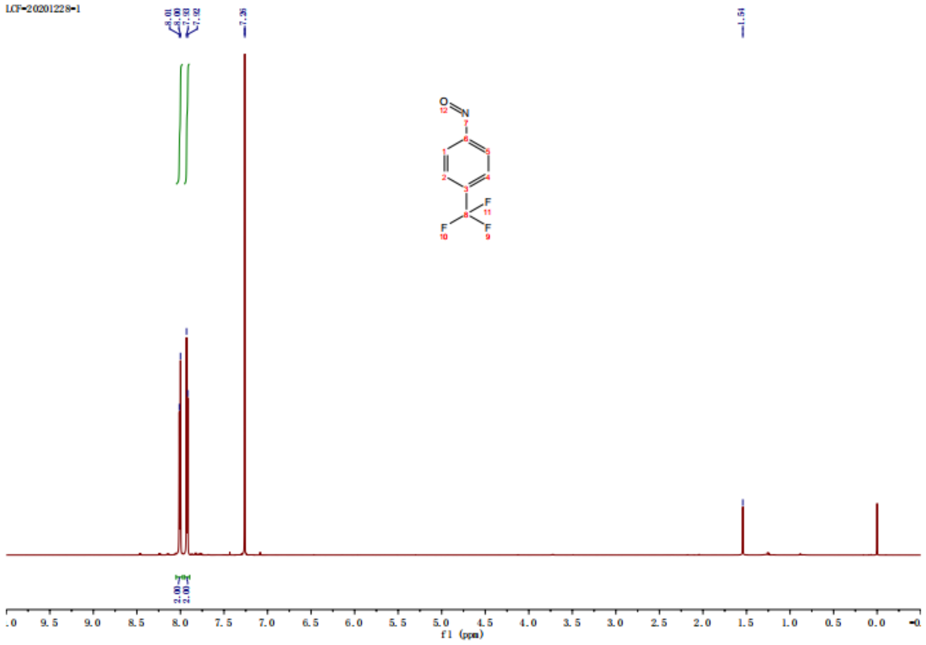

Figure S31. The spectra of ^{1}H NMR for the product identification of *p*-trifluoromethylaniline. ^{1}H NMR (600 MHz， $CDCl_3$) δ = 8.01~7.92 (m, 4H): 1-Nitroso-4-(trifluoromethyl) benzene ($C_7H_4F_3NO$).

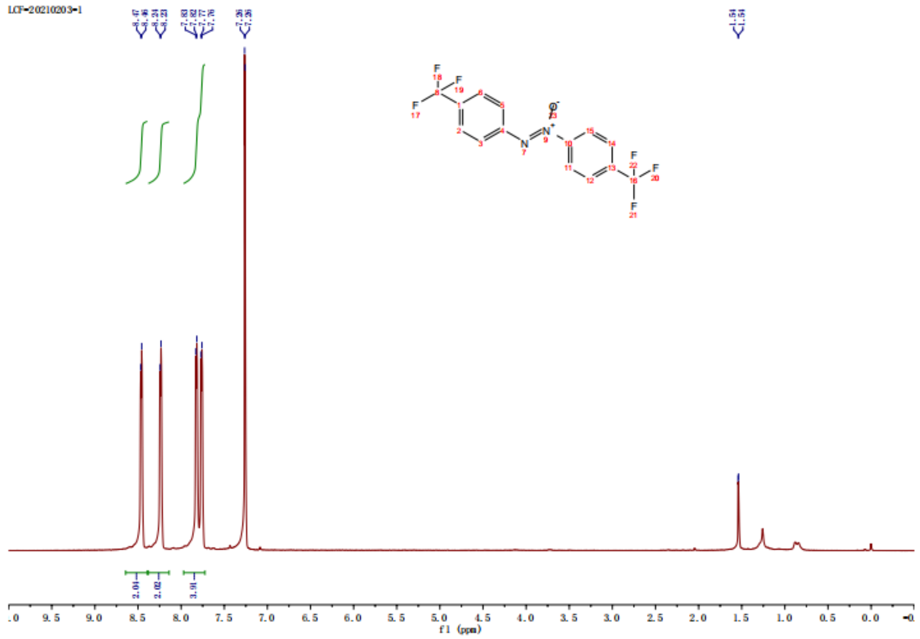

Figure S32. The spectra of ^{1}H NMR for the product identification of *p*-trifluoromethylaniline. ^{1}H NMR (600 MHz, $CDCl_3$) δ = 8.47~8.23 (m, 4H), 7.83~7.76 (m, 4H): 4,4'-Bis(trifluoromethyl)azoxybenzene ($C_{14}H_8F_6N_2O$).

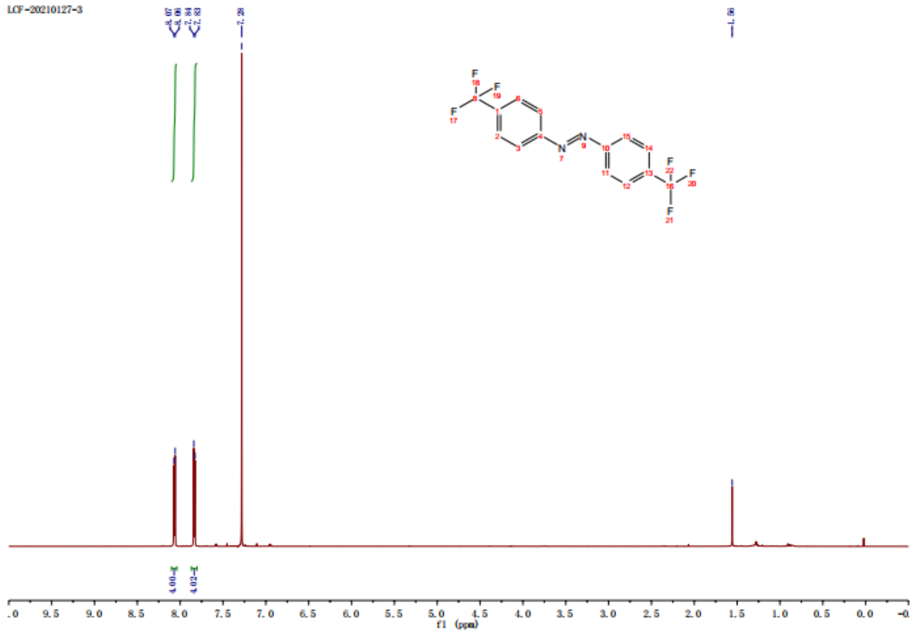

Figure S33. The spectra of ^{1}H NMR for the product identification of *p*-trifluoromethylaniline. ^{1}H NMR (600 MHz, $CDCl_3$) δ = 8.07~8.06 (d, 4H), 7.84~7.83 (d, 4H): 4,4'-Bis(trifluoromethyl)azobenzene ($C_{14}H_8F_6N_2$).

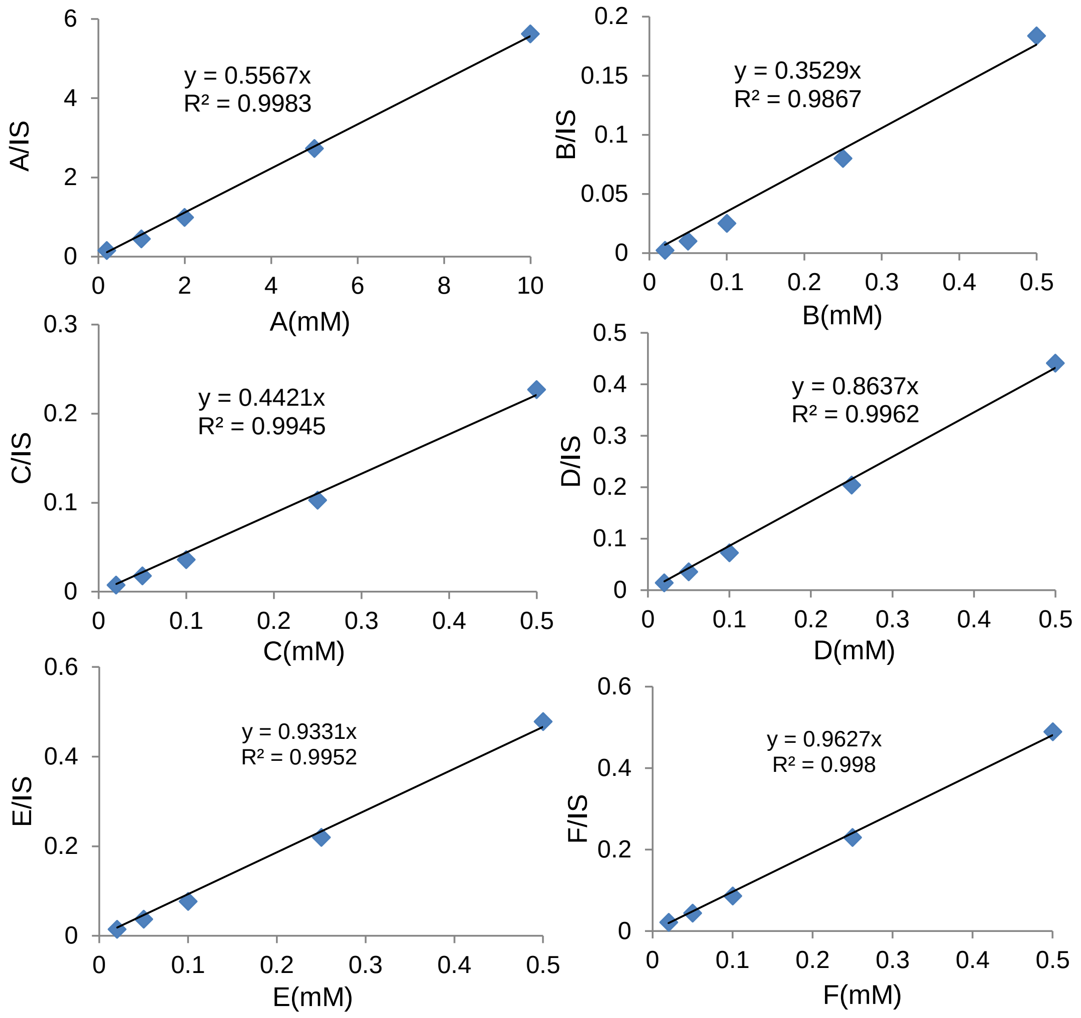

Figure S34. The calibration curves of *p*-toluidine substrates and the corresponding oxidation products. A) *p*-toluidine. B) 4-Nitrosotoluene. C) 4-Nitrotoluene. D) 4,4'-Dimethylazoxybenzene. E) Di-4-tolylamine. F) 4,4'-Dimethylazobenzene. IS: Internal standard.

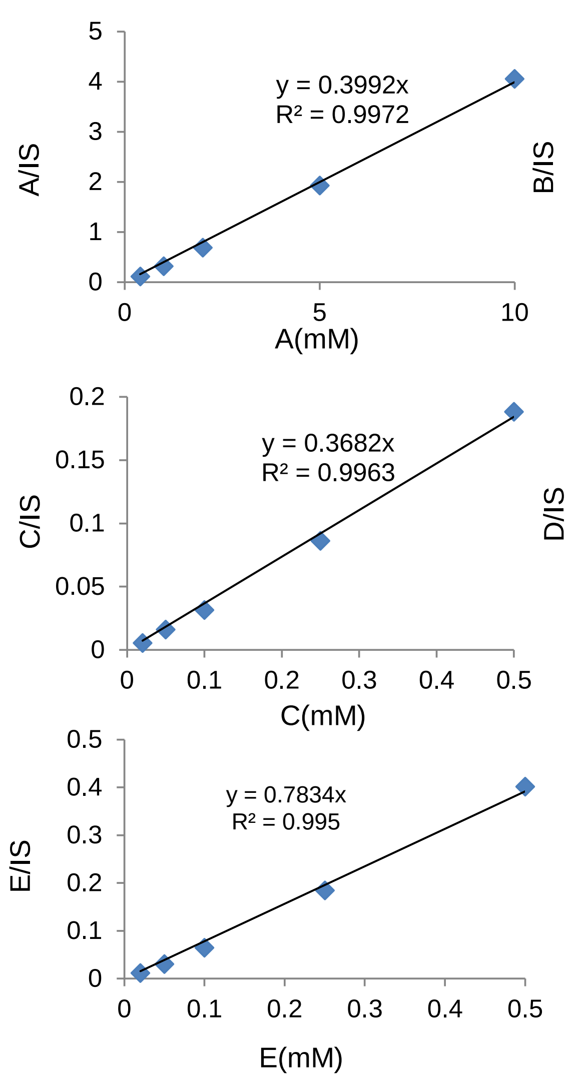

Figure S35. The calibration curves of *p*-fluoroaniline substrates and the corresponding oxidation products. A) *p*-fluoroaniline. B) 1-Fluoro-4-nitrosobenzene. C) 1-Fluoro-4-nitrobenzene. D) 4,4'-Difluoroazoxybenzene. E) 4,4'-Difluoroazobenzene. IS: Internal standard.

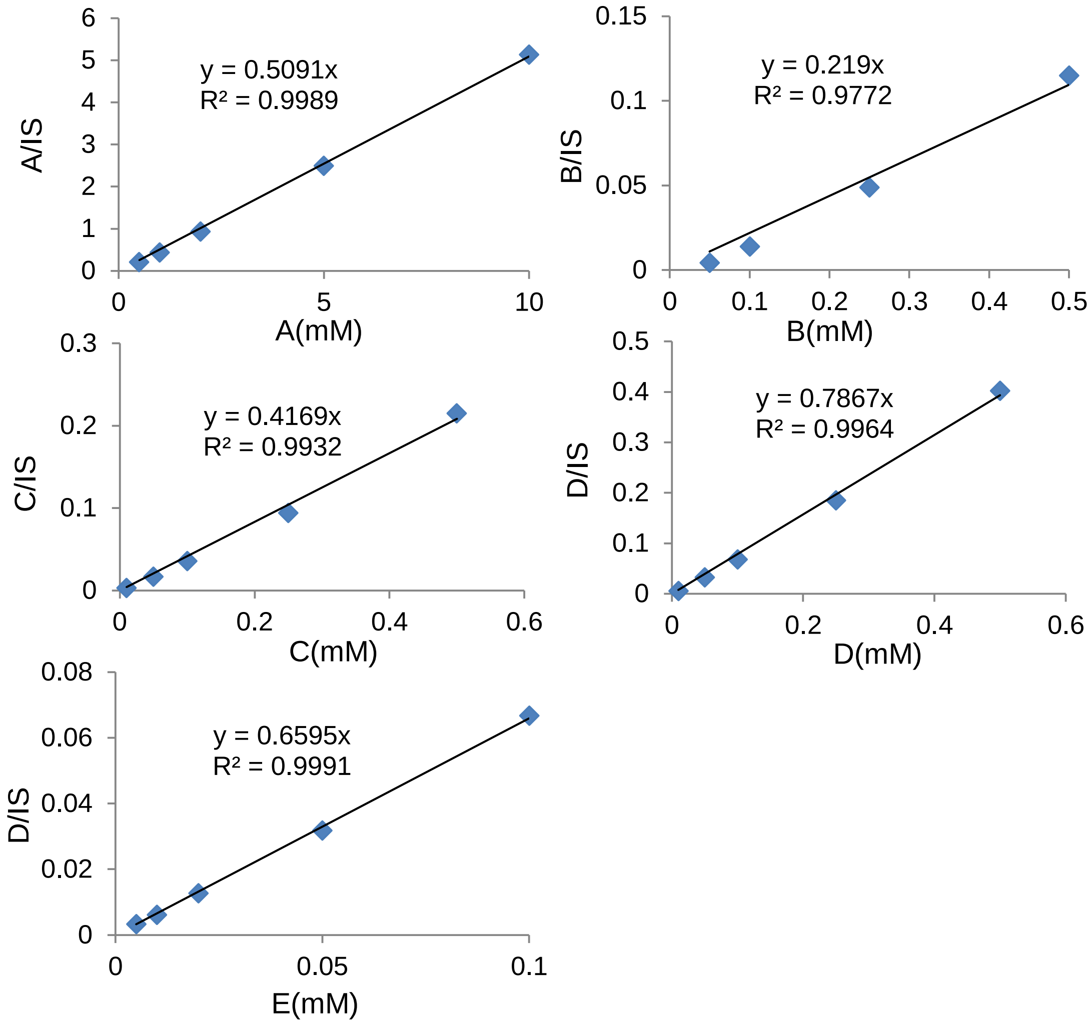

Figure S36. The calibration curves of *p*-chloroaniline substrates and the corresponding oxidation products. A) *p*-chloroaniline. B) 1-Chloro-4-nitrosobenzene. C) 1-Chloro-4-nitrobenzene. D) 4,4'-Dichloroazoxybenzene. E) 4,4'-Dichloroazobenzene. IS: Internal standard.

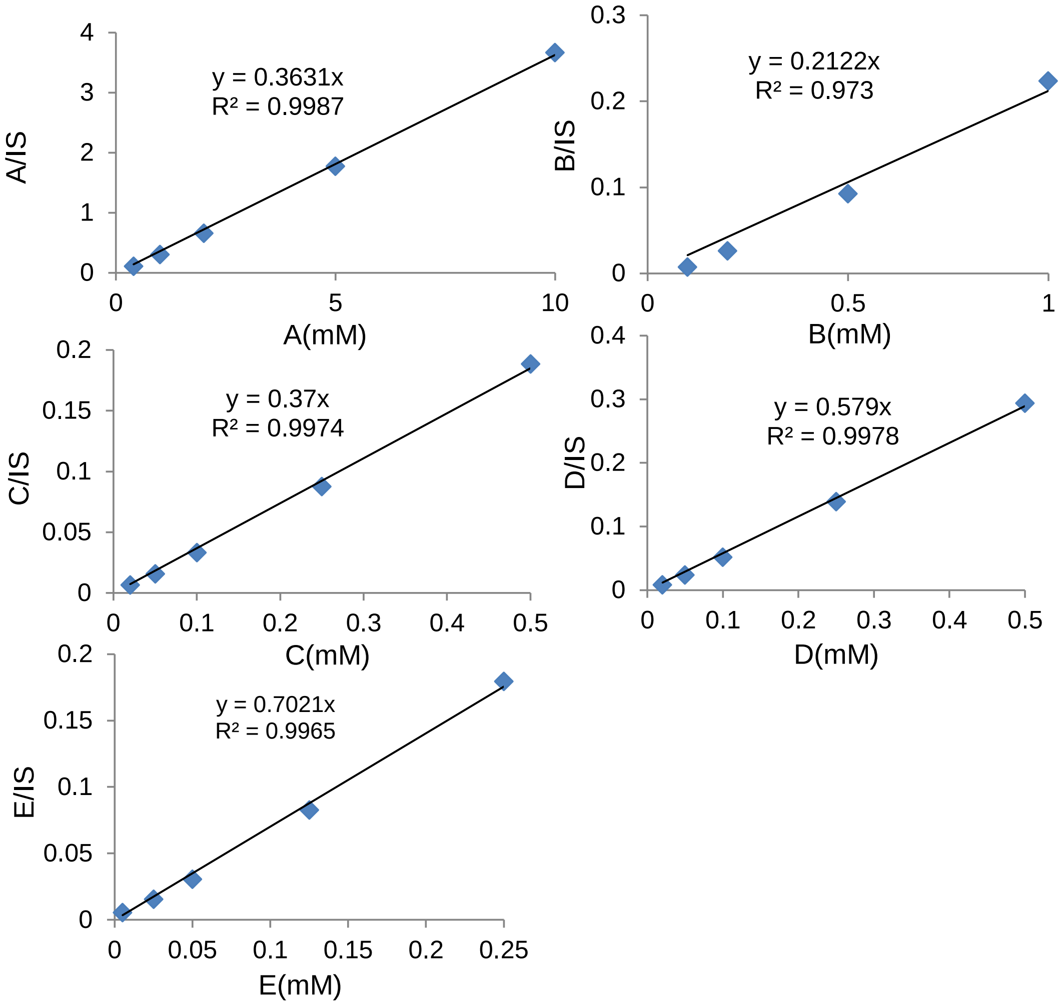

Figure S37. The calibration curves of *p*-bromoaniline substrates and the corresponding oxidation products. A) *p*-bromoaniline. B) 1-Bromo-4-nitrosobenzene. C) 1-Bromo-4-nitrobenzene. D) 4,4'-Dibromoazoxybenzene. E) 4,4'-Dichloroazobenzene. IS: Internal standard.

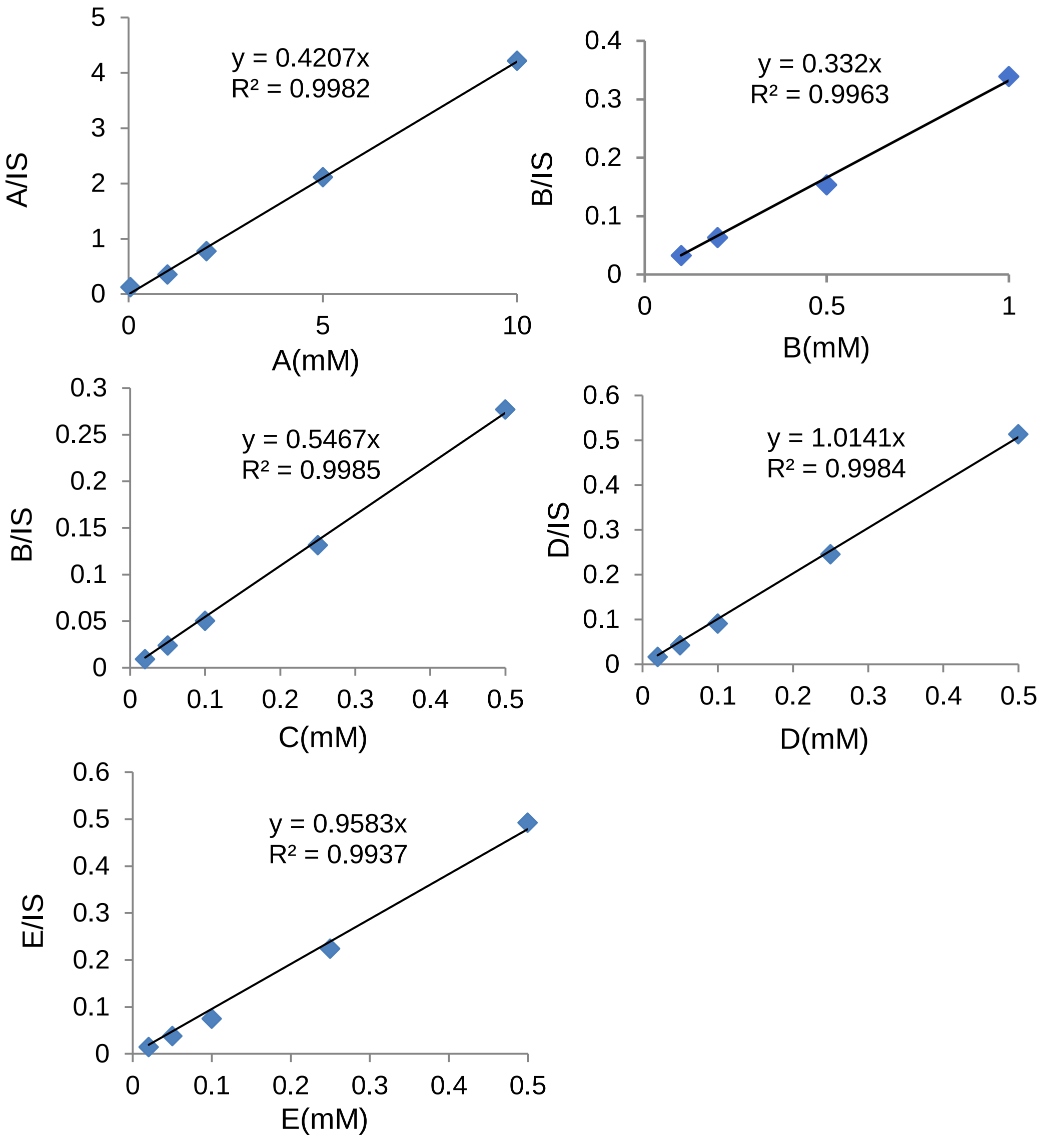

Figure S38. The calibration curves of *p*-trifluoromethylaniline substrates and the corresponding oxidation products. A) *p*-trifluoromethylaniline. B) 1-Nitroso-4-(trifluoromethyl)benzene. C) 1-Nitro-4-(trifluoromethyl)benzene. D) 4,4'-Bis(trifluoromethyl)azoxybenzene. E) 4,4'-Bis(trifluoromethyl)azobenzene. IS: Internal standard.

Supporting Tables

Table S1. The selected site in protein engineering and substituted amino acids.

position	mutations
**F87**	G, A, V, I, L, M, P, C. S, T, D, E, N, Q, R
**T268**	G, A, V, I, L, C, P, S
**L75**	V, A, M
**V78**	A, I, L, M, F, S, C, T
**A82**	G, V, L, M, S, C, T
**L181**	A, V, I, M, Q, T
**A184**	G, V, I, L, M
**L188**	A, V, I, M, F
**R255**	S, D, N
**I263**	G, A, V, L, M, Q, T
**A264**	S, V, M, E, Q
**E267**	L, S, D, Q

Table S2. The oxidation of *p*-toluidine with H_2O_2 catalyzed by F87 mutants of P450BM3 heme domain in the presence of Im-C6-Phe in pH 8.0 buffer. [a, b]

mutations	TON[c]			
	4-Nitrosotoluene	4-Nitrotoluene	Di-4-tolylamine	4,4'-Dimethylazobenzene
F87A	291±1	41±5	nd[d]	24±1
F87L	36±1	nd[d]	nd[d]	24±1
F87D	46±2	nd[d]	nd[d]	23±1
F87M	41±1	nd[d]	nd[d]	22±3
F87N	51±1	nd[d]	nd[d]	45±2
F87P	82±1	nd[d]	nd[d]	32±1
F87Q	38±1	nd[d]	nd[d]	31±2
F87T	47±1	nd[d]	nd[d]	24±1
F87V	49±1	nd[d]	nd[d]	26±2
F87S	53±1	nd[d]	nd[d]	21±1
F87I	44±1	nd[d]	nd[d]	24±1
F87G	49±1	nd[d]	nd[d]	20±2
F87E	45±1	nd[d]	nd[d]	35±1
F87C	42±1	nd[d]	nd[d]	25±5
F87H	40±1	nd[d]	nd[d]	19±1
F87R	41±1	nd[d]	nd[d]	18±1
F87K	36±1	nd[d]	nd[d]	24±2

[a] Reaction conditions: P450BM3 (0.5 μM), H_2O_2 (60 mM), Im-C6-Phe (0.5 mM), *p*-toluidine (10 mM) in pH 8.0 phosphate buffer at 25 °C. [b] All the control reactions did not show obvious activity of *p*-toluidine oxidation in the absence of Im-C6-Phe. [c] TON: Turnover numbers were estimated over a 30 min reaction. [d] nd: not detected.

Table S3. The oxidation of *p*-toluidine with H_2O_2 catalyzed by F87 mutants of P450BM3 heme domain in the presence of Im-C6-Phe in pH 7.0 buffer.[a, b]

mutations	TON[c]			
	4-Nitrosotoluene	4-Nitrotoluene	Di-4-tolylamine	4,4'-Dimethylazobenzene
F87A	52±1	nd[d]	nd[d]	20±2
F87L	13±1	nd[d]	221±1	337±3
F87D	36±1	nd[d]	8±1	27±1
F87M	32±1	nd[d]	103±1	111±1
F87N	34±2	nd[d]	142±5	177±8
F87P	49±2	nd[d]	11±1	32±1
F87Q	33±1	nd[d]	160±14	180±17
F87T	35±1	nd[d]	43±1	56±2
F87V	36±1	nd[d]	172±7	193±11
F87S	26±1	nd[d]	nd[d]	21±1
F87I	33±1	nd[d]	146±1	163±4
F87G	23±1	nd[d]	nd[d]	21±1
F87E	31±1	nd[d]	83±5	96±5
F87C	20±1	nd[d]	20±1	37±1
F87H	41±1	nd[d]	9±1	23±1
F87R	39±1	nd[d]	28±2	40±2
F87K	36±1	nd[d]	121±5	175±9

[a] Reaction conditions: P450BM3 (0.5 μM), H_2O_2 (40 mM), Im-C6-Phe (0.5 mM), *p*-toluidine (10 mM) in pH 7.0 phosphate buffer at 25 °C. [b] All the control reactions did not show obvious activity of *p*-toluidine oxidation in the absence of Im-C6-Phe. [c] TON: Turnover numbers were estimated over a 30 min reaction. [d] nd: not detected.

Table S4. The oxidation of *p*-toluidine with H_2O_2 catalyzed by F87/T268 double mutants of P450BM3 heme domain in the presence of Im-C6-Phe in pH 8.0 buffer.[a, b]

mutations	TON[c]			
	4-Nitrosotoluene	4-Nitrotoluene	Di-4-tolylamine	4,4'-Dimethylazobenzene
F87A/T268A	156±2	19±1	nd[d]	24±1
F87A/T268L	44±1	10±1	nd[d]	24±1
F87A/T268I	54±1	11±1	nd[d]	25±1
F87A/T268P	489±1	67±1	12±1	23±1
F87A/T268V	572±14	80±4	nd[d]	22±3
F87A/T268W	42±1	6±1	nd[d]	17±1
F87A/T268F	47±1	10±1	nd[d]	22±1
F87A/T268G	51±1	8±2	nd[d]	23±2
F87A/T268S	131±7	15±1	nd[d]	22±1
F87L/T268A	31±3	nd[d]	nd[d]	14±1
F87L/T268L	18±1	nd[d]	nd[d]	15±1
F87L/T268I	20±1	nd[d]	5±1	12±2
F87L/T268P	14±1	nd[d]	nd[d]	12±1
F87L/T268V	26±2	nd[d]	6±1	15±1
F87L/T268G	37±3	nd[d]	2±1	12±1
F87L/T268S	18±1	nd[d]	6±1	11±1
F87L/T268C	17±1	nd[d]	nd[d]	11±1

[a] Reaction conditions: P450BM3 (0.5 μM), H_2O_2 (60 mM), Im-C6-Phe (0.5 mM), *p*-toluidine (10 mM) in pH 8.0 phosphate buffer in 25 °C. [b] All the control reactions did not show obvious activity of *p*-toluidine oxidation in the absence of Im-C6-Phe. [c] TON: Turnover numbers were estimated over a 30 min reaction. [d] nd: not detected.

Table S5. The oxidation of *p*-toluidine with H_2O_2 catalyzed by F87L/T268 double mutants of P450BM3 heme domain in the presence of Im-C6-Phe in pH 7.0 buffer.[a, b]

mutations	TON[c]			
	4-Nitrosotoluene	4-Nitrotoluene	Di-4-tolylamine	4,4'-Dimethylazobenzene
F87L/T268A	nd[d]	nd[d]	14±1	40±1
F87L/T268L	nd[d]	nd[d]	23±1	61±3
F87L/T268I	nd[d]	nd[d]	31±1	75±2
F87L/T268P	nd[d]	nd[d]	25±1	63±1
F87L/T268V	nd[d]	nd[d]	42±1	104±3
F87L/T268G	nd[d]	nd[d]	12±1	32±3
F87L/T268S	nd[d]	nd[d]	49±1	103±1
F87L/T268C	nd[d]	nd[d]	nd[d]	22±1

[a] Reaction conditions: P450BM3 (0.5 μM), H_2O_2 (40 mM), Im-C6-Phe (0.5 mM), *p*-toluidine (10 mM) in pH 7.0 phosphate buffer at 25 °C. [b] All the control reactions did not show obvious activity of *p*-toluidine oxidation in the absence of Im-C6-Phe. [c] TON: Turnover numbers were estimated over a 30 min reaction. [d] nd: not detected.

Table S6. The oxidation of *p*-toluidine with H_2O_2 catalyzed by F87A/T268V/X triple mutants of P450BM3 heme domain in the presence of Im-C6-Phe in pH 8.0 buffer.[a, b]

mutations	TON[c]		total	Ratio[e]
	4-Nitrosotoluene	4-Nitrotoluene		
F87A/T268V/L75V	15±1	nd[d]	15±1	100:0
F87A/T268V/L75A	33±1	nd[d]	33±1	100:0
F87A/T268V/V78A	174±2	64±4	238±6	73:27
F87A/T268V/V78T	621±34	123±7	744±41	84:16
F87A/T268V/V78F	41±1	nd[d]	41±1	100:0
F87A/T268V/V78I	98±4	nd[d]	98±4	100:0
F87A/T268V/V78C	26±1	nd[d]	26±1	100:0
F87A/T268V/V78L	78±4	13±1	91±5	86:14
F87A/T268V/V78M	62±2	62±2	124±4	50:50
F87A/T268V/V78S	418	97±1	525±1	80:20
F87A/T268V/A82G	184±2	107±1	244±3	75:25
F87A/T268V/A82C	40±1	11±1	51±2	78:22
F87A/T268V/A82V	737±6	200±3	938±9	79:21
F87A/T268V/A82S	129±3	13±1	142±4	91:9
F87A/T268V/A82L	107±9	11±1	118±10	91:9
F87A/T268V/A82M	92±8	nd[d]	92±8	100:0
F87A/T268V/A82T	794±3	197±7	991±10	80:20
F87A/T268V/A82F	48±4	nd[d]	48±4	100:0
F87A/T268V/L181A	24±1	nd[d]	24±1	100:0
F87A/T268V/L181V	212±2	81±1	293±3	72:28
F87A/T268V/L181I	108±2	11±1	119±3	91:9
F87A/T268V/L181Q	146±2	14	160±2	91:9
F87A/T268V/L181T	13±1	nd[d]	13±1	100:0
F87A/T268V/L181M	93±7	nd[d]	93±7	100:0
F87A/T268V/A184G	195±17	63±7	248±24	75:25
F87A/T268V/A184V	574±3	115±1	689±4	83:17
F87A/T268V/A184L	94±4	19±1	123±5	85:15
F87A/T268V/A184M	134±16	35±3	169±19	79:21
F87A/T268V/A184I	196±2	64±2	260±4	75:25
F87A/T268V/L188A	97±1	25±1	132±2	81:19
F87A/T268V/L188M	511±5	122±6	633±11	81:19
F87A/T268V/L188F	617±7	136±2	753±9	82:18
F87A/T268V/L188I	639±31	138±12	777±43	82:18
F87A/T268V/L188V	312±16	130±8	442±24	71:29
F87A/T268V/I263G	20±1	nd[d]	20±1	100:0
F87A/T268V/I263A	60±1	10±1	70±2	86:24
F87A/T268V/I263V	101±3	27±1	128±4	79:21
F87A/T268V/I263L	832±28	330±22	1162±50	72:28
F87A/T268V/I263M	554±10	190±8	744±18	74:26
F87A/T268V/I263T	52±4	11±1	63±5	83:17

F87A/T268V/I263Q	136±2	25±1	161±3	84:16
F87A/T268V/A264E	453±5	95±1	548±6	83:17
F87A/T268V/A264M	30±1	nd[d]	30±1	100:0
F87A/T268V/A264Q	37±3	nd[d]	37±3	100:0
F87A/T268V/A264S	124±2	22	146±24	85:15
F87A/T268V/A264V	72±1	nd[d]	72±1	100:0
F87A/T268V/E267D	169±5	66±2	235±7	72:28
F87A/T268V/E267S	173±7	54±4	227±11	76:24
F87A/T268V/E267Q	679±23	171±15	850±38	80:20
F87A/T268V/E267L	133±3	30±2	163±5	82:18

[a] Reaction conditions: P450BM3 (0.5 μM), H_2O_2 (80 mM), Im-C6-Phe (0.5 mM), *p*-toluidine (10 mM) in pH 8.0 phosphate buffer at 25 °C. [b] All the control reactions did not show obvious activity of *p*-toluidine oxidation in the absence of Im-C6-Phe. [c] TON: Turnover numbers were estimated over a 30 min reaction. [d] nd: not detected. [e] 4-Nitrosotoluene: 4-Nitrotoluene.

Table S7. Effects of different pH on activity and selectivity of *p*-toluidine catalyzed reaction.[a, b]

pH	TON[c]		total	Ratio[d]
	4-Nitrosotoluene	4-Nitrotoluene		
5	25±1		25±1	100:0
6	173±3	17±1	190±4	91:9
7	672±18	103±1	776±19	87:13
8	856±2	203±1	1059±3	81:19
9	772±14	337±15	1109±29	70:30
10	354±8	541±1	894±9	40:60
10.6	50±1	732±2	782±3	6:94
11	17±1	532±14	549±15	3:97

[a] Reaction conditions: F87A/T268V/A82T (0.5 μM), H_2O_2 (80 mM), Im-C6-Phe (0.5 mM), *p*-toluidine (10 mM) in different pH buffer at 25 °C. [b] All the control reactions did not show obvious activity of *p*-toluidine oxidation in the absence of Im-C6-Phe. [c] TON: Turnover numbers were estimated over a 30 min reaction. [d] 4-Nitrosotoluene: 4-Nitrotoluene.

Table S8. Effects of different concentration of *L*-sodium ascorbate on activity and selectivity of *p*-toluidine catalyzed reaction.[a, b]

L-sodium ascorbate (mM)	TON[c]		total	4-Nitrosotoluene %
	4-Nitrosotoluene	4-Nitrotoluene		
0.5	1049±10	154±1	1203±11	87
1	1103±2	95±2	1198±4	92
2	942±1	30±1	973±2	97
5	933±3	24±1	956±4	98
10	893±20	21±1	914±21	98

[a] Reaction conditions: F87A/T268V/A82T (0.5 μM), H_2O_2 (80 mM), Im-C6-Phe (0.5 mM), *p*-toluidine (10 mM) add different concentration of *L*-sodium ascorbate at 25 °C. [b] All the control reactions did not show obvious activity of *p*-toluidine oxidation in the absence of Im-C6-Phe. [c]

TON: Turnover numbers were estimated over a 30 min reaction.

Table S9. The oxidation of *p*-toluidine with H_2O_2 catalyzed by F87A/T268V/X/Y quadruple mutants of P450BM3 heme domain in the presence of Im-C6-Phe in pH 8.0 buffer.[a, b]

mutations	TON[c]			total	Ratio[e]
	4-Nitrosotoluene	4-Nitrotoluene	4,4'-Dimethylazoxybenzene		
F87A/T268V/A82T/A184V	768±1	180±2	43±1	991±4	78:18:4
F87A/T268V/V78T/A82T	857±19	143±1	78±4	1078±24	80:13:7
F87A/T268V/V78T/A82V	482±6	76±4	26±1	584±11	82:13:5
F87A/T268V/V78T/A184V	445±3	68±1	nd[d]	513±4	87:13:0

F87A/T268V/ V78T/L188I	509±5	67±3	23±1	599±9	85:11:4
F87A/T268V/ V78T/E267Q	481±1	76±1	23±1	580±3	83:13:4
F87A/T268V/ A82T/L188I	661±9	141±5	27±1	829±15	80:17:3
F87A/T268V/ A82T/E267Q	771±49	183±13	74±11	1028±73	75:18:7
F87A/T268V/ A82V/A184V	397±10	56±2	14±1	467±13	85:12:3
F87A/T268V/ A82V/L188I	706±12	216±2	14±1	936±15	75:23:2
F87A/T268V/ A82V/E267Q	466±8	114±4	8±1	588±13	79:19:2
F87A/T268V/ A184V/L188I	503±1	89±1	29±1	621±3	81:14:5
F87A/T268V/ L188I/E267Q	584±4	99±1	37±1	720±6	81:14:5
F87A/T268V/ V78T/I263L	583±49	181±3	22±3	786±55	74:23:3
F87A/T268V/ A82T/I263L	1164±40	507±39	137±11	1808±90	64:28:8
F87A/T268V/ A82V/I263L	1001±11	409±11	58±2	1468±24	68:28:4
F87A/T268V/ A184V/I263L	989±21	593±21	140±11	1722±53	58:34:8

[a] Reaction conditions: P450BM3 (0.5 μM), H_2O_2 (80 mM), Im-C6-Phe (0.5 mM), *p*-toluidine (10 mM) in pH 8.0 phosphate buffer at 25 °C. [b] All the control reactions did not show obvious activity of *p*-toluidine oxidation in the absence of Im-C6-Phe. [c] TON: Turnover numbers were estimated over a 30 min reaction. [d] nd: not detected. [e] 4-Nitrosotoluene: 4-Nitrotoluene: 4,4'-Dimethylazoxybenzene.

Table S10. The oxidation of *p*-toluidine with H_2O_2 catalyzed by typical mutants of P450BM3 heme domain in the presence of Im-C6-Phe in 5mM *L*-sodium ascorbate pH 8.0 buffer.[a, b]

mutations	TON[c]			total	4-Nitrosotoluene %
	4-Nitrosotoluene	4-Nitrotoluene	4,4'-Dimethylazoxybenzene		
F87A/T268V/ V78T/A82T	1470±48	4±1	19±1	1493±50	98
F87A/T268V/ A82V/L188I	1615±36	3±1	22±4	1640±41	97
F87A/T268V/ A82T/I263L	2001±15	14±1	49±7	2064±23	97

F87A/T268V/ A82V/I263L	1557±29	4±1	42±2	1602±32	97

[a] Reaction conditions: P450BM3 (0.5 μM), H_2O_2 (80 mM), Im-C6-Phe (0.5 mM), *L*-sodium ascorbate (5 mM), *p*-toluidine (10 mM) in pH 8.0 phosphate buffer at 25 °C. [b] All the control reactions did not show obvious activity of *p*-toluidine oxidation in the absence of Im-C6-Phe. [c] TON: Turnover numbers were estimated over a 30 min reaction.

Table S11. The oxidation of *p*-toluidine with H_2O_2 catalyzed by typical mutants of P450BM3 heme domain in the presence of Im-C6-Phe in pH 10.6 buffer.[a, b]

mutations	TON[c]			total	4-Nitrotoluene %
	4-Nitrosotoluene	4-Nitrotoluene	4,4'-Dimethylazoxybenzene		
F87A/T268V/ V78T/A82T	37±2	851±73	nd[d]	888±75	96
F87A/T268V/ A82V/L188I	53±3	719±7	nd[d]	773±10	93
F87A/T268V/ A82T/I263L	45±1	1022±1	nd[d]	1067±2	96
F87A/T268V/ A82V/I263L	43±1	911±10	nd[d]	953±11	95

[a] Reaction conditions: P450BM3 (0.5 μM), H_2O_2 (80 mM), Im-C6-Phe (0.5 mM), *p*-toluidine (10 mM) in pH 10.6 carbonate buffer at 25 °C. [b] All the control reactions did not show obvious activity of *p*-toluidine oxidation in the absence of Im-C6-Phe. [c] TON: Turnover numbers were estimated over a 30 min reaction. [d] nd: not detected.

Table S12. The oxidation of *p*-toluidine with H_2O_2 catalyzed by F87A/T268V/V78T/A82T mutant in the presence of Im-DFSM-dipeps in 5mM *L*-sodium ascorbate pH 8.0 buffer.[a, b]

Im-DFSM-dipeps	TON[c]			total	4-Nitrosotoluene %
	4-Nitrosotoluene	4-Nitrotoluene	4,4'-Dimethylazoxybenzene		
Im-C6-Phe-Phe	1234±24	30±1	nd[d]	1264±26	98
Im-C6-Tyr-Nap	2005±4	32±2	nd[d]	2037±8	98
Im-C6-Phe(3CH_3)-Tyr	922±38	24±1	nd[d]	946±39	97

[a] Reaction conditions: F87A/T268V/V78T/A82T (0.5 μM), H_2O_2 (80 mM), Im-DFSM-dipeps (5 μM), *L*-sodium ascorbate (5 mM), *p*-toluidine (10 mM) in pH 8.0 phosphate buffer at 25 °C. [b] All the control reactions did not show obvious activity of *p*-toluidine oxidation in the absence of Im-DFSM-dipeps. [c] TON: Turnover numbers were estimated over a 30 min reaction.

Table S13. The oxidation of *p*-toluidine with H_2O_2 catalyzed by typical mutants of P450BM3 heme domain in the presence of Im-C6-Tyr-Nap in 5mM *L*-sodium ascorbate pH 8.0 buffer.[a, b]

mutations	TON[c] 4-Nitrosotoluene	4-Nitrotoluene	4,4'-Dimethylazoxybenzene	total	4-Nitrosotoluene %
F87A/T268V/A82V/I263L	2464±12	61±1	nd[d]	2525±13	98
F87A/T268V/A82T/E267Q	2170±100	123±4	nd[d]	2293±104	95
F87A/T268V/A184V/I263L	2573±47	87±11	nd[d]	2660±58	97

[a] Reaction conditions: P450BM3 (0.5 μM), H_2O_2 (80 mM), Im-C6-Phe (0.5 mM), *L*-sodium ascorbate (5 mM), *p*-toluidine (10 mM) in pH 8.0 phosphate buffer at 25 °C. [b] All the control reactions did not show obvious activity of *p*-toluidine oxidation in the absence of Im-C6-Phe. [c] TON: Turnover numbers were estimated over a 30 min reaction.

Table S14. The oxidation of *p*-toluidine with H_2O_2 catalyzed by F87A/T268V/A82T/I263L mutant in the presence of Im-DFSM-dipeps in pH 10.6 buffer.[a, b]

Im-DFSM-dipeps	TON[c] 4-Nitrosotoluene	4-Nitrotoluene	4,4'-Dimethylazoxybenzene	total	4-Nitrotoluene %
Im-C6-Phe-Phe	nd[d]	586±1	nd[d]	586±1	>99
Im-C6-Tyr-Nap	nd[d]	1221±7	nd[d]	1221±7	>99
Im-C6-Phe(3CH$_3$)-Tyr	nd[d]	335±5	nd[d]	335±5	>99

[a] Reaction conditions: P450BM3 (0.5 μM), H_2O_2 (80 mM), Im-DFSM-dipeps (5 μM), *p*-toluidine (10 mM) in pH 10.6 carbonate buffer at 25 °C. [b] All the control reactions did not show obvious activity of *p*-toluidine oxidation in the absence of Im-DFSM-dipeps. [c] TON: Turnover numbers were estimated over a 30 min reaction. [d] nd: not detected.

Table S15. The oxidation of *p*-toluidine with H_2O_2 catalyzed by F87L/X double mutants of P450BM3 heme domain in the presence of Im-C6-Phe in pH 7.0 buffer.[a, b]

mutations	TON[c] Di-4-tolylamine	4,4'-Dimethylazobenzene	N,N'-Di-p-tolyl-5-amino-2-methyl-2,5-cyclohexadiene-1,4-diimine	total	Ratio[d]
F87L	221±1	337±3	484±4	1042±8	21:32:46
F87L/V78A	394±14	560±10	709±5	1663±29	24:34:43
F87L/V78S	501±5	1047±3	1041±5	2589±13	19:41:40
F87L/V78L	185±1	275±3		460±4	40:60
F87L/V78T	410±2	628±8	770±10	1808±20	23:35:42

F87L/V78G	294±8	342±18	503±5	1139±41	26:30:44
F87L/V78C	411±11	698±30		1109±41	37:63
F87L/A82G	237±3	300±2		537±5	44:56
F87L/A82S	228±12	294±14		522±26	44:56
F87L/A82V	217±7	270±6		487±13	45:55
F87L/A82C	123±1	117±1		240±2	51:49
F87L/A82F	286±20	329±23		615±43	47:53
F87L/A82I	67±1	135±3	226±1	428±5	16:31:53
F87L/A82T	383±3	621±3	775±5	1779±9	21:35:44
F87L/A82L	457±35	893±21	1033±9	2383±65	19:38:43
F87L/A82K	363±1	640±8	814±32	1817±41	20:35:45
F87L/A82M	534±28	881±11	1071±9	2486±48	22:35:43
F87L/L181I	97±1	171±1	236±1	504±3	19:34:47
F87L/L181Q	239±1	444±10	528±10	1211±21	20:37:43
F87L/L181M	256±14	454±4	523±5	1233±23	21:37:42
F87L/L181F	266±12	461±3	566±1	1293±16	20:36:44
F87L/L181K	7±1	14±2		21±3	33:67
F87L/A184I	200±8	239±23		439±31	46:54
F87L/A184K	229±11	268±8		497±19	46:54
F87L/A184R	138±1	236±4	283±9	657±14	21:36:43
F87L/A184V	239±3	315±1	512±4	1066±8	22:30:48
F87L/A184L	153±5	218±12	324±4	695±21	22:31:47
F87L/A184M	127±1	168±2	291±19	586±22	21:29:50
F87L/L188I	214±1	358±2	485±5	1057±8	20:34:46
F87L/L188V	243±3	414±2	523±15	1180±20	21:35:44
F87L/I263L	281±3	354±6		635±9	44:56
F87L/I263V	206±6	297±3		503±9	41:59
F87L/I263M	247±1	369±15	516±1	1132±17	22:32:46
F87L/I263T	173±3	269±1	404±10	846±14	20:32:48
F87L/A264S	65±1	153±43	137±5	355±49	18:43:39
F87L/A264I	25±1	39±2	42±4	106±7	23:37:40
F87L/E267D	232±8	419±1	443±9	1094±18	21:38:41
F87L/E267Q	10±1	18±1		28±2	36:64
F87L/A328V	193±5	255±11		448±16	43:57

[a] Reaction conditions: P450BM3 (0.5 μM), H_2O_2 (40 mM), Im-C6-Phe (0.5 mM), *p*-toluidine (10 mM) in pH 7.0 phosphate buffer at 25 °C. [b] All the control reactions did not show obvious activity of *p*-toluidine oxidation in the absence of Im-C6-Phe. [c] TON: Turnover numbers were estimated over a 30 min reaction. [d] No detect. [e] Di-4-tolylamine: 4,4'-Dimethylazobenzene: N,N'-Di-p-tolyl-5-amino-2-methyl-2,5-cyclohexadiene-1,4-diimine.

Table S16. Optimization of H_2O_2 concentration used in *p*-toluidine hydroxylation catalyzed reaction.[a, b]

H_2O_2	TON[c]			total	Ratio[d]

(mM)	Di-4-tolylamine	4,4'-Dimethyl azobenzene	N,N'-Di-p-tolyl-5-amino-2-methyl-2,5-cyclohexadiene-1,4-diimine		
2.5	336±5	719±5	712±14	1767±24	19:41:40
5	482±2	1074±5	999±1	2555±8	19:42:39
7.5	533±6	1202±6	1126±5	2861±17	19:42:39
10	543±7	1227±6	1169±1	2939±14	18:42:40
20	546±6	1296±15	1225±9	3067±30	18:42:40
40	501±5	1047±3	1041±5	2589±13	19:41:40
60	405±2	747±9	811±9	1963±20	21:38:41

[a] Reaction conditions: P450BM3 F87L/V78S (0.5 μM), Im-C6-Phe (0.5 mM), *p*-toluidine (10 mM) in pH 7.0 phosphate buffer at 25 °C. [b] All the control reactions did not show obvious activity of *p*-toluidine oxidation in the absence of Im-C6-Phe. [c] TON: Turnover numbers were estimated over a 30 min reaction. [d] Di-4-tolylamine: 4,4'-Dimethylazobenzene: N, N'-Di-p-tolyl-5-amino-2-methyl-2,5-cyclohexadiene-1,4-diimine.

Table S17. Effects of different pH on activity and selectivity of *p*-toluidine catalyzed reaction.[a, b]

pH	TON[c]				
	Di-4-tolylamine	4,4'-Dimethyl azobenzene	N,N'-Di-p-tolyl-5-amino-2-methyl-2,5-cyclohexadiene-1,4-diimine	total	Ratio[d]
5.0	26±1	193±1	1343±5	1562±7	2:12:86
6.0	762±6	1459±11	1885±1	4106±18	19:35:46
7.0	561±6	1319±5	1277±14	3157±25	18:42:40
7.4	236±6	535±1	730±5	1501±12	16:39:45
8.0	59±2	128±2	330±1	517±5	14:25:64

[a] Reaction conditions: P450BM3 F87L/V78S (0.5 μM), H_2O_2 (20 mM), Im-C6-Phe (0.5 mM), *p*-toluidine (10 mM) in different pH buffer at 25 °C. [b] All the control reactions did not show obvious activity of *p*-toluidine oxidation in the absence of Im-C6-Phe. [c] TON: Turnover numbers were estimated over a 30 min reaction. [d] Di-4-tolylamine /4,4'-Dimethylazobenzene/N, N'-Di-p-tolyl-5-amino-2-methyl-2,5-cyclohexadiene-1,4-diimine.

Table S18. The oxidation of *p*-toluidine with H_2O_2 catalyzed by F87L/X/Y triple mutants of P450BM3 heme domain in the presence of Im-C6-Phe in pH 7.0 buffer.[a, b]

mutations	TON[c]				
	Di-4-tolylamine	4,4'-Dimethylazobenzene	N,N'-Di-p-tolyl-5-amino-2-methyl-2,5-cyclohexadiene-1,4-diimine	total	Ratio[d]
F87L/A82M/L188I	395±1	931±7	971±5	2297±13	17:41:42

F87L/V78T/A82T	280±1	646±1	683±9	1609±11	17:40:43
F87L/V78T/A82M	280±3	702±3	707±33	1689±39	17:41:42
F87L/V78S/A82T	437±3	938±22	990±9	2365±34	18:40:42
F87L/V78S/A82M	333±1	802±9	825±5	1959±15	17:41:42
F87L/V78S/L188V	502±9	1111±29	1131±1	2744±39	18:41:41
F87L/V78S/L181M	430±10	949±9	980±9	2360±28	18:40:42
F87L/V78S/A184V	594±1	1476±12	1409±5	3479±18	17:42:41
F87L/V78S/L188I	481±1	1182±3	1324±1	2988±5	16:40:44

[a] Reaction conditions: P450BM3 (0.5 μM), H_2O_2 (20 mM), Im-C6-Phe (0.5 mM), *p*-toluidine (10 mM) in pH 7.0 phosphate buffer at 25 °C. [b] All the control reactions did not show obvious activity of *p*-toluidine oxidation in the absence of Im-C6-Phe. [c] TON: Turnover numbers were estimated over a 30 min reaction. [d] No detect. [e] Di-4-tolylamine: 4,4'-Dimethylazobenzene: N, N'-Di-p-tolyl-5-amino-2-methyl-2,5-cyclohexadiene-1,4-diimine.

Table S19. The oxidation of *p*-toluidine with H_2O_2 catalyzed by F87L/V78S/A184V triple mutant in the presence of Im-DFSM-dipeps in pH 7.0 buffer.[a, b]

Im-DFSM-dipeps	TON[c]			total	Ratio[d]
	Di-4-tolylamine	4,4'-Dimethylazobenzene	N,N'-Di-p-tolyl-5-amino-2-methyl-2,5-cyclohexadiene-1,4-diimine		
Im-C6-Phe-Phe	283±8	674±8	970±4	1927±20	15:35:50
Im-C6-Tyr-Nap	308±12	761±25	1025±1	2094±38	15:36:47
Im-C6-Phe(3CH_3)-Tyr	205±1	393±1	558±1	1156±3	18:34:48

[a]Reaction conditions: P450BM3 (0.5 μM), H_2O_2 (20 mM), Im-DFSM-dipeps (5 μM), *p*-toluidine (10 mM) in pH 7.0 phosphate buffer at 25 °C. [b] All the control reactions did not show obvious activity of *p*-toluidine oxidation in the absence of Im-DFSM-dipeps. [c] TON: Turnover numbers were estimated over a 30 min reaction. [d] No detect. [e] Di-4-tolylamine: 4,4'-Dimethylazobenzene: N, N'-Di-p-tolyl-5-amino-2-methyl-2,5-cyclohexadiene-1,4-diimine.

Table S20. The oxidation of *p*-toluidine with H_2O_2 catalyzed by F87L/X/Y triple mutants of P450BM3 heme domain in the presence of Im-C6-Phe in pH 6.0 buffer.[a, b]

mutations	TON[c]			total	Ratio[d]
	Di-4-tolylamine	4,4'-Dimethylazobenzene	N,N'-Di-p-tolyl-5-amino-2-methyl-2,5-cyclohexadiene-1,4-diimine		

F87L/V78S/A184V	660±20	1622±27	2177±9	4459±56	15:36:49
F87L/V78S/L188V	649±11	1307±21	1871±5	3827±37	17:34:49
F87L/V78S/L188I	605±21	1155±20	1937±42	3697±83	16:31:52

[a]Reaction conditions: P450BM3 (0.5 μM), H_2O_2 (20 mM), Im-C6-Phe (0.5 mM), *p*-toluidine (10 mM) in pH 6.0 phosphate buffer at 25 °C. [b] All the control reactions did not show obvious activity of *p*-toluidine oxidation in the absence of Im-C6-Phe. [c] TON: Turnover numbers were estimated over a 30 min reaction. [d] No detect. [e] Di-4-tolylamine: 4,4'-Dimethylazobenzene: N, N'-Di-p-tolyl-5-amino-2-methyl-2,5-cyclohexadiene-1,4-diimine.

Table S21. The oxidation of *p*-fluoroaniline with H_2O_2 catalyzed by typical mutants of P450BM3 heme domain in the presence of Im-C6-Phe in pH 8.0 buffer [a, b]

mutations	TON[c]			total	Ratio[e]
	1-Fluoro-4-nitrosobenzene	1-Fluoro-4-nitrobenzene	4,4'-Difluoroazoxybenzene		
F87A/T268V	871±37	210±9	19±1	1099±47	79:19:2
F87A/T268V/A82T	856±12	265±3	239±6	1360±21	63:19:18
F87A/T268V/A82T/A184V	833±1	195±7	10±1	1038±9	80:19:1
F87A/T268V/A82T/L188I	721±14	176±2	5±1	902±17	80:19:1
F87A/T268V/A82V/L188I	902±14	179 ±3	nd[d]	1081±17	83:17:0
F87A/T268V/A82T/I263L	926±3	160±3	58±3	1114±9	81:14:5

[a]Reaction conditions: P450BM3 (0.5 μM), H_2O_2 (40 mM), Im-C6-Phe (0.5 mM), *p*-fluoroaniline (10 mM) in pH 8.0 phosphate buffer at 25 °C. [b] All the control reactions did not show obvious activity of *p*-fluoroaniline oxidation in the absence of Im-C6-Phe. [c] TON: Turnover numbers were estimated over a 30 min reaction. [d] nd: not detected. [e] Ratio: 1-Fluoro-4-nitrosobenzene: 1-Fluoro-4-nitrobenzene: 4,4'-Fluoroazoxybenzene.

Table S22. The oxidation of *p*-fluoroaniline with H_2O_2 catalyzed by typical mutants of P450BM3 heme domain in the presence of Im-C6-Phe in 5mM *L*-sodium ascorbate pH 8.0 buffer.[a, b]

mutations	TON[c]			total	1-Fluoro-4-nitrosob
	1-Fluoro-4-nitrosoben	1-Fluoro-4-nitrobenz	4,4'-Difluoroazoxybenz		

	zene	ene	ene		enzene %
F87A/T268V	1483±55	61±1	92±8	1636±64	91
F87A/T268V/A82T	909±11	nd[d]	30±3	939±14	97
F87A/T268V/A82V/L188I	990±22	25±9	78±2	1093±33	91
F87A/T268V/A82T/I263L	1092±23	17±1	22±1	1131±25	97

[a] Reaction conditions: P450BM3 (0.5 μM), H_2O_2 (80 mM), Im-C6-Phe (0.5 mM), *L*-sodium ascorbate (5 mM), *p*-fluoroaniline (10 mM) in pH 8.0 phosphate buffer at 25 °C. [b] All the control reactions did not show obvious activity of *p*-fluoroaniline oxidation in the absence of Im-C6-Phe. [c] TON: Turnover numbers were estimated over a 30 min reaction. [d] nd: not detected.

Table S23. The oxidation of *p*-fluoroaniline with H_2O_2 catalyzed by typical mutants of P450BM3 heme domain in the presence of Im-C6-Phe in pH 10.6 buffer.[a, b]

mutations	TON[c]			total	1-Fluoro-4-nitrobenzene %
	1-Fluoro-4-nitrosobenzene	1-Fluoro-4-nitrobenzene	4,4'-Difluoroazoxybenzene		
F87A/T268V	nd[d]	635±47	nd[d]	635±47	>99
F87A/T268V/A82T	nd[d]	724±18	nd[d]	724±18	>99
F87A/T268V/A82V/L188I	nd[d]	315±1	nd[d]	315±1	>99
F87A/T268V/A82T/I263L	nd[d]	917±5	nd[d]	917±5	>99

[a] Reaction conditions: P450BM3 (0.5 μM), H_2O_2 (80 mM), Im-C6-Phe (0.5 mM), *p*-fluoroaniline (10 mM) in pH 10.6 carbonate buffer at 25 °C. [b] All the control reactions did not show obvious activity of *p*-fluoroaniline oxidation in the absence of Im-C6-Phe. [c] TON: Turnover numbers were estimated over a 30 min reaction. [d] nd: not detected.

Table S24. The oxidation of *p*-fluoroaniline with H_2O_2 catalyzed by F87A/T268V/A82T/I63L mutant in the presence of Im-DFSM-dipeps in 5mM *L*-sodium ascorbate pH 8.0 buffer.[a, b]

Im-DFSM-dipeps	TON[c]			total	1-Fluoro-4-nitrosobenzene %
	1-Fluoro-4-nitrosobenzene	1-Fluoro-4-nitrobenzene	4,4'-Difluoroazoxybenzene		
Im-C6-Phe-Phe	2279±17	nd[d]	nd[d]	2279±17	>99
Im-C6-Tyr-Nap	1991±13	nd[d]	nd[d]	1991±13	>99
Im-C6-Phe(3CH$_3$)-Tyr	1786±21	nd[d]	nd[d]	1786±21	>99

[a] Reaction conditions: P450BM3 (0.5 μM), H_2O_2 (80 mM), Im-DFSM-dipeps (5 μM), *L*-sodium

ascorbate (5 mM), *p*-fluoroaniline (10 mM) in pH 8.0 phosphate buffer at 25 °C. [b] All the control reactions did not show obvious activity of *p*-fluoroaniline oxidation in the absence of Im-DFSM-dipeps. [c] TON: Turnover numbers were estimated over a 30 min reaction.

Table S25. The oxidation of *p*-fluoroaniline with H_2O_2 catalyzed by F87A/T268V/A82T/I263L mutant in the presence of Im-DFSM-dipeps in pH 10.6 buffer.[a, b]

Im-DFSM-dipeps	TON[c]				1-Fluoro-4-nitrobenzene %
	1-Fluoro-4-nitrosobenzene	1-Fluoro-4-nitrobenzene	4,4'-Difluoroazoxybenzene	total	
Im-C6-Phe-Phe	nd[d]	658±16	nd[d]	658±16	>99
Im-C6-Tyr-Nap	nd[d]	1129±1	nd[d]	1129±1	>99
Im-C6-Phe(3CH$_3$)-Tyr	nd[d]	418±3	nd[d]	418±3	>99

[a] Reaction conditions: P450BM3 (0.5 μM), H_2O_2 (80 mM), Im-DFSM-dipeps (5 μM), *p*-fluoroaniline (10 mM) in pH 10.6 carbonate buffer at 25 °C. [b] All the control reactions did not show obvious activity of *p*-fluoroaniline oxidation in the absence of Im-DFSM-dipeps. [c] TON: Turnover numbers were estimated over a 30 min reaction. [d] nd: not detected.

Table S26. The oxidation of *p*-fluoroaniline with H_2O_2 catalyzed by typical mutants of P450BM3 heme domain in the presence of Im-C6-Phe in pH 7.0 buffer.[a, b]

mutations	TON[c]	
	4,4'-Difluoroazobenzene	*N*[1]-(4-Fluorophenyl)-3,6-bis[(4-fluorophenyl)imino]-1,4-cyclohexadiene-1,4-diamine
F87L	127±1	375±1
F87L/A82M	203±1	568±1
F87L/V78S	288±24	722±1
F87L/L181M	172±1	384±2
F87L/L188V	189±4	436±1
F87L/V78S/L188V	306±7	717±1
F87L/V78S/A184V	373±11	777±5

[a] Reaction conditions: P450BM3 (0.5 μM), H_2O_2 (20 mM), Im-C6-Phe (0.5 mM), *p*-fluoroaniline (10 mM) in pH 7.0 phosphate buffer at 25 °C. [b] All the control reactions did not show obvious activity of *p*-fluoroaniline oxidation in the absence of Im-C6-Phe. [c] TON: Turnover numbers were estimated over a 30 min reaction.

Table S27. The oxidation of *p*-fluoroaniline with H_2O_2 catalyzed by F87L/V78S/A184V triple mutant in the presence of Im-DFSM-dipeps in pH 7.0 buffer.[a, b]

Im-DFSM-dipeps	TON[c]	
	4,4'-Difluoroazobenzene	*N*[1]-(4-Fluorophenyl)-3,6-bis[(4-fluorophenyl)imino]-1,4-cyclohexadiene-1,4-diamine

Im-C6-Phe-Phe	147±5	468±2
Im-C6-Tyr-Nap	155±3	509±3
Im-C6-Phe(3CH$_3$)-Tyr	90±11	300±2

[a] Reaction conditions: P450BM3 (0.5 μM), H_2O_2 (40 mM), Im-DFSM-dipeps (5 μM), *p*-fluoroaniline (10 mM) in pH 7.0 phosphate buffer at 25 °C. [b] All the control reactions did not show obvious activity of *p*-fluoroaniline oxidation in the absence of Im-DFSM-dipeps. [c] TON: Turnover numbers were estimated over a 30 min reaction.

Table S28. The oxidation of *p*-chloroaniline with H_2O_2 catalyzed by typical mutants of P450BM3 heme domain in the presence of Im-C6-Phe in pH 8.0 buffer.[a, b]

mutations	TON[c]			total	Ratio[d]
	1-Chloro-4-nitrosobenzene	1-Chloro-4-nitrobenzene	4,4'-Dichloroazoxybenzene		
F87A/T268V	314±9	303±15	241±12	858±36	37:36:17
F87A/T268V/A82T	270±1	317±3	769±1	1356±5	20:23:57
F87A/T268V/A82T/A184V	560±10	547±13	1048±75	2155±98	26:25:49
F87A/T268V/A82T/L188I	343±2	360±4	311±1	1014±7	34:36:30
F87A/T268V/A82V/L188I	436±29	494±33	112±18	1042±80	42:47:11
F87A/T268V/A82T/I263L	522±15	174±11	208±28	904±54	58:19:23

[a] eaction conditions: P450BM3 (0.5 μM), H_2O_2 (80 mM), Im-C6-Phe (0.5 mM), *p*-chloroaniline (10 mM) in pH 8.0 phosphate buffer at 25 °C. [b] All the control reactions did not show obvious activity of *p*-chloroaniline oxidation in the absence of Im-C6-Phe. [c] TON: Turnover numbers were estimated over a 30 min reaction. [d] Ratio: 1-Chloro-4-nitrosobenzene: 1-Chloro-4-nitrobenzene: 4,4'-Dichloroazoxybenzene.

Table S29. The oxidation of *p*-chloroaniline with H_2O_2 catalyzed by typical mutants of P450BM3 heme domain in the presence of Im-C6-Phe in 5mM *L*-sodium ascorbate pH 8.0 buffer.[a, b]

mutations	TON[c]			total	1-Chloro-4-nitrosobenzene %
	1-Chloro-4-nitrosobenzene	1-Chloro-4-	4,4'-Dichloroazoxyb		

		nitroben zene	enzene		
F87A/T268V/A82T/A184V	2301±169	28±1	82±2	2411±172	95
F87A/T268V/A82V/L188I	1727±43	15±1	37	1779±3	97
F87A/T268V/A82T/I263L	1340±22	56±23	25±2	1421±47	94

[a] Reaction conditions: P450BM3 (0.5 μM), H_2O_2 (80 mM), Im-C6-Phe (0.5 mM), *L*-sodium ascorbate (5 mM), *p*-chloroaniline (10 mM) in pH 8.0 phosphate buffer at 25 °C. [b] All the control reactions did not show obvious activity of *p*-chloroaniline oxidation in the absence of Im-C6-Phe. [c] TON: Turnover numbers were estimated over a 30 min reaction.

Table S30. The oxidation of *p*-chloroaniline with H_2O_2 catalyzed by typical mutants of P450BM3 heme domain in the presence of Im-C6-Phe in pH 10.6 buffer.[a, b]

mutations	TON[c]			total	1-Chloro-4-nitrobenzene %
	1-Chloro-4-nitrosobenzene	1-Chloro-4-nitrobenzene	4,4'-Dichloroazoxybenzene		
F87A/T268V/A82T/A184V	52±3	1025±44	nd[d]	1078±47	95
F87A/T268V/A82V/L188I	nd[d]	219±10	nd[d]	219±10	>99
F87A/T268V/A82T/I263L	nd[d]	1430±23	32±3	1463±26	98

[a] Reaction conditions: P450BM3 (0.5 μM), H_2O_2 (80 mM), Im-C6-Phe (0.5 mM), *p*-chloroaniline (10 mM) in pH 10.6 carbonate buffer at 25 °C. [b] All the control reactions did not show obvious activity of *p*-chloroaniline oxidation in the absence of Im-C6-Phe. [c] TON: Turnover numbers were estimated over a 30 min reaction. [d] nd: not detected

Table S31. The oxidation of *p*-chloroaniline with H_2O_2 catalyzed by F87A/T268V/A82T/A184V mutant in the presence of Im-DFSM-dipeps in 5mM *L*-sodium ascorbate pH 8.0 buffer.[a, b]

Im-DFSM-dipeps	TON[c]			total 1-Chloro-4-nitrosobenzene	1-Chloro-4-nitrosobenzene % 1-Chloro-4-nitrobenze
	1-Chloro-4-nitrosobenzene	1-Chloro-4-nitrobenzene	4,4'-Dichloroazoxybenzene		

					ne
Im-C6-Phe-Phe	3542±92	48±1	48±4	3638±97	97
Im-C6-Tyr-Nap	4274±13	30±1	77±7	4381±21	98
Im-C6-Phe(3CH$_3$)-Tyr	3173±21	42±1	59±1	3274±23	97

[a] Reaction conditions: P450BM3 (0.5 μM), H_2O_2 (80 mM), Im-DFSM-dipeps (5 μM), *L*-sodium ascorbate (5 mM), *p*-chloroaniline (10 mM) in pH 8.0 phosphate buffer at 25 °C. [b] All the control reactions did not show obvious activity of *p*-chloroaniline oxidation in the absence of Im-DFSM-dipeps. [c] TON: Turnover numbers were estimated over a 30 min reaction.

Table S32. The oxidation of *p*-chloroaniline with H_2O_2 catalyzed by F87A/T268V/A82T/I63L mutant in the presence of Im-DFSM-dipeps in pH 10.6 buffer.[a, b]

Im-DFSM-dipeps	TON[c]			total	1-Chloro-4-nitrobenzene %
	1-Chloro-4-nitrosobenzene	1-Chloro-4-nitrobenzene	4,4'-Dichloroazoxybenzene		
Im-C6-Phe-Phe	nd[d]	977±28	nd[d]	977±28	>99
Im-C6-Tyr-Nap	nd[d]	1583±4	nd[d]	1583±4	>99
Im-C6-Phe(3CH$_3$)-Tyr	nd[d]	607±28	nd[d]	607±28	>99

[a] Reaction conditions: P450BM3 (0.5 μM), H_2O_2 (80 mM), Im-DFSM-dipeps (5 μM), *p*-chloroaniline (10 mM) in pH 10.6 carbonate buffer at 25 °C. [b] All the control reactions did not show obvious activity of *p*-chloroaniline oxidation in the absence of Im-DFSM-dipeps. [c] TON: Turnover numbers were estimated over a 30 min reaction. [d] nd: not detected.

Table S33. The oxidation of *p*-chloroaniline with H_2O_2 catalyzed by typical mutants of P450BM3 heme domain in the presence of Im-C6-Phe in pH 7.0 buffer.[a, b]

mutations	TON[c]		
	4,4'-Dichloroazobenzene	Benzenamine,*N,N'*-(2-amino-5-chloro-2,5-cyclohexadiene-1,4-diylidene)bis[4-chloro-(9Cl)]	*N*[1]-(4-Chlorophenyl)-3,6-bis[(4-chlorophenyl)imino]-1,4-cyclohexadiene-1,4-diamine
F87L	108±3	90±1	72±1
F87L/A82M	215±1	188±1	151±1

F87L/V78S	152±1	165±9	132±7
F87L/L181M	93±3	62±9	50±7
F87L/L188V	116±6	72±10	58±8
F87L/V78S/L188V	177±8	135±3	108±3
F87L/V78S/A184V	198±3	193±24	154±19

[a] Reaction conditions: P450BM3 (0.5 μM), H_2O_2 (40 mM), Im-C6-Phe (0.5 mM), *p*-chloroaniline (10 mM) in pH 7.0 phosphate buffer at 25 °C. [b] All the control reactions did not show obvious activity of *p*-chloroaniline oxidation in the absence of Im-C6-Phe. [c] TON: Turnover numbers were estimated over a 30 min reaction.

Table S34. The oxidation of *p*-chloroaniline with H_2O_2 catalyzed by F87L/V78S/A184V triple mutant in the presence of Im-DFSM-dipeps in pH 7.0 buffer.[a, b]

Im-DFSM-dipeps	TON[c]		
	4,4'-Dichloroazobenzene	Benzenamine,*N,N'*-(2-amino-5-chloro-2,5-cyclohexadiene-1,4-diylidene)bis[4-chloro-(9Cl)]	*N*[1]-(4-Chlorophenyl)-3,6-bis[(4-chlorophenyl)imino]-1,4-cyclohexadiene-1,4-diamine
Im-C6-Phe-Phe	55±1	187±5	112±2
Im-C6-Tyr-Nap	61±1	174±1	104±1
Im-C6-Phe(3CH$_3$)-Tyr	50±1	136±5	82±2

[a] Reaction conditions: P450BM3 (0.5 μM), H_2O_2 (40 mM), Im-DFSM-dipeps (5 μM), *p*-chloroaniline (10 mM) in pH 7.0 phosphate buffer at 25 °C. [b] All the control reactions did not show obvious activity of *p*-chloroaniline oxidation in the absence of Im-DFSM-dipeps. [c] TON: Turnover numbers were estimated over a 30 min reaction.

Table S35. The oxidation of *p*-bromoaniline with H_2O_2 catalyzed by typical mutants of P450BM3 heme domain in the presence of Im-C6-Phe in pH 8.0 buffer.[a, b]

mutations	TON[c]			total	Ratio[e]
	1-Bromo-4-nitrosobenzene	*1*-Bromo-4-nitrobenzene	4,4'-Dibromoazoxybenzene		
F87A/T268V	400±2	232±5	170±5	802±12	50:29;21
F87A/T268V/A82T	659±21	269±6	598±20	1526±47	43:18:39

F87A/T268V/A82T/A184V	861±31	530±2	1384±10	2775±43	31:28:41
F87A/T268V/A82T/L188I	451±16	347±7	351±3	1148±26	39:30:31
F87A/T268V/A82V/L188I	366±39	429±64	107±14	902±117	41:47:12
F87A/T268V/A82T/I263L	281±2	217±3	289±41	788±46	36:28:36

[a] Reaction conditions: P450BM3 (0.5 μM), H_2O_2 (40 mM), Im-C6-Phe (0.5 mM), (10 mM) in pH 8.0 phosphate buffer at 25 °C. [b] All the control reactions did not show obvious activity of *p*-bromoaniline oxidation in the absence of Im-C6-Phe. [c] TON: Turnover numbers were estimated over a 30 min reaction. [e] Ratio: 1-Bromo-4-nitrosobenzene: 1-Bromo-4-nitrobenzene: 4,4'-Bromoroazoxybenzene.

Table S36. The oxidation of *p*-bromoaniline with H_2O_2 catalyzed by typical mutants of P450BM3 heme domain in the presence of Im-C6-Phe in 5mM *L*-sodium ascorbate pH 8.0 buffer.[a, b]

mutations	TON[c]			total	*1*-Bromo-4-nitrosobenzene %
	1-Bromo-4-nitrosobenzene	*1*-Bromo-4-nitrobenzene	4,4'-Dibromoazoxybenzene		
F87A/T268V/A82T/A184V	2312±31	74±1	165±16	2550±48	91
F87A/T268V/A82V/L188I	1372±59	nd[d]	42±5	1415±64	97
F87A/T268V/A82T/I263L	802±100	71±45	28±15	901±160	89

[a] Reaction conditions: P450BM3 (0.5 μM), H_2O_2 (80 mM), Im-C6-Phe (0.5 mM), *L*-sodium ascorbate (5 mM), *p*-bromoaniline (10 mM) in pH 8.0 phosphate buffer at 25 °C. [b] All the control reactions did not show obvious activity of *p*-bromoaniline oxidation in the absence of Im-C6-Phe. [c] TON: Turnover numbers were estimated over a 30 min reaction. [d] nd: not detected.

Table S37. The oxidation of *p*-bromoaniline with H_2O_2 catalyzed by typical mutants of P450BM3 heme domain in the presence of Im-C6-Phe in pH 10.6 buffer.[a, b]

mutations	TON[c]			total	1-Bromo-4-nitrobenzene %
	1-Bromo-4-nitrosobenzene	1-Bromo-4-nitrobenzene	4,4'-Dibromoazoxybenzene		

F87A/T268V/ A82T/A184V	88±1	2133±128	24±4	2200±133	97
F87A/T268V/ A82V/L188I	nd[d]	519±54	nd[d]	519±54	>99
F87A/T268V/ A82T/I263L	nd[d]	1101±138	21±5	1123±143	98

[a] Reaction conditions: P450BM3 (0.5 μM), H_2O_2 (80 mM), Im-C6-Phe (0.5 mM), *p*-bromoaniline (10 mM) in pH 10.6 carbonate buffer at 25 °C. [b] All the control reactions did not show obvious activity of *p*-bromoaniline oxidation in the absence of Im-C6-Phe. [c] TON: Turnover numbers were estimated over a 30 min reaction. [d] nd: not detected.

Table S38. The oxidation of *p*-bromoaniline with H_2O_2 catalyzed by F87A/T268V/A82T/A184V mutant in the presence of Im-DFSM-dipeps in 5mM *L*-sodium ascorbate pH 8.0 buffer.[a, b]

Im-DFSM-dipeps	TON[c]			total	*1*-Bromo-4-nitrosobenzene %
	1-Bromo-4-nitrosobenzene	*1*-Bromo-4-nitrobenzene	4,4'-Dibromoazoxybenzene		
Im-C6-Phe-Phe	3279±43	88±2	nd[d]	3367±45	97
Im-C6-Tyr-Nap	4220±102	47±1	nd[d]	4267±103	99
Im-C6-Phe(3CH$_3$)-Tyr	3295±131	74±1	nd[d]	3369±132	98

[a] Reaction conditions: P450BM3 (0.5 μM), H_2O_2 (80 mM), Im-DFSM-dipeps (5 μM), *L*-sodium ascorbate (5 mM), *p*-bromoaniline (10 mM) in pH 8.0 phosphate buffer at 25 °C. [b] All the control reactions did not show obvious activity of *p*-bromoaniline oxidation in the absence of Im-DFSM-dipeps. [c] TON: Turnover numbers were estimated over a 30 min reaction.

Table S39. The oxidation of *p*-bromoaniline with H_2O_2 catalyzed by F87A/T268V/A82T/A184V mutant in the presence of Im-DFSM-dipeps in pH 10.6 buffer.[a, b]

Im-DFSM-dipeps	TON[c]			total	1-Bromo-4-nitrobenzene %
	1-Bromo-4-nitrosobenzene	1-Bromo-4-nitrobenzene	4,4'-Dibromoazoxybenzene		
Im-C6-Phe-Phe	nd[d]	1420±112	nd[d]	1420±112	>99
Im-C6-Tyr-Nap	nd[d]	1845±153	nd[d]	1845±153	>99

Im-C6-Phe(3CH_3)-Tyr	nd[d]	885±19	nd[d]	885±19	>99

[a] Reaction conditions: P450BM3 (0.5 μM), H_2O_2 (80 mM), Im-DFSM-dipeps (5 μM), *p*-bromoaniline (10 mM) in pH 10.6 carbonate buffer at 25 °C. [b] All the control reactions did not show obvious activity of *p*-bromoaniline oxidation in the absence of Im-DFSM-dipeps. [c] TON: Turnover numbers were estimated over a 30 min reaction. [d] nd: not detected.

Table S40. The oxidation of *p*-bromoaniline with H_2O_2 catalyzed by typical mutants of P450BM3 heme domain in the presence of Im-C6-Phe in pH 7.0 buffer.[a, b]

mutations	TON[c]		
	4,4'-Dibroroazobenzene	Benzenamine,*N,N'*-(2-amino-5-bromo-2,5-cyclohexadiene-1,4-diylidene)bis[4-bromo-(9Br)]	*N*[1]-(4-Bromophenyl)-3,6-bis[(4-bromophenyl)imino]-1,4-cyclohexadiene-1,4-diamine
F87L	49±1	63±6	76±2
F87L/A82M	78±1	99±1	120±1
F87L/V78S	94±2	119±7	144±2
F87L/L181M	49±1	62±5	75±2
F87L/L188V	64±1	81±7	99±3
F87L/V78S/L188V	86±1	109±1	132±1
F87L/V78S/A184V	113±1	143±1	174±1

[a] Reaction conditions: P450BM3 (0.5 μM), H_2O_2 (40 mM), Im-C6-Phe (0.5 mM), *p*-bromoaniline (10 mM) in pH 7.0 phosphate buffer at 25 °C. [b] All the control reactions did not show obvious activity of *p*-bromoaniline oxidation in the absence of Im-C6-Phe. [c] TON: Turnover numbers were estimated over a 30 min reaction.

Table S41. The oxidation of *p*-bromoaniline with H_2O_2 catalyzed by F87L/V78S/A184V triple mutant in the presence of Im-DFSM-dipeps in pH 7.0 buffer.[a, b]

Im-DFSM-dipeps	TON[c]		
	4,4'-Dibroroazobenzene	Benzenamine,*N,N'*-(2-amino-5-bromo-2,5-cyclohexadiene-	*N*[1]-(4-Bromophenyl)-3,6-bis[(4-

		1,4-diylidene)bis[4-bromo-(9Br)]	bromophenyl)imino]-1,4-cyclohexadiene-1,4-diamine
Im-C6-Phe-Phe	31±1	66±2	79±2
Im-C6-Tyr-Nap	32±1	55±2	66±1
Im-C6-Phe(3CH_3)-Tyr	26±1	45±1	54±2

[a] Reaction conditions: P450BM3 (0.5 μM), H_2O_2 (40 mM), Im-DFSM-dipeps (5 μM), *p*-bromoaniline (10 mM) in pH 7.0 phosphate buffer at 25 °C. [b] All the control reactions did not show obvious activity of *p*-bromoaniline oxidation in the absence of Im-DFSM-dipeps. [c] TON: Turnover numbers were estimated over a 30 min reaction.

Table S42. The oxidation of *p*-trifluoromethylaniline with H_2O_2 catalyzed by typical mutants of P450BM3 heme domain in the presence of Im-C6-Phe in pH 8.0 buffer.[a, b]

mutations	TON[c]			total	Ratio[e]
	1-Nitroso-4-(trifluoromethyl)benzene	*1*-Nitro-4-(trifluoromethyl)benzene	4,4'-Bis(trifluoromethyl)azoxybenzene		
F87A/T268V	337±20	210±10	115±7	662±37	51:32:17
F87A/T268V/A82T	555±33	255±22	273±46	1082±101	51:24:25
F87A/T268V/A82T/A184V	429±15	501±19	326±23	1255±57	34:40:26
F87A/T268V/A82T/L188I	270±5	214±2	88±4	572±11	47:37:16
F87A/T268V/A82V/L188I	nd[d]	229±33	58±17	286±50	0:80:20
F87A/T268V/A82T/I263L	284±2	240±9	214±1	737±12	39:32:29

[a] Reaction conditions: P450BM3 (0.5 μM), H_2O_2 (80 mM), Im-C6-Phe (0.5 mM), *p*-trifluoromethylaniline (10 mM) in pH 8.0 phosphate buffer at 25 °C. [b] All the control reactions did not show obvious activity of *p*-trifluoromethylaniline oxidation in the absence of Im-C6-Phe. [c] TON: Turnover numbers were estimated over a 30 min reaction. [d] nd: not detected. [e]Ratio:1-Nitroso-4-(trifluoromethyl) benzene: 1-Nitro-4-(trifluoromethyl) benzene: 4,4'-Bis (trifluoromethyl) azoxybenzene.

Table S43. The oxidation of *p*-trifluoromethylaniline with H_2O_2 catalyzed by typical mutants of P450BM3 heme domain in the presence of Im-C6-Phe in 5mM *L*-sodium ascorbate pH 8.0 buffer.[a, b]

mutations	TON[c]			total	1-Nitroso-4-(trifluoromethyl)benzene %
	1-Nitroso-4-(trifluoromethyl)benzene	1-Nitro-4-(trifluoromethyl)benzene	4,4'-Bis(trifluoromethyl)azoxybenzene		
F87A/T268V/A82T	1579±28	38±2	52±1	1669±31	95
F87A/T268V/A82T/A184V	1753±13	136±1	65±1	1954±15	90
F87A/T268V/A82T/L188I	518±41	46±1	7±1	571±43	91
F87A/T268V/A82T/I263L	534±14	43±1	16±1	593±16	90

[a] Reaction conditions: P450BM3 (0.5 μM), H_2O_2 (80 mM), Im-C6-Phe (0.5 mM), *L*-sodium ascorbate (5 mM), *p*-trifluoromethylaniline (10 mM) in pH 8.0 phosphate buffer at 25 °C. [b] All the control reactions did not show obvious activity of *p*-trifluoromethylaniline oxidation in the absence of Im-C6-Phe. [c] TON: Turnover numbers were estimated over a 30 min reaction.

Table S44. The oxidation of *p*-trifluoromethylaniline with H_2O_2 catalyzed by typical mutants of P450BM3 heme domain in the presence of Im-C6-Phe in pH 10.6 buffer.[a, b]

mutations	TON[c]			total	*1*-Nitro-4-(trifluoromethyl)benzene %
	1-Nitroso-4-(trifluoromethyl)benzene	*1*-Nitro-4-(trifluoromethyl)benzene	4,4'-Bis(trifluoromethyl)azoxybenzene		
F87A/T268V/A82T	nd[d]	651±7	15±1	666±98	98
F87A/T268V/A82T/A184V	nd[d]	1041±1	15±1	1056±2	99
F87A/T268V/A82T/L188I	nd[d]	343±12	262±12	605±24	57
F87A/T268V/A82T/I263L	nd[d]	586±5	29±1	616±6	95

[a] Reaction conditions: P450BM3 (0.5 μM), H_2O_2 (80 mM), Im-C6-Phe (0.5 mM), *p*-trifluoromethylaniline (10 mM) in pH 10.6 carbonate buffer at 25 °C. [b] All the control reactions did not show obvious activity of *p*-trifluoromethylaniline oxidation in the absence of Im-C6-Phe. [c] TON: Turnover numbers were estimated over a 30 min reaction. [d] nd: not detected.

Table S45. The oxidation of *p*-trifluoromethylaniline with H_2O_2 catalyzed by F87A/T268V/A82T/A184V mutant in the presence of Im-DFSM-dipeps in 5mM *L*-sodium ascorbate pH 8.0 buffer.[a, b]

Im-DFSM-dipeps	TON[c]			total	1-Nitroso-4-(trifluorometh
	1-Nitroso-	1-Nitro-	4,4'-		

	4-(trifluoromethyl)benzene	4-(trifluoromethyl)benzene	Bis(trifluoromethyl)azoxybenzene		yl)benzene %
Im-C6-Phe-Phe	1367±80	84±1	nd[d]	1451±81	94
Im-C6-Tyr-Nap	2195±29	78±2	nd[d]	2273±31	97
Im-C6-Phe(3CH$_3$)-Tyr	1067±30	69±1	nd[d]	1136±31	94

[a] Reaction conditions: P450BM3 (0.5 μM), H_2O_2 (80 mM), Im-DFSM-dipeps (5 μM), *L*-sodium ascorbate (5 mM), *p*-trifluoromethylaniline (10 mM) in pH 8.0 phosphate buffer at 25 °C. [b] All the control reactions did not show obvious activity of *p*-trifluoromethylaniline oxidation in the absence of Im-DFSM-dipeps. [c] TON: Turnover numbers were estimated over a 30 min reaction.

Table S46. The oxidation of *p*-trifluoromethylaniline with H_2O_2 catalyzed by F87A/T268V/A82T/A184V mutant in the presence of Im-DFSM-dipeps in pH 10.6 buffer.[a, b]

Im-DFSM-dipeps	TON[c]				*1*-Nitro-4-(trifluoromethyl)benzene %
	1-Nitroso-4-(trifluoromethyl)benzene	1-Nitro-4-(trifluoromethyl)benzene	4,4'-Bis(trifluoromethyl)azoxybenzene	total	
Im-C6-Phe-Phe	nd[d]	426±2	nd[d]	426±2	>99
Im-C6-Tyr-Nap	nd[d]	605±1	nd[d]	605±1	>99
Im-C6-Phe(3CH$_3$)-Tyr	nd[d]	242±2	nd[d]	242±2	>99

[a] Reaction conditions: P450BM3 (0.5 μM), H_2O_2 (80 mM), Im-DFSM-dipeps (5 μM), *p*-trifluoromethylaniline (10 mM) in pH 10.6 carbonate buffer at 25 °C. [b] All the control reactions did not show obvious activity of *p*-trifluoromethylaniline oxidation in the absence of Im-DFSM-dipeps. [c] TON: Turnover numbers were estimated over a 30 min reaction. [d] nd: not detected

Table S47. The oxidation of *p*-trifluoromethylaniline with H_2O_2 catalyzed by typical mutants of P450BM3 heme domain in the presence of Im-C6-Phe in pH 7.0 buffer. [a, b]

mutations	4,4'-Bis(trifluoromethyl)azobenzene TON[c]
F87L	6±1
F87L/V78S	4±1
F87L/A82L	8±1
F87L/L181Q	7±1
F87L/A184V	5±1

F87L/L188I	5±1

[a] Reaction conditions: P450BM3 (0.5 μM), H_2O_2 (40 mM), Im-C6-Phe (0.5 mM), *p*-trifluoromethylaniline (10 mM) in pH 7.0 phosphate buffer at 25 °C. [b] All the control reactions did not show obvious activity of *p*-trifluoromethylaniline oxidation in the absence of Im-C6-Phe. [c] TON: Turnover numbers were estimated over a 30 min reaction.

Table S48. The influence of free radical agent in *p*-toluidine oxidation catalyzed by F87L/V78S/A184V in the presence of Im-C6-Phe in pH 7.0 buffer.[a, b]

Time	TON[c]	
	Di-4-tolylamine	4,4'-Dimethylazobenzene
1min -[d]	38±1	537±5
1min +[d]	nd[e]	nd[e]
30min -[d]	346±18	828±40
30min +[d]	nd[e]	nd[e]

[a] Reaction conditions: F87L/V78S/A184V (0.5 μM), H_2O_2 (40 mM), Im-C6-Phe (0.5 mM), TEMPO (2mM) , *p*-toluidine (10 mM) in pH 7.0 phosphate buffer at 25 °C. [b] All the control reactions did not show obvious activity of *p*-toluidine oxidation in the absence of Im-C6-Phe. [c] TON: Turnover numbers were estimated over a 30 min reaction. [d] +: TEMPO added, -: No TEMPO added. [e] nd: not detected.

Table S49. The influence of free radical agent in *p*-toluidine oxidation catalyzed by F87A/T268V/A82T/I263L in the presence of Im-C6-Phe in pH 8.0 buffer.[a, b]

Time	TON[c]		
	4-Nitrosotoluene	4-Nitrotoluene	4,4'-Dimethylazoxybenzene
1min -[d]	166±1	nd[e]	nd[e]
1min +[d]	181±1	nd[e]	nd[e]
30min -[d]	950±8	243±3	23±1
30min +[d]	969±9	294±2	32±1

[a] Reaction conditions: F87A/T268V/A82T/I263L (0.5 μM), H_2O_2 (80 mM), Im-C6-Phe (0.5 mM), TEMPO (2 mM) , *p*-toluidine (10 mM) in pH 8.0 phosphate buffer at 25 °C. [b] All the control reactions did not show obvious activity of *p*-toluidine oxidation in the absence of Im-C6-Phe. [c] TON: Turnover numbers were estimated over a 30 min reaction. [d] +: TEMPO added, -: No

TEMPO added. [e] nd: not detected.

Table S50. Data collection and refinement statistics.[a]

**Protein**	F87L/V78S/A184V	F87A/T268V/A82T/I263L	F87A/T268V/A82T/I263L
**Ligand**	*p*-Toluidine, NH_2OH Im-C6-Phe	p-Cresol, NH_2OH Im-C6-Phe	*p*-Toluidine
**PDB Entry**	**7Y0R**	**7Y0P**	**7Y0Q**
**Data collection**			
Space group	*P 1 21 1*	*P 1 21 1*	*P 1 21 1*
Unit cell dimensions			
a, b, c (Å)	59.034, 149.907, 65.763	57.244, 144.105, 62.836	58.909, 148.689, 64.534
a, b, g (°)	90, 100.578, 90	90, 99.945, 90	90, 99.495, 90
Multiplicity	3.4 (3.6)	2.6 (2.7)	3.4 (3.6)
Completeness (%)	93.6 (99.9)	98.2 (98.4)	89.8 (99.7)
I/s_I	5.57 (2.28)	13.19 (3.36)	9.58 (2.73)
R_{merge}	0.161 (0.654)	0.056 (0.380)	0.091 (0.475)
Wilson B factor (Å^2)	28.98	29.37	40.04
$CC_{1/2}$	0.966 (0.652)	0.997 (0.751)	0.991 (0.826)
**Refinement**			
Resolution range (Å)	29.48 - 2.09 (2.17 - 2.09)	38.66 - 1.99 (2.06 - 1.99)	35.07 - 2.31 (2.39 - 2.31)
NO. reflections	65178 (6580)	67349 (6711)	42968 (4739)
R_{work}/R_{free}	0.2131/0.2392	0.1736/0.2031	0.2333/0.2492
Number of non-hydrogen atoms	7894	7873	7299
Macromolecules	7095	7079	6972

Ligands/ions	202	210	102
Solvent	597	584	225
Average B-factor	31.50	31.10	44.69
Macromolecules	31.27	30.63	44.88
Ligands	24.28	30.11	29.94
Solvent	36.65	37.14	45.65
r.m.s.d. for ideal value			
Bond length (Å)	0.006	0.019	0.019
Bond angle (°)	0.80	1.72	1.14
Ramachandran plot			
Favored/allowed/outliers (%)	96.98/3.02/0	97.20/2.80/0	97.26/2.74/0

[a]Values for the highest resolution shell are given in parentheses.

Supporting Methods

Materials

All chemical reagents were purchased from commercial sources (e.g. Aldrich, TCI, and Alladin) and used without further purification until otherwise noticed. Dual-functional small molecule (DFSM), *N*-(ω-imidazolyl)-hexanoyl-L-phenylalanine (Im-C6-Phe) was synthesized according to our previous report. [1]

Expression and Purification of P450BM3 Enzymes

The heme domain of Cytochrome P450BM3 and its variants were respectively cultured and purified according to reported methods. [1] All of purified proteins were characterized by SDS pages.

The formation of a ferrous CO complex was confirmed by UV-visible spectral change through the reduction of ferric heme of the wild type P450 and its mutants by addition of $Na_2S_2O_4$ in the presence of carbon monoxide (CO). [2]

The concentrations of P450BM3 and its variants were measured by Hemochrome binding assay. [3] A pyridine solution was made by combining pyridine (1.75 mL) and 1 M aqueous of NaOH (0.75 mL). The solution was mixed at room temperature then centrifuged for 30 s at 5000 rpm to remove excess aqueous base. To a cuvette containing 0.75 mL of protein solution in phosphate buffer (0.1 M, pH 8.0), 0.25 mL of the pyridine solution was added followed by 2 mg of sodium dithionite. A UV-vis spectrum was recorded immediately. Hemoprotein concentration was determined from the absorbance of the hemochrome complex using extinction coefficients of ε_{418} = 196 mM^{-1} cm^{-1}. Absorbance was assigned as the difference between the peak max at 418 nm and the baseline at 420 nm as determined by extrapolating from two points on either side of the hemochrome peak (390 nm and 450 nm).

Mutagenesis and Recombination

All the mutations were made by PCR based site-directed mutagenesis and verified by DNA sequencing. The single mutants at the position of F87 were prepared according to previous report. [1]

The primers used were as follows:

mutation	sequence
F87A-F	5’- GCGACAAGCTGGACGCATGAAAAAAATTG -3’
F87G-F	5’- GGTACAAGCTGGACGCATGAAAAAAATTGGAA -3’
F87V-F	5’- GTGACAAGCTGGACGCATGAAAAAAATTGGAA -3’
F87I-F	5’- ATTACAAGCTGGACGCATGAAAAAAATTGAAAAAAGC -3’
F87L-F	5’- CTGACAAGCTGGACGCATGAAAAAAATTGGAA -3’
F87M-F	5’- ATGACAAGCTGGACGCATGAAAAAAATTGGAA -3’
F87P-F	5’- CCGACAAGCTGGACGCATGAAAAAAATTGGAA -3’
F87S-F	5’- AGCACAAGCTGGACGCATGAAAAAAATTGGAA -3’
F87C-F	5’- TGCACAAGCTGGACGCATGAAAAAAATTGGAA -3’
F87T-F	5’- ACCACAAGCTGGACGCATGAAAAAAATTGGAA -3’
F87D-F	5’- GATACAAGCTGGACGCATGAAAAAAATTGGAA -3’
F87N-F	5’- AATACAAGCTGGACGCATGAAAAAAATTGGAA -3’
F87Q-F	5’- CAGACAAGCTGGACGCATGAAAAAAATTGGAA -3’
F87E-F	5’- GAAACAAGCTGGACGCATGAAAAAAATTGGAA -3’
F87H-F	5’- CATACAAGCTGGACGCATGAAAAAAATTGGAA -3’
F87R-F	5’- CGTACAAGCTGGACGCATGAAAAAAATTGGAA -3’
F87K-F	5’- AAAACAAGCTGGACGCATGAAAAAAATTGGAA -3’
F87-R	5’- TAACCCGTCTCCTGCAAAATCACGTACAA -3’

The F87A/T268 and F87L/T268 double mutants were prepared used F87A and F87L mutants as parent templates. The primers used were as follows:

primer	sequence
T268G-F	5’- GGTACAAGTGGTCTTTTATCATTTGC -3’
T268A-F	5’- GCGACAAGTGGTCTTTTATCATTTGC -3’
T268V-F	5’- GTGACAAGTGGTCTTTTATCATTTGC -3’
T268I-F	5’- ATCACAAGTGGTCTTTTATCATTTGC -3’
T268L-F	5’- CTGACAAGTGGTCTTTTATCATTTGC -3’
T268F-F	5’- TTCACAAGTGGTCTTTTATCATTTGC -3’
T268P-F	5’- CCGACAAGTGGTCTTTTATCATTTGC -3’
T268S-F	5’- AGCACAAGTGGTCTTTTATCATTTGC -3’
T268C-F	5’- TGCACAAGTGGTCTTTTATCATTTGC -3’
T268-R	5’- TTCGTGTCCCGCAATTAAGAATG -3’

The F87A/T268V/X triple mutants and F87L/X double mutants were prepared used F87A/T268V mutant and F87L mutant as parent templates, respectively. Beneficial mutations selected from the prepared mutations were recombined. All primers used were as follows:

primer	sequence
L75V-F	5’- CGGTTAAATTTGTACGTGATTTTGCAGGAGAC -3’
L75A-F	5’- CGGCAAAATTTGTACGTGATTTTGCAGGAGAC -3’
L75-R	5’- CTTGACTTAAGTTTTTATCAAAGCGTGATTCATCG -3’
V78L-F	5’- CTTAAATTTCTGCGTGATTTTGCAGGAGACG -3’
V78M-F	5’- CTTAAATTTATGCGTGATTTTGCAGGAGACG -3’

V78I-F	5'- CTTAAATTTATTCGTGATTTTGCAGGAGACG -3'
V78A-F	5'- CTTAAATTTGCGCGTGATTTTGCAGGAGACG -3'
V78S-F	5'- CTTAAATTTAGCCGTGATTTTGCAGGAGACG -3'
V78C-F	5'- CTTAAATTTTGCCGTGATTTTGCAGGAGACG -3'
V78T-F	5'- CTTAAATTTACCCGTGATTTTGCAGGAGACG -3'
V78G-F	5'- CTTAAATTTGGTCGTGATTTTGCAGGAGACG -3'
V78F-F	5'- CTTAAATTTTTTCGTGATTTTGCAGGAGACG -3'
V78-R	5'- CGCTTGACTTAAGTTTTTATCAAAGCGTGAT -3'
A82G-F	5'- GATTTTGGTGGAGACGGGTTA -3'
A82F-F	5'- GATTTTTTTGGAGACGGGTTA -3'
A82T-F	5'- GATTTTACCGGAGACGGGTTA -3'
A82A-F	5'- GATTTTGCAGGAGACGGGTTA -3'
A82V-F	5'- GATTTTGTTGGAGACGGGTTA -3'
A82L-F	5'- GATTTTCTGGGAGACGGGTTA -3'
A82M-F	5'- GATTTTATGGGAGACGGGTTA -3'
A82S-F	5'- GATTTTAGCGGAGACGGGTTA -3'
A82I-F	5'- GATTTTATTGGAGACGGGTTA -3'
A82C-F	5'- GATTTTTGTGGAGACGGGTTA -3'
A82K-F	5'- GATTTTAAAGGAGACGGGTTA -3'
A82-R	5'- ACGTACAAATTTAAGCGCTTGAC -3'
L181V-F	5'- GTGCAGTGGATGAAGCAATGAACAAGCTG -3'
L181I-F	5'- GTGCAATTGATGAAGCAATGAACAAGCTG -3'
L181M-F	5'- GTGCAATGGATGAAGCAATGAACAAGCTG -3'
L181Q-F	5'- GTGCACAGGATGAAGCAATGAACAAGCTG -3'
L181F-F	5'- GTGCATTTGATGAAGCAATGAACAAGCTG -3'
L181K-F	5'- GTGCAAAAGATGAAGCAATGAACAAGCTG -3'
L181A-F	5'- GTGCAGCAGATGAAGCAATGAACAAGCTG -3'
L181T-F	5'- GTGCAACCGATGAAGCAATGAACAAGCTG -3'
L181-R	5'- GGACCATACTTGTAATAAATGGATGAGGCT-3'
A184I-F	5'- CACTGGATGAAATTATGAACAAGCTG -3'
A184V-F	5'- CACTGGATGAAGTGATGAACAAGCTG -3'
A184L-F	5'- CACTGGATGAACTGATGAACAAGCTG -3'
A184G-F	5'- CACTGGATGAAGGTATGAACAAGCTG -3'
A184M-F	5'- CACTGGATGAAATGATGAACAAGCTG -3'
A184K-F	5'- CACTGGATGAAAAAATGAACAAGCTG -3'
A184R-F	5'- CACTGGATGAACGTATGAACAAGCTG -3'
A184-R	5'- CACGGACCATACTTGTAATAAATGGATGAG -3'
L188I-F	5'- ATTCAGCGAGCAAATCCAGACG -3'
L188V-F	5'- GTTCAGCGAGCAAATCCAGACG -3'
L188A-F	5'- GCACAGCGAGCAAATCCAGACG -3'
L188M-F	5'- ATGCAGCGAGCAAATCCAGACG -3'
L188F-F	5'- TTTCAGCGAGCAAATCCAGACG -3'
L188-R	5'- CTTGTTCATTGCTTCATCCAGTG -3'

I263L-F	5'- ATTCTTACTGGCGGGACACGA -3'
I263V-F	5'- ATTCTTAGTGGCGGGACACGA -3'
I263M-F	5'- ATTCTTAATGGCGGGACACGA -3'
I263T-F	5'- ATTCTTAACGGCGGGACACGA -3'
I263G-F	5'- ATTCTTAGGGGCGGGACACGA -3'
I263A-F	5'- ATTCTTAGCGGCGGGACACGA -3'
I263Q-F	5'- ATTCTTACAGGCGGGACACGA -3'
I263-R	5'- GTAATAATTTGATAGCGAATGTTCTCGTC -3'
A264E-F	5'- GAAGGACACGAAGTGACAAGTGG -3'
A264M-F	5'- ATGGGACACGAAGTGACAAGTGG -3'
A264Q-F	5'- CAGGGACACGAAGTGACAAGTGG -3'
A264S-F	5'- AGCGGACACGAAGTGACAAGTGG -3'
A264V-F	5'- GTTGGACACGAAGTGACAAGTGG -3'
A264I-F	5'- ATTGGACACGAAGTGACAAGTGG -3'
A264-R	5'- GATCAGGAAGGTAATAATTTGATAGCG -3'
E267D-F	5'- GATACAACAAGTGGTCTTTTATCA -3'
E267Q-F	5'- CAGACAACAAGTGGTCTTTTATCA -3'
E267S-F	5'- AGCACAACAAGTGGTCTTTTATCA -3'
E267L-F	5'- CTGACAACAAGTGGTCTTTTATCA -3'
E267-R	5'- GTGTCCCGCAATTAAGAATGT -3'
A328V-F	5'- GTGCCTGCGTTTTCCCTATATGC -3'
A328-R	5'- AGTTGGCCATAAGCGCAGC -3'

General procedure for *p*-substituted aniline oxidation

P450BM3 heme domain mutant (0.5 μM) was transferred to a glass sample bottle containing 0.1 M, pH 7.0, 8.0 or 10.6 buffer, *p*-substituted aniline (10 mM, dissolved in DMSO (2% vol/vol)) and Im-C6-Phe (500 μM, dissolved in pH 8.0 or 7.0 phosphate buffer) or Im-DFSM-dipeps (5 μM, dissolved in pH 8.0 or 7.0 phosphate buffer). The reaction was initiated by the addition of H_2O_2 (20/40/60/80 mM, dissolved in pH 7.0, 8.0 or 10.6 buffer). The reaction mixture was incubated in water bath at 25 °C for 30 min. After the reaction, the mixture was quenched and extracted with 1 mL of ethyl acetate, and the organic phase was separated and dried with sodium sulphate anhydrous. The product was analyzed by gas chromatography (GC) by benzophenone as an internal standard. Control reactions performed by repeating these steps in the absence of DFSMs.

General procedure for Semi-preparative scale synthesis of *p*-substituted aniline oxidation product by the DFSM-facilitated P450BM3 peroxygenase system.

One-electron oxidation

0.5 μM heme domain of F87L/V78S/A184V was transferred to a glass flask containing 50 mL 0.1 M pH 7.0 phosphate buffer, 8 mM *p*-toluidine (dissolved in 2% DMSO) and 0.5 mM Im-C6-Phe. The reaction was initiated by the addition of 40 mM H_2O_2 and incubated at 25 ºC. 0.5 μM enzyme was added to the reaction system every 5 minutes for 2 times. The total reaction time was 1 h. The reaction mixture was extracted with ethyl acetate (50 mL × 3), and the combined organic phase was dried with anhydrous sodium sulphate, filtered and concentrated. The mixture was separated and purified with neutral alumina preparation plate (toluene: petroleum ether = 2: 1) to

give Di-4-tolylamine (4 mg), 4,4'-Dimethylazobenzene (13 mg), N, N'-Di-p-tolyl-5-amino-2-methyl-2, 5-cyclohexadiene-1, 4-diimine (9.9 mg). The other one electron oxidation products of *p*-substituted anilines were also synthesized by the same protocol.

***p*-nitrosotoluene**

The reaction was performed with the most active F87A/T268V/A82V/L188I mutant (2 μM), *p*-toluidine (2 mM), Im-C6-Phe-Phe (5 μM), *L*- sodium ascorbate (5 mM) and H_2O_2 (80 mM, affter 5 minutes add 30 mM) in 100 mL 0.1 M pH 8.0 phosphate buffer at 25°C. The reaction mixture was extracted with ethyl acetate (100 mL × 3), and the combined organic phase was dried with anhydrous sodium sulphate, filtered and concentrated. The mixture was separated and purified with silica gel (ethyl acetate: petroleum ether = 1: 8) to give *p*-nitrosotoluene as a colorless solid (17.2 mg, 71%).

***p*-Br-nitrobenzene**

The optimal reaction condition was F87A/T268V/A82T/A184V (6 μM), *p*-Br-aniline (4 mM), Im-C6-Phe-Phe (5 μM) and H_2O_2 (100 mM) in 50 mL 0.1 M pH 10.6 carbonate buffer at 25°C. The reaction mixture was extracted with ethyl acetate (50 mL × 3), and the combined organic phase was dried with anhydrous sodium sulphate, filtered and concentrated. The mixture was separated and purified with silica gel (ethyl acetate: petroleum ether = 1: 8) to give *p*-Br-nitrobenzene as a colorless solid (30.4 mg, 77%).

General procedure for preparation of *p*-nitrobenzene compounds (1-Bromo-4-nitrobenzene as an example)

A solution of oxone (1.79 g, 2.9 mmol) in H_2O (15 mL) was gradually added over a 20-minute period to a solution of *p*-bromoaniline (0.5 g, 2.9 mmol) in H_2O under a nitrogen atmosphere. Following stirring under nitrogen at room temperature for 2 hours, the solution was extracted with dichloromethane (DCM). The combined organic extracts were washed successively with 1 M HCl, saturated sodium bicarbonate, brine, and water, then dried over sodium sulfate. After filtration, the solvent was removed to yield the crude products. The crude products were purified further by column chromatography using a ethyl acetate/hexane ratio of 1:8, and the resulting product was characterized by ^{1}H NMR.

General procedure for preparation of azo compound (4,4'-Difluoroazobenzene as an example)

A suspension of iodine powder (3.43 g, 13.5 mmol) in toluene (40 ml) was prepared, and *p*-fluoroaniline (1 g, 9 mmol) was added, followed by NaOH pellets (1.08 g, 27 mmol) under a nitrogen atmosphere. Following a vigorous reaction, the mixture was filtered, and the residue was washed with DCM and diluted HCl. The organic layer was separated from the filtrate and washed with diluted HCl and H_2O, then dried over Na_2SO_4. The solvent was removed to yield the crude products, which was further purified by column chromatography using a DCM/hexane ratio of 1:15. Similarly, other azo compound products were synthesized using the same protocol. The compounds were characterized by ^{1}H NMR.

General procedure for preparation of azoxy compound (4,4'-Dimethylazoxybenzene as an example)

4,4'-Dimethylazobenzene (100 mg, 0.48 mmol) was dissolved in glacial acetic acid at 80 ºC, to which 8 ml of 30% H_2O_2 was subsequently added. Further additions of 8 ml of 30% H_2O_2 were made every 15 minutes during the initial 2 hours, followed by an additional 20 ml. The reaction

mixture was maintained at approximately 65 ºC. After 3 hours, 12 ml of 30% H_2O_2 was added, followed by another 10 ml after 20 hours. Heating continued until thin-layer chromatography (TLC) analysis indicated the complete disappearance of the starting azo compound, which occurred after 26 hours. Subsequently, the solution was allowed to cool to room temperature and was poured into 300 ml of excess H_2O. The precipitate was filtered, dried, and crystallized twice using ethanol. The compounds were characterized by ^{1}H NMR.

Instruments and Analytical Conditions

Gas Chromatography

After the reaction, the reaction mixture was extracted with 1 mL of ethyl acetate, and the organic phase was separated and dried with sodium sulphate anhydrous. The product analysis was performed on a Shimadzu GC-2010 plus gas chromatograph equipped with a DB-5 column (length: 30 m, internal diameter: 0.25 mm, film thickness: 1.0 µm, Agilent, USA), a flame ionization detector, and an AOC-20i auto sampler system. The analytical conditions were as follows:

splitting ratio: 1/9, temperature program: injector 280 ºC, detector 300 ºC, 120 ºC oven , then 30 ºC/min gradient to 140 ºC for 2 min, 40ºC /min gradient to 240 ºC, then 10 ºC/min gradient to 290 ºC for 5 min (total 15.17 min).

Crystallization of the P450BM3 mutants in complex with the DFSM and substrates

The affinity column purified proteins were subjected to gel filtration using a HiPrep 16/60 Sephacryl S-200 column (Cytiva), followed by concentration and storage in 10 mM Tris buffer (pH 8.0) prior to crystallization. The crystallization experiment was performed at 18 °C using hanging drop evaporation combined with seeding as described previously.[3] Briefly, multi-crystals of F87A mutant in complex with Im-C6-Phe were prepared by mixing 1 ml protein solution (30 mg/ml protein supplemented with 1 mM Im-C6-Phe) and 1 ml reservoir solution A (0.1 M Tris pH 8.0, 0.38 M $MgCl_2$ and 28% PEG 3350). The multi-crystals were crushed into microcrystals and diluted 1000-fold with reservoir solution A as the seeding solution. The rod-like single crystals of F87L/V78S/A184V and F87A/T268V/A82T/I263L mutants in complex with Im-C6-Phe were obtained within 1 week by mixing 1.5 ml protein solution, 1 ml reservoir solution B (0.1 M Tris pH 8.5, 0.38 M $MgCl_2$, and 10-20% PEG 3350), and 0.5 ml seeding solution. The crystals were soaked in a cryo-protecting solution containing 0.1 M Tris pH 8.5, 0.38 M $MgCl_2$, 16% PEG 3350, 1mM Im-C6-Phe, 20% glycerol, 0.5 M hydroxylamine, 5-100 mM *p*-toluidine or 100 mM p-cresol for 3 min before transferring into the liquid nitrogen for data collection.

Data Collection and Structure Determination

The X-ray diffraction data were collected at BL02U1 and BL10U2 beamlines of the Shanghai Synchrotron Radiation Facility (SSRF) with a DECTRIS EIGER X 16M detector at 100 K and processed with Aquarium.[4] The structure was solved by molecular replacement with PHENIX Phaser-MR,[5]using the previously determined structure of P450BM3 heme domain (PDB ID: 7EGN) as the search model. The structure model was further built using COOT[6] and refined by PHENIX Refine.[7] The overall quality of the structural model was checked by MolProbity,[8] and the structure has been deposited in the Protein Data Bank (PDB ID: 7Y0P, 7Y0Q&7Y0R). Data collection and refinement statistics are shown in Table S35. Protein structure graphics were generated by PyMOL (https://www.schrodinger.com).

Supporting References

[1] N. Ma, Z. Chen, J. Chen, J. Chen, C. Wang, H. Zhou, L. Yao, O. Shoji, Y. Watanabe, Z. Cong, *Angew. Chem. Int. Ed*. **2018**, *57*, 7628-7633; *Angew. Chem.* **2018**, *130*, 7754-7759.

[2] T. Omura, R. Sato, *J. Biol. Chem*. **1964**, *239*, 2379-2385.

[3] X. Wang, X. Lin, Y. Jiang, X. Qin, N. Ma, F. Yao, S. Dong, C. Liu, Y. Feng, l. Jin, M. Xian, Z. Cong, *Angew. Chem. Int. Ed*. **2023**, *62*, e202217678; *Angew. Chem.* **2023**, *135*, e202217678.

[4] F. Yu, Q. Wang, M. Li, H. Zhou, K. Liu, K. Zhang, Z. Wang, Q. Xu, C. Xu, Q. Pan, J. He, *J. Appl. Crystallogr*. **2019**, *52*, 472-477.

[5] P. D. Adams, P. V. Afonine, G. Bunkoczi, V. B. Chen, I. W. Davis, N. Echols, J. J. Headd, L. W. Hung, G. J. Kapral, R. W. Grosse-Kunstleve, A. J. McCoy, N. W. Moriarty, R. Oeffner, R. J. Read, D. C. Richardson, J. S. Richardson, T. C. Terwilliger, P. H. Zwart, *Acta Crystallogr., Sect. D: Biol. Crystallogr*. **2010**, *66*, 213-221.

[6] P. Emsley, B. Lohkamp, W. G. Scott, K. Cowtan, *Acta Crystallogr., Sect. D: Biol. Crystallogr*. **2010**, *66*, 486-501.

[7] N. Echols, N. W. Moriarty, H. E. Klei, P. V. Afonine, G. Bunkóczi, J. J. Headd, A. J. McCoy, R. D. Oeffner, R. J. Read, T. C. Terwilliger, P. D. Adams, *Acta Crystallogr. D Biol. Crystallogr*. **2014**, *70*, 144–154.

[8] V. B. Chen, W. B. Arendall 3rd, J. J. Headd, D. A. Keedy, R. M. Immormino, G. J. Kapral, L. W. Murray, J. S. Richardson, D. C. Richardson, *Acta Crystallogr. D Biol. Crystallogr*. **2010**, *66*, 12–21.
